# Overriding Stereochemical Outcomes in Cyclase Phase Total Synthesis: Enantioselective Synthesis of Habiterpenol and Dasyscyphin A

**DOI:** 10.1021/jacs.6c00141

**Published:** 2026-02-11

**Authors:** Licheng Wu, Long H. Nguyen, Liwen Yan, Haoyu Yin, Natsuki Mizuno, Alexander W. Schuppe

**Affiliations:** Department of Chemistry, 5718 Vanderbilt University, Nashville, Tennessee 37235, United States

## Abstract

Herein, we report the enantioselective total syntheses of habiterpenol and dasyscyphin A. By exploiting a quaternary carbon stereocenter epimerization protocol, we could override the intrinsic stereochemical bias of polyolefin cyclization reactions. Accordingly, we achieved the efficient and selective construction of the polycyclic scaffolds of both meroterpenoid natural products that bear an unconventional *cis*-hydrindane motif. This work demonstrates the utility of stereocenter remodeling in reprogramming the outcomes of biomimetic cyclizations, thus enabling the rapid synthesis of terpenoid frameworks that deviate from the prototypical all-*trans*-ring fusions.

## Graphical abstract

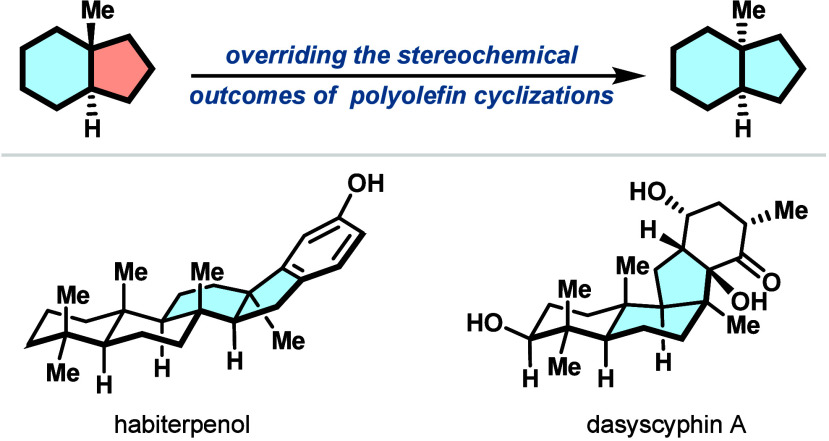

Over the past half-century, synthetic chemists have drawn insight from the exquisite selectivity of cyclase phase biosynthetic pathways that transform linear olefin precursors into complex polycyclic architectures characteristic of many natural product classes.^1^ Radical and polar polyolefin cyclization reactions are frequently employed in total synthesis owing to their capacity to efficiently assemble stereochemically complex scaffolds with high selectivity (Scheme 1A).^2^ As the polyprenoid substrates for these reactions are *E*-olefins, the concerted nature of the reaction mechanism and the conformational preorganization of linear polyenes afford exclusively *trans*-fused carbocycles.^3^ While this preference closely mirrors the biosynthetic cyclase phase, it also imposes a significant retrosynthetic constraint: polyolefin cyclizations are generally not applicable for synthesizing natural products that do not conform to this stereochemical pattern. As a result, terpenoids featuring *cis*-fused hydrindanes must be assembled through alternative stepwise approaches.^4^

**Scheme 1:**
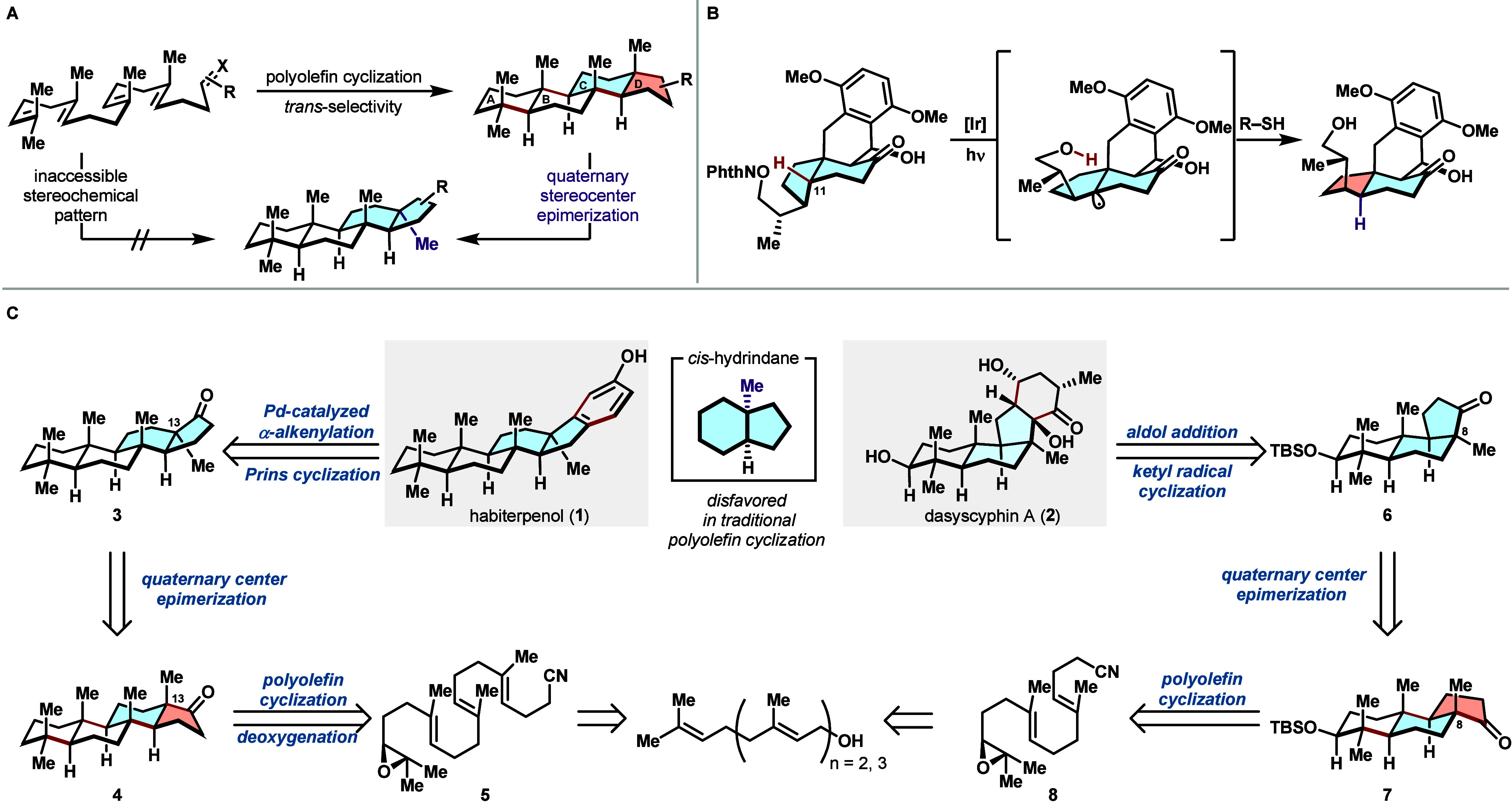
Overriding Stereochemical Outcomes in Cyclase Phase Total Synthesis

Stereocenter remodeling, modifying specific stereocenters after a bond-forming step, has emerged as a powerful tool in natural product total synthesis to tackle this limitation. These reactions allow chemists to circumvent inherent selectivity biases and access stereoisomers that are otherwise difficult to obtain through primary bond-forming reactions. While this approach has traditionally entailed the stereochemical inversion of secondary alcohols or epimerization at carbonyl α-positions through deprotonation, recent advances using radical intermediates have expanded this concept to include the epimerization of unactivated tertiary carbons.^5–7^ For example, Sorensen addressed the epimerization of a tertiary carbon center in the synthesis of pleurotin (Scheme 1B).^7a^ To adjust the stereochemistry of C11, a tertiary carbon-centered radical was generated through an intramolecular 1,5-hydrogen atom transfer (HAT) from an intermediate alkoxy radical. A subsequent HAT with thiol furnished the epimerized product. Furthermore, this tertiary C–H epimerization strategy has recently been leveraged for the concise synthesis of several alkaloid natural products by reconfiguring the native topology of abundant steroid precursors.^7b,7c^

However, such epimerization processes are not amenable to quaternary carbon centers, particularly those formed through polyolefin cyclization reactions. Recently, our group developed a transformation for the epimerization of quaternary carbon centers adjacent to ketones via a transient imine intermediate.^8^ This process proceeds through photochemical excitation of the imine substructure to produce a diradical intermediate, followed by β-scission, stereochemical inversion of the tertiary radical species, and radical recombination to form the epimer with high selectivity. Our continued interest in stereocenter modification led us to apply this α-epimerization transformation toward the outstanding synthetic challenge of overriding conventional stereochemical outcomes in cyclase phase synthesis.

To demonstrate the feasibility of perturbing prototypical polyolefin cyclization selectivity (Scheme 1C), we identified two structurally distinct microbial secondary metabolites, habiterpenol (**1**) and dasyscyphin A (**2**). Both molecules contain a *cis*-hydrindane embedded in the core structure and thus would present an impediment to direct access via biomimetic polyolefin cyclization. Habiterpenol, a meroterpenoid, is a noncytotoxic G2-checkpoint inhibitor characterized by a highly substituted phenol.^9^ Prior total syntheses of habiterpenol utilized sclareolide as a building block and demonstrated that a key challenge was constructing the C13 quaternary carbon center in a stereocontrolled manner.^10^ Furthermore, dasyscyphin A is a fungal merosesquiterpene and the most densely functionalized congener among the dasyscyphin natural product family, featuring a stereochemically rich tricyclic framework fused to a highly oxygenated D-ring derived from phenolic unit.^11^ While dasyscyphin A has not previously been synthesized, we anticipated that stereoselective synthesis of the tricyclic 6/6/5 terpenoid architecture, featuring an unconventional *trans-anti-cis* stereochemical array and a densely functionalized D-ring, would present major obstacles.^12^ Sarlah and co-workers recently reported a concise synthesis of dasyscyphin B via a Au-catalyzed Rautenstrauch rearrangement, demonstrating complementary access to the tricyclic architecture through catalyst-controlled cyclization strategies.^12d^ By strategically deploying our quaternary carbon center epimerization protocol, we envisioned that we could rapidly construct these terpenoid frameworks in a stereoselective manner, thereby addressing the synthetic challenges inherent to both molecules.

Retrosynthetically, we envisioned that habiterpenol could be disconnected from its substituted phenolic ring. This could arise from an annulative Prins cyclization and aromatization, thereby simplifying the structure back to a *cis*-fused hydrindane core (**3**). Precise modulation of the C13 stereocenter via epimerization would lead to **4** as a key intermediate, which could be assembled through a biomimetic polyolefin cyclization of a derivative of geranylgeraniol. Similarly, dasyscyphin A, bearing a highly functionalized D-ring, can be traced back to an analogous *cis*-hydrindane framework (**6**) through an annulative sequence featuring aldol addition and reductive radical cyclization. This *trans-anti-cis* substructure can be accessed via quaternary stereocenter epimerization from the all-*trans* polycyclic scaffold **7**. Retrosynthetic deconstruction of the polycyclic framework traces back to linear polyene **8**, a precursor readily accessible from farnesol. Together, this epimerization logic would enable the facile construction of the nontraditional polycyclic terpenoid framework from simple linear starting materials.

We began our first-generation synthesis of habiterpenol by forging the tetracyclic core (**4**) through a cationic polyolefin cyclization (Scheme 2A). Treatment of (*E*,*E*)-geranyllinalool (**9**) with PBr_3_ produced allylic bromide **10** in 99% yield and a mixture of olefin isomers (3:1 *E*/*Z*). A substitution reaction of **10** with lithiated 1-(trimethylsilyl)­propyne provided alkyne **11** after in situ removal of the silyl group. Subsequent alkylation of the acetylenic position with (iodomethyl)­trimethylsilane formed polyolefin cylization precursor **12**. A Brønsted-acid-mediated cationic polyene cyclization delivered an allene intermediate, which upon ozonolysis generated **4** in trace yield (<5%).^3d^ While we were able to rapidly synthesize the tetracyclic core of habiterpenol through this route, poor material throughput and difficulty in adapting it to an enantioselective route led us to pursue an alternative sequence.

**Scheme 2:**
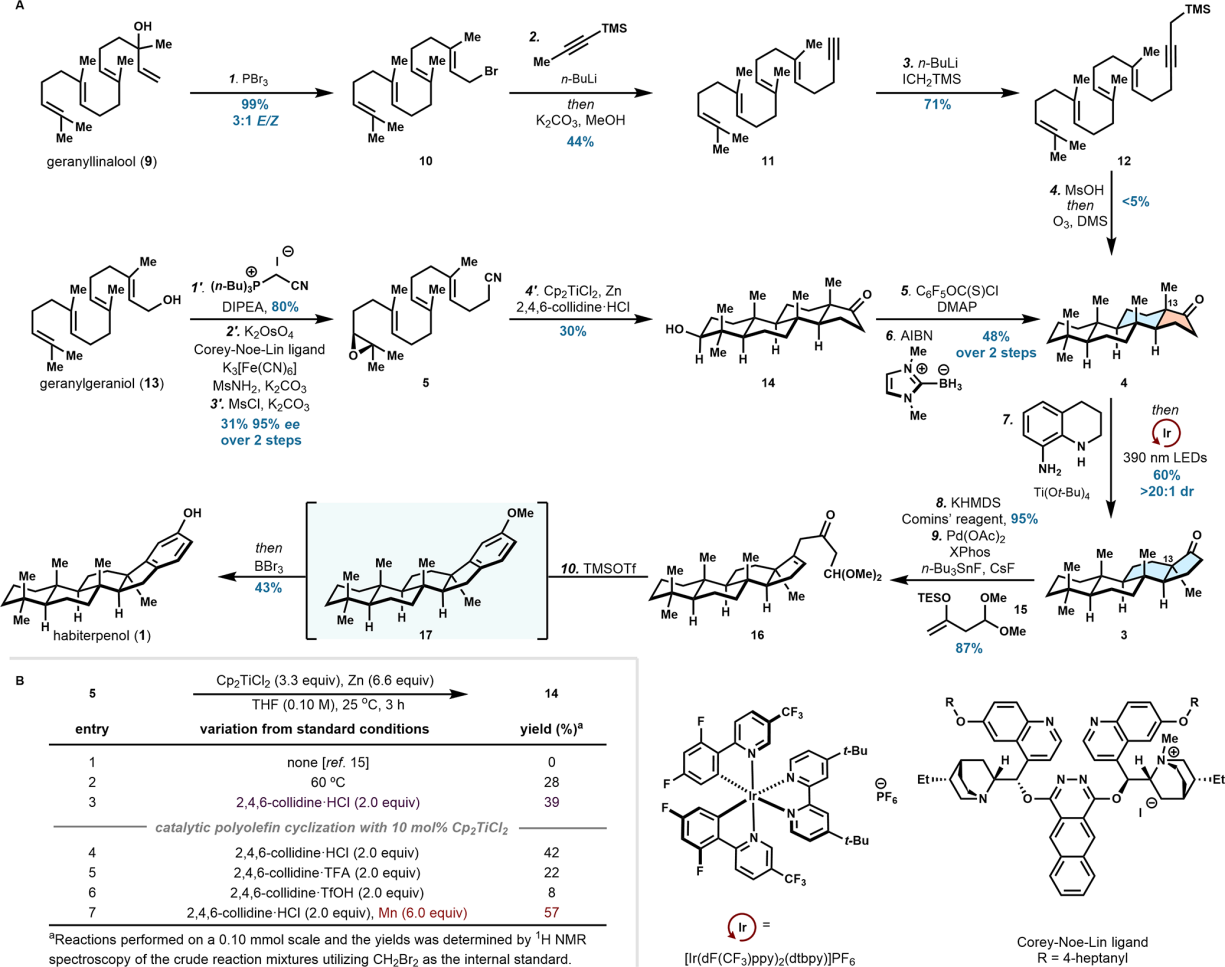
Total Synthesis of Habiterpenol

Accordingly, our enantioselective synthesis of **4** commenced by converting commercially available (*E*,*E*,*E*)-geranylgeraniol (**13**) to **14**. Although we established a stepwise protocol to access geranylgeranyl acetonitrile from **13** (see the Supporting Information for details), we sought to develop a more efficient method. To this end, we established a direct two-carbon homologation of the allylic alcohol, delivering the γ,δ-unsaturated nitrile in excellent yield using Zaragoza’s method, which employs (cyanomethyl)­tributylphosphonium iodide.^13^ Notably, this procedure obviates the need for an unstable allylic bromide precursor or pyrophoric reagents. Synthesis of the enantioenriched terminal epoxide was accomplished through Os-catalyzed asymmetric dihydroxylation, utilizing Corey-Noe-Lin ligand, and subsequent epoxidation through the intermediacy of the mesylate formed **5** in 31% yield over two steps and 95% *ee*.^14^

With a robust approach to epoxynitrile **5** established, we then focused on the key radical polyolefin cyclization to generate **4**. Fernández-Mateos previously reported that **5** resulted exclusively in reductive ring-opening of the epoxide under Ti-mediated cyclization conditions (entry 1, Scheme 2B).^15^ Undeterred, we systematically evaluated a range of the reaction parameters. While the reported conditions failed to deliver detectable product, elevating the reaction temperature led to increased formation of the desired cyclized species (entry 2) and the use of 2,4,6-collidine hydrochloride yielded **14** at ambient temperature (entry 3). In line with prior mechanistic investigations, this additive likely stabilizes the active Ti­(III) species by suppressing catalyst deactivation.^16^ Further optimization revealed that catalytic quantities of the Ti species remained effective (entries 4–6) and the use of Mn as a heterogeneous reductant resulted in the highest yield of **14** on a small scale (entry 7). However, the poor dispersibility of Mn powder rendered this process impractical on a multigram scale, necessitating the use of Zn as an alternative. The modest yield of this process is a consequence of challenging construction of the tetracyclic framework, and results in ring-opening and partially cyclizes species as byproducts.

The secondary alcohol was then excised via an initial conversion to the *O*-(perfluorophenyl) thiocarbonate. Boryl-radical-mediated deoxygenation employing conditions developed by Curran afforded **4** in excellent yield.^17^ To revise the configuration of the D-ring, the stereochemistry of C13 was modified by utilizing our photochemical epimerization protocol. Ketone **4** was first condensed with 1,2,3,4-tetrahydroquinolin-8-amine to afford an intermediate imine. Treatment of the corresponding imine with catalytic [Ir­(dF­(CF_3_)­ppy)_2_(dtbpy)]­PF_6_ (**Ir**) as a photosensitizer and 390 nm LEDs resulted in epimerized ketone **3** in excellent yield and diastereoselectivity (>20:1 dr) after acidic imine hydrolysis. This epimerization proceeds through a dynamic equilibrium, ultimately favoring formation of the more thermodynamically stable isomer. Our epimerization strategy not only offers a rapid and selective route toward the terpenoid skeleton but also provides a novel retrosynthetic tactic, decoupling ring formation and quaternary stereocenter construction.

The final structural element in **1** that remained to be appended was the E-ring phenol, for which we envisioned a stepwise annulation to achieve this objective. The ketone was first converted to the corresponding vinyl triflate, after which the sp^2^–sp^3^ C–C bond of **16** was forged via a Pd-catalyzed carbonyl α-alkenylation using conditions developed by Kapur.^18^ Further treatment of **16** with TMSOTf facilitated a Prins cyclization and aromatization to surprisingly deliver the methylated phenolic intermediate (**17**).^19^ At this juncture we cannot rule out that a 6π electrocyclization mechanism is operative, which would proceed through a discrete triene species. We speculate that formation of the ethereal linkage likely occurs prior to the formation of the phenol substructure, as resubjection of **1** to analogous reaction conditions did not produce **17** (see the Supporting Information for details). Subsequent in situ demethylation with BBr_3_ smoothly unveiled **1**, thereby completing our enantioselective total synthesis of habiterpenol in 10 steps.

We sought to further demonstrate the utility of our epimerization protocol to precisely manipulate the stereochemical patterns of polyolefin cyclization transformations. As such, our synthesis of dasyscyphin A initiated with conversion of farnesol (**18**) to farnesyl acetonitrile (**19**) employing an analogous Zaragoza homologation reaction (Scheme 3). Sequential asymmetric dihydroxylation of the terminal olefin and formation of the enantioenriched epoxide generated cyclization precursor **8** in excellent yield and selectivity (97% *ee*). Ti-catalyzed polyolefin cyclization and *tert*-butyldimethylsilyl (TBS) protection of the ensuing secondary alcohol provided **7** with the all-*trans* configuration. We were able to smoothly adjust the stereochemistry at C8 with our epimerization protocol, and the tricyclic core (**6**) of dasyscyphin A was accessed in 69% yield and >20:1 dr.

**Scheme 3:**
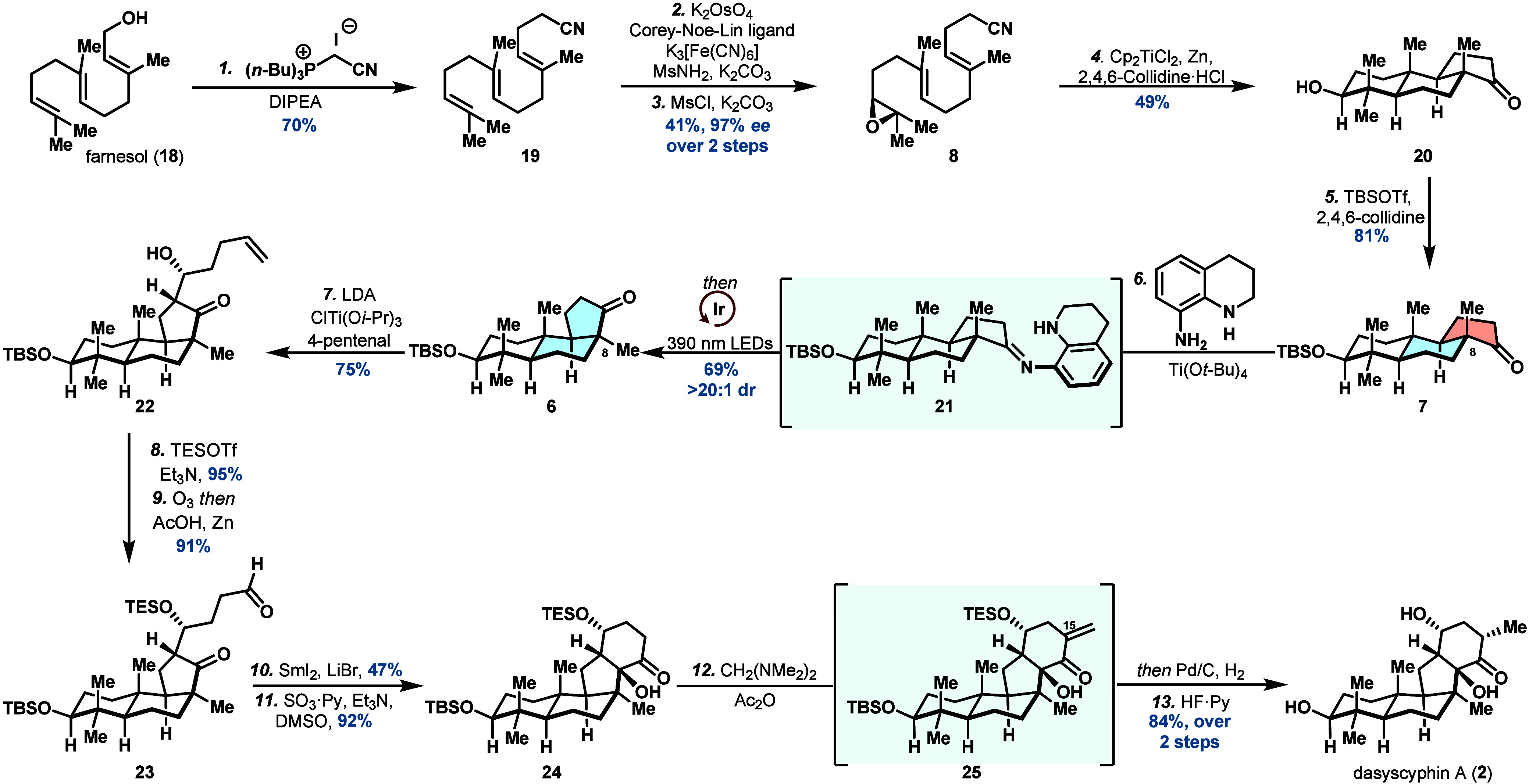
Total Synthesis of Dasyscyphin A

We then turned our attention toward construction of the highly functionalized D-ring present in **2**, which proved to be a formidable challenge (see the Supporting Information for details). A Ti-mediated diastereoselective aldol addition generated alcohol **22**, followed by conversion to the corresponding silyl ether. Ozonolysis of the pendant alkene produced aldehyde **23** and set the stage for reductive cyclization. Our preliminary investigations revealed that the SmI_2_-mediated ketyl radical cyclization was more difficult than anticipated. This was likely due to the sterically encumbered ketone and predominantly led to aldehyde reduction and dimerization. Optimization revealed that using the PhMe as solvent suppresses the competing reduction of aldehyde by minimizing solvent-mediated hydrogen atom transfer.^20^ Consistent with this interpretation, deuterium-labeling experiments indicated that the reduced byproduct arises from HAT with THF rather than proton transfer (see the Supporting Information for details). Further examination of the cyclization conditions showed that LiBr as an additive increased the reducing ability of SmI_2_, thus promoting productive ketyl radical formation from the sterically hindered ketone and intramolecular cyclization, rather than intermolecular aldehyde dimerization.^20^ A Parikh–Doering oxidation of the secondary alcohol provided **24** in 92% yield. Attempts to directly methylate **24** through alkylation of the corresponding enolate intermediate with iodomethane led to the incorrect configuration at C15 along with the dimethylation. To circumvent this, a sequential methylenation and in situ Pd/C hydrogenation was used to introduce the methyl group. Removal of the silyl protecting groups completed the first total synthesis of dasyscyphin A (**2**) in 13 steps.

In summary, we have developed a concise strategy for the rapid assembly of the core frameworks of habiterpenol (**1**) and dasyscyphin A (**2**) through sequential polyolefin cyclization and quaternary carbon center epimerization. By overriding the inherent stereochemical bias of polyolefin cyclization reactions, this approach enables access to polycyclic architectures bearing unconventional stereochemical patterns that would traditionally require multistep synthetic sequences. This work demonstrates how stereocenter remodeling can be leveraged to streamline the synthesis of complex molecules, while introducing a retrosynthetic paradigm in which bond construction and stereochemical installation are strategically decoupled.

## Supplementary Material



## Notes

Materials and methods, experimental procedures, optimization studies, NMR spectra, and other characterization data (PDF)
