# Supplementary material for: Overriding Stereochemical Outcomes in Cyclase Phase Total Synthesis: Enantioselective Synthesis of Habiterpenol and Dasyscyphin A
Source: J Am Chem Soc. 2026 Feb 11;148(7):6820–5. doi: 10.1021/jacs.6c00141 (PMC12951448; doi:10.1021/jacs.6c00141)
Supplement: Supplementary file 1 [file ja6c00141_si_001.pdf]

Supporting Information

Overriding Stereochemical Outcomes in Cyclase Phase Total Synthesis: Enantioselective Synthesis of Habiterpenol and Dasyscyphin A

Licheng Wu, Long H. Nguyen, Liwen Yan, Haoyu Yin, Natsuki Mizuno, and Alexander W. Schuppe*

Department of Chemistry, Vanderbilt University, Nashville, Tennessee, 37235, United States

Corresponding author: alexander.w.schuppe@vanderbilt.edu

Table of Contents

1. General experimental details 3
2. Selected previous syntheses of habiterpenol (**1**) 5
3. Synthesis toward habiterpenol (**1**) 7
   Synthesis of allyl bromide (**10**) 7
   Synthesis of alkyne (**11**) 7
   Synthesis of alkyne TMS (**12**) 8
   Synthesis of ketone (**4**) 9
   Synthesis of nitrile (**SI-2**) 10
   Synthesis of diol (**SI-3**) 11
   Synthesis of epoxide (**5**) 13
   Synthesis of alcohol (**14**) 14
   Synthesis of ketone (**4**) 15
   Synthesis of *epi*-ketone (**3**) 16
   Synthesis of triflate (**SI-5**) 18
   Synthesis of silyl enol ether (**15**) 18
   Synthesis of acetal (**16**) 20
   Synthesis of habiterpenol (**1**) 21
   Comparison of Natural and Synthetic **1** 22
4. Synthesis toward dasyscyphin A (**2**) 26
   Synthesis of nitrile (**19**) 26
   Synthesis of diol (**SI-6**) 26
   Synthesis of epoxide (**8**) 28
   Synthesis of alcohol (**20**) 29
   Synthesis of ketone (**7**) 30
   Synthesis of *epi*-ketone (**6**) 30
   Synthesis of alkene alcohol (**22**) 32
   Synthesis of OTES alkene (**SI-7**) 33
   Synthesis of aldehyde (**23**) 34
   Synthesis of diol (**SI-8**) 35
   Synthesis of ketone (**24**) 36
   Synthesis of dasyscyphin A (**2**) 37
   Comparison of natural and synthetic **2** 39
5. General procedure for the optimization of polyolefin cyclization 41
6. Additional attempts toward E-ring construction of habiterpenol 42
7. Additional attempts toward the D-ring construction of dasyscyphin A 43
8. Additional mechanistic probe for quaternary center epimerization 48
9. Computational details 51
10. References 60
11. NMR spectra 61

1. General experimental details

General Experimental Procedures: All reactions were performed in flame-dried or oven-dried (at 140 °C) glassware fitted with rubber or PTFE/silicone septa under a positive pressure of N_2, unless otherwise noted. Air- and moisture-sensitive liquids were transferred via syringe or stainless-steel cannula through rubber or PTFE/silicone septa. Unless otherwise noted, reactions were conducted in PYREX® reaction tube (catalog no. 99447). Solids were added under inert gas counter flow or were dissolved in the appropriate solvent. Reactions carried out at temperatures above room temperature were conducted in a preheated oil bath.

All reactions were magnetically stirred and monitored by ^{1}H NMR spectroscopy, Gas Chromatography/Mass Spectrometry (GC/MS), or analytical thin-layer chromatography (TLC), using glass-backed plates precoated with silica gel (250 μm, 60-Å pore size, Extra Hard Layer, SilicaPlate) impregnated with a fluorescent indicator (254 nm). TLC plates were visualized by exposure to ultraviolet light (UV), or were stained by submersion in iodine dispersed in SiO_2 (I_2), an acidic solution of *p*-anisaldehyde (PAA), an acidic solution of cerium ammonium molybdate (CAM), or an aqueous potassium permanganate solution ($KMnO_4$) and were developed by heating with a heat gun. Flash column chromatography was performed using Sorbtech® P60 silica gel (40–63 μm, 230–400 mesh, 60-Å pore diameter). The yields refer to chromatographically and spectroscopically (^{1}H and ^{13}C NMR) pure material. For light irradiation, Kessil PR160L-Blue LED lamps (max 30 W, λ_{max}= 390 nm) at 100% intensity were placed 10 cm away from the reaction flask.

Materials: Unless noted otherwise, all reagents and starting materials were purchased from commercial sources and used as received (Millipore Sigma, Thermo Fisher Scientific, Strem, TCI America, Combi-Blocks, Ambeed, Oakwood Chemical). $CDCl_3$ and CD_3OD was purchased from Milipore Sigma. Tetrahydrofuran (THF), acetonitrile (MeCN), toluene (PhMe), dimethylformamide (DMF), dichloromethane (CH_2Cl_2), and propionitrile (EtCN), *tert*-butanol (*t*-BuOH), and MeOH. Solvents for extraction, crystallization, and flash column chromatography were purchased in ACS grade from Fisher Scientific. K_2CO_3 and $K_3[Fe(CN)_6]$ were ground to a fine powder using a mortar and pestle prior to use.

Instrumentation: NMR spectra were measured on Bruker Avance III HD 400 or 600 MHz spectrometers and Bruker Ascend Evo 400 MHz spectrometer. Proton chemical shifts are expressed in parts per million (ppm, δ scale) and are referenced to the residual proton in the NMR solvent ($CDCl_3$: δ 7.26). ^{1}H NMR spectroscopic data are reported as follows: Chemical shift in ppm (multiplicity, coupling constants J (Hz), integration intensity, assigned proton). The multiplicities are abbreviated with s (singlet), br s (broad singlet), d (doublet), t (triplet), q (quartet), and m (multiplet). All ^{13}C spectra recorded are proton-decoupled. The carbon chemical shifts are expressed in parts per million (ppm, δ scale) and are referenced to the carbon resonance of the NMR solvent ($CDCl_3$: δ 77.2). ^{13}C NMR spectroscopic data are reported as follows: Chemical shift in ppm (multiplicity, coupling constants J (Hz), assigned carbon). All ^{19}F spectra were acquired without proton-decoupling. The ^{19}F chemical shifts are expressed in parts per million (ppm, δ scale). All raw ".fid" files were processed and analyzed using MestReNOVA 14.3 from Mestrelab Research S. L. High-resolution mass spectra were obtained on a LTQ Orbitrap XL™ Hybrid FT MassSpectrometer and an Agilent Technologies 6550 Q-TOF LC/MS system using an Agilent Zorbax 300

SB-C3 (2.1 × 150 mm, 5-μm particle size). Enantiomeric ratio (er) was determined either by chiral HPLC analysis using Agilent 1260 Infinity II Quaternary System equipped with 4.6 mm I.D x 250 mm columns containing chiral stationary phases; specific columns and analytic methods are provided in the experimental details for individual compounds; the wavelengths of light used for chiral analyses are provided with the associated chromatograms. Optical rotation data was obtained using a Rudolph Research Analytical AutoPol IV Automatic Polarimeter. Specific optical rotations were reported for $CHCl_3$ solutions using a wavelength of 589 nm unless otherwise noted. IR spectra were obtained on a Nicolet iS5 spectrometer equipped with an iD5 diamond laminate ATR accessory from Thermo Scientific. IR spectra were acquired from thin-film, neat samples. If required, substances were dissolved in CH_2Cl_2 prior to direct application on the ATR unit. Data are reported as follows: frequency of absorption (cm^{-1}).

2. Selected previous syntheses of habiterpenol (1)

Scheme SI-1. Nagamitsu's synthesis of habiterpenol in 2020

Org. Lett. **2020**, *22*, 5131

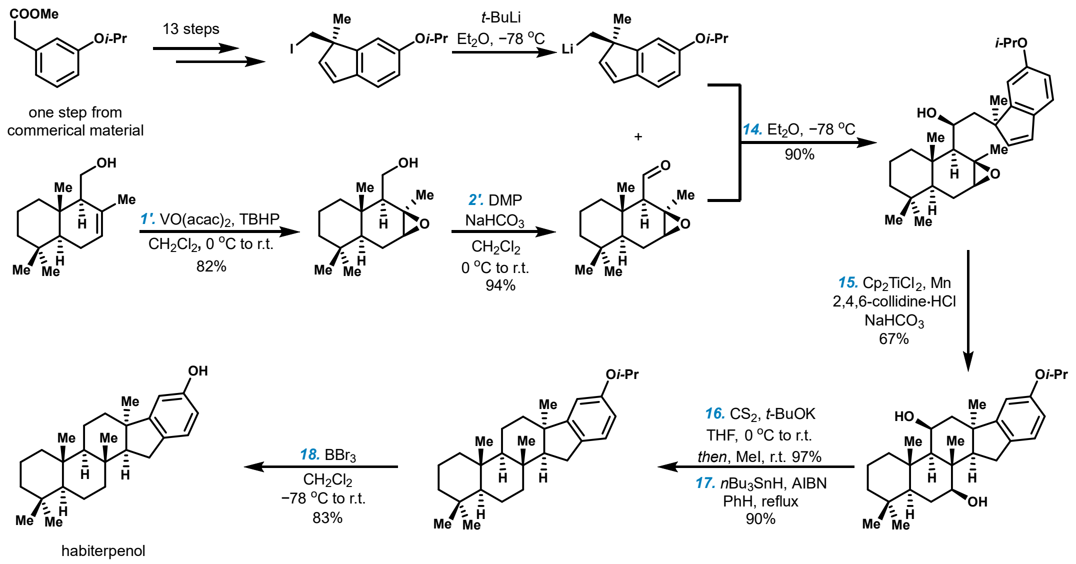

Scheme SI-2. Nagamitsu's synthesis of habiterpenol in 2023 using sclareolide

Org. Biomol. Chem. **2023**, *21*, 6129

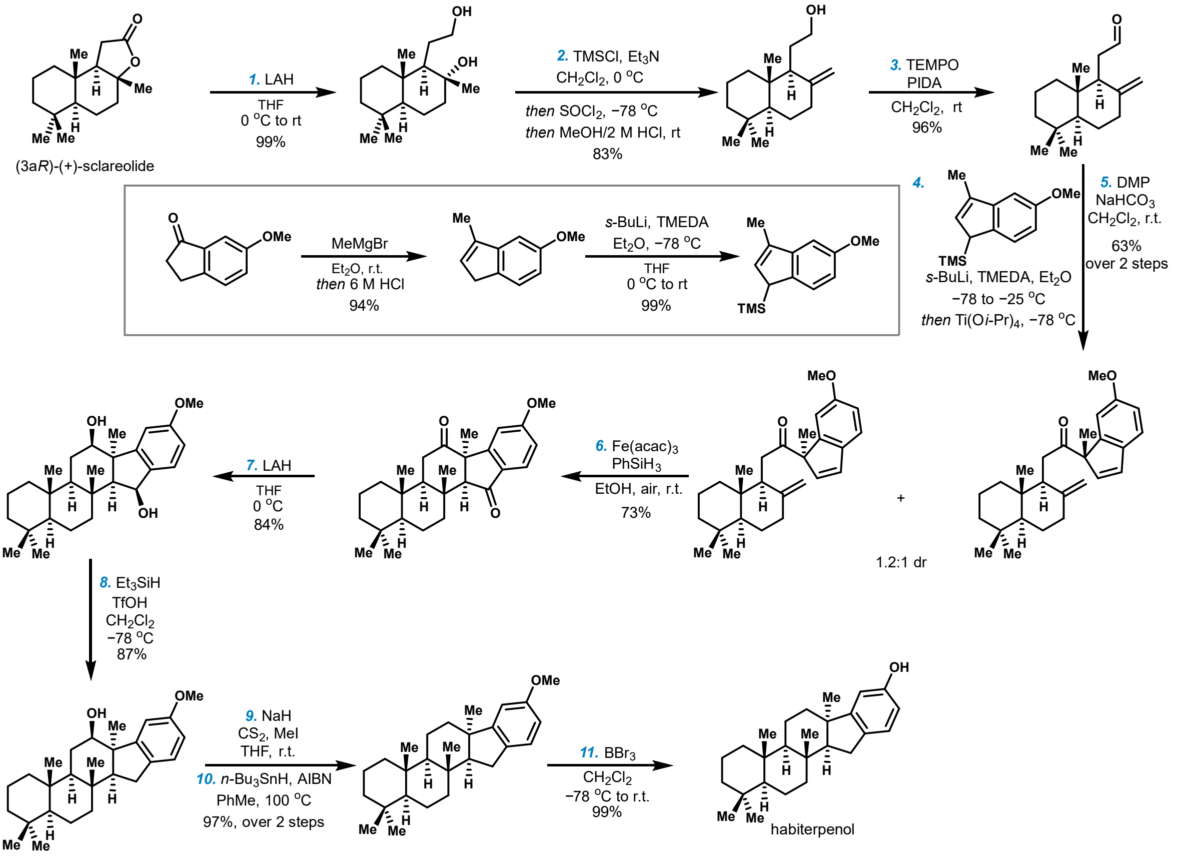

Scheme SI-3. Boonsombat's semi-synthesis of habiterpenol in 2025

Chem. Asian J. **2025**, *20*, e202500130

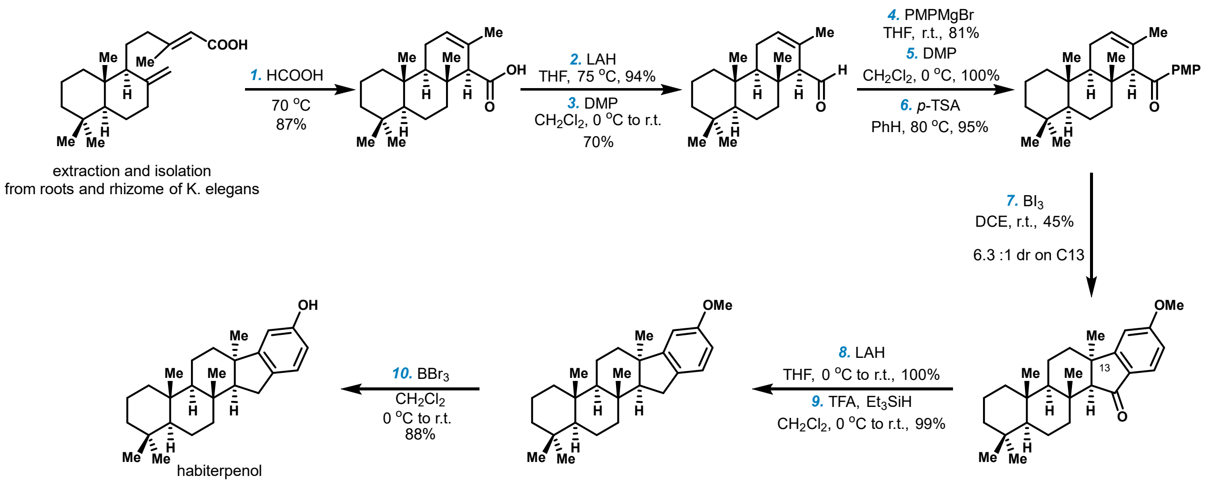

3. Synthesis toward habiterpenol (1)

Synthesis of allyl bromide (10)

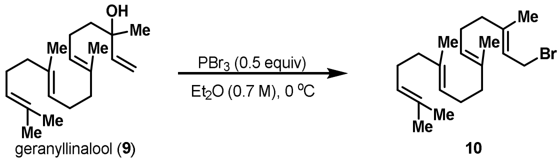

geranyllinalool (**9**) **10**

To a flame-dried 3 L round-bottom flask equipped with a magnetic stir-bar was charged with geranyllinalool (170 mL, 516 mmol, 1.0 equiv) and Et_2O (50 mL, 0.7 M). The reaction flask was placed in a 0 °C ice-water bath. To the stirred reaction mixture was added PBr_3 (25.6 mL, 258 mmol, 0.5 equiv) dropwise over 10 min at 0 °C. After 3 h, the reaction mixture was diluted with Et_2O (1.0 L) and H_2O (0.5 L), transferred to a separatory funnel, and the layers were separated. The aqueous layer was extracted with Et_2O (3 x 200 mL). The combined organic extracts were dried over anhydrous Na_2SO_4, filtered, and concentrated *in vacuo* with the aid of a rotary evaporator to yield **10** (181 g, 99%) as a colorless oil and 3:1 mixture of *E/Z* isomers. The crude allyl bromide was used in the subsequent step without further purification.

Synthesis of alkyne (11)

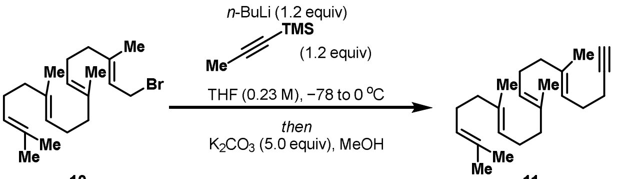

10 **11**

To a flame-dried 250 mL round-bottom flask equipped with a magnetic stir-bar was charged with trimethyl(prop-1-ynyl)silane (5.04 mL, 34.0 mmol, 1.2 equiv) and THF (40 mL, 0.85 M). The reaction vessel was placed in a −78 °C dry ice/acetone bath. To the stirred reaction mixture, *n*-butyllithium (13.6 mL, 34.0 mmol, 1.2 equiv, 2.5 M in hexanes) was added over 10 min. Then the reaction vessel was placed in a −40 °C dry ice/acetonitrile bath and allowed to stir for 15 min to provide a solution of the lithium reagent. A separate flame-dried 250 mL Schlenk flask was charged with **10** (10.0 g, 28.3 mmol, 1.0 equiv) and THF (82 mL, 0.35 M). The reaction vessel was placed in a −78 °C dry ice/acetone bath. To the stirred reaction mixture, the lithium reagent solution was added over 10 min via cannula. The reaction vessel was placed in a 0 °C ice-water bath and allowed to stir. After 1 h, to the stirred reaction mixture was added MeOH (85 mL) and K_2CO_3 (19.6 g). The reaction vessel was removed from the ice-water bath and stirred at room temperature. After 20 h, the reaction mixture was diluted with sat. aq. NH_4Cl (50 mL), transferred to a separatory funnel and the layers were separated. The aqueous layer was extracted with hexanes (3 x 50 mL). The combined organic extracts were washed with brine (50 mL), dried over anhydrous Na_2SO_4, filtered, and concentrated *in vacuo* with the aid of a rotary evaporator. The residue was purified by flash column chromatography on silica gel (2% EtOAc in hexanes) to yield **11** as a yellow oil and 3:1 mixture of *E/Z* isomers (3.9 g, 44%).

^{1}H NMR (600 MHz, $CDCl_3$, mixture of *E/Z* isomers): δ 5.18 (t, *J* = 7.2 Hz, 1H), 5.14–5.08 (m, 3H), 2.26–2.22 (m, 2H), 2.21–2.17 (m, 2H), 2.12–2.04 (m, 6H), 2.03–1.96 (m, 6H), 1.95–1.93 (m, 1H), 1.68 (s, 3H), 1.64–1.61 (m, 3H), 1.61–1.57 (m, 9H).

^{13}C NMR (151 MHz, $CDCl_3$, mixture of *E/Z* isomers): δ 137.1, 136.9, 135.5, 135.2, 135.1, 135.0, 131.4, 124.5, 124.5, 124.4, 124.3, 124.2, 124.1, 123.3, 122.6, 84.7, 84.6, 68.3, 68.2, 39.9, 39.8, 39.8, 32.2, 27.3, 27.2, 26.9, 26.8, 26.7, 26.7, 26.7, 25.8, 23.5, 19.3, 19.1, 17.8, 16.3, 16.2, 16.1.

IR (Diamond-ATR, neat) ṽ (cm^{-1}): 2960, 2909, 2852, 2166, 1248, 865.

HRMS (ESI): m/z: $[M+H]^+$ calc'd for $C_{23}H_{37}^+$: 313.2890. Found: 313.2886.

Synthesis of alkyne TMS (12)

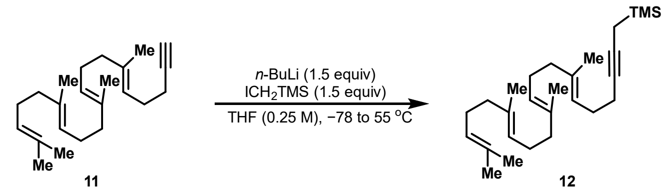

To a flame-dried 250 mL round-bottom flask equipped with a magnetic stir-bar was charged with **11** (3.52 g, 11.3 mmol, 1.0 equiv) and THF (45 mL, 0.25 M). The reaction vessel was placed in a −78 °C dry ice/acetone bath. To the stirred reaction mixture, *n*-butyllithium (6.76 mL, 16.9 mmol, 1.5 equiv, 2.5 M in hexanes) was added over 10 min. The reaction mixture was stirred at −78 °C. After 15 min, the reaction vessel was placed in 0 °C ice-water bath and allow to stir. After 15 min, to the stirred reaction mixture was added iodo(trimethylsilyl)methane (2.51 mL, 16.9 mmol, 1.5 equiv) dropwise over 2 min. The reaction flask was then covered with aluminium foil and placed in a preheated oil bath at 55 °C. After 12 h, the reaction was removed from the oil bath and allowed to cool to room temperature. Once at room temperature, the reaction mixture was diluted with H_2O (10 mL), transferred to a separatory funnel, and the layers were separated. The aqueous layer was extracted with EtOAc (2 x 30 mL). The combined organic extracts were washed with brine (50 mL), dried over anhydrous Na_2SO_4, filtered, and concentrated *in vacuo* with the aid of a rotary evaporator. The residue was purified by flash column chromatography on silica gel (1% EtOAc in hexanes) to yield **12** as a yellow oil and 3:1 mixture of *E/Z* isomers (3.2 g, 71%).

^{1}H NMR (600 MHz, $CDCl_3$, mixture of *E/Z* isomers): δ 5.21–5.17 (m, 1H), 5.14–5.09 (m, 3H), 2.18–2.14 (m, 4H), 2.11–2.03 (m, 7H), 2.01–1.95 (m, 5H), 1.68 (s, 3H), 1.62–1.58 (m, 12H), 1.43–1.40 (m, 2H), 0.09 (s, 9H).

^{13}C NMR (151 MHz, $CDCl_3$, mixture of *E/Z* isomers): δ 136.3, 136.1, 135.4, 135.1, 135.1, 135.0, 131.4, 124.5, 124.4, 124.4, 124.3, 124.2, 123.4, 78.9, 78.9, 39.9, 32.2, 28.3, 28.1, 26.9, 26.8, 26.8, 26.7, 26.7, 25.8, 23.6, 19.8, 19.6, 17.8, 16.3, 16.3, 16.1, 16.1, 7.1, −2.0.

IR (Diamond-ATR, neat) ṽ (cm^{-1}): 2963, 2914, 2853, 1438, 1379, 1103, 983, 831.

HRMS (ESI): m/z: $[M+H]^+$ calc'd for $C_{27}H_{47}Si^+$: 399.3442. Found: 399.3454.

Synthesis of ketone (4)

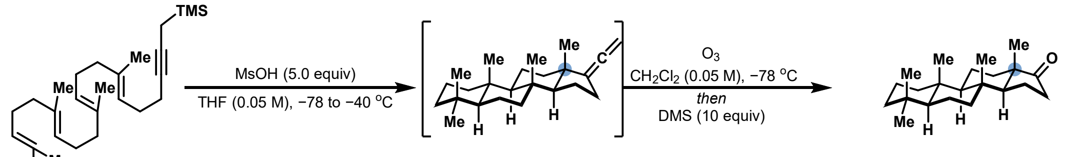

To an oven-dried reaction tube equipped with a magnetic stir-bar was added **12** (20 mg, 50 μmol, 1.0 equiv) and CH_2Cl_2 (1.0 mL, 0.05 M). The reaction vessel was placed in a −78 °C dry ice/acetone bath. To the stirred reaction mixture, methanesulfonic acid (16 μL, 0.25 mmol, 5.0 equiv) was added. The reaction was placed in −40 °C dry ice/acetonitrile bath and allowed to stir. After 1 h, the reaction mixture was diluted with sat. aq. $NaHCO_3$ (1 mL), transferred to a separatory funnel, and the layers were separated. The aqueous layer was extracted with CH_2Cl_2 (3 x 5 mL). The combined organic layers were washed with brine (10 mL), dried over anhydrous Na_2SO_4, filtered, and concentrated *in vacuo* with the aid of a rotary evaporator. The crude mixture was used without further purification.

To an oven-dried reaction tube equipped with a magnetic stir-bar was added above residue and CH_2Cl_2 (1.0 mL, 0.05 M). The reaction vessel was placed in a –78 °C dry ice/acetone bath. O_3 was bubbled through the reaction mixture until a blue colored solution was observed. After 10 min, N_2 was bubbled through the reaction solution for 10 min. To the resulting reaction mixture was added dimethyl sulfide (37 μL, 0.5 mmol, 10.0 equiv). The reaction vessel was removed from the dry ice/acetone bath and allowed to stir at room temperature. After 4 h, the reaction mixture was concentrated *in vacuo* with the aid of a rotary evaporator. The residue was purified by preparatory thin-layer chromatography on silica gel (10% EtOAc in hexanes) to yield **4** (*ca.* 0.1 mg).

^{1}H NMR (600 MHz, $CDCl_3$): δ 2.40 (ddd, *J* = 18.7, 8.8, 1.0 Hz, 1H), 2.04 (dt, *J* = 19.2, 8.7 Hz, 1H), 1.79–1.73 (m, 2H), 1.72–1.57 (m, 5H), 1.57–1.50 (m, 1H), 1.46–1.32 (m, 4H), 1.28 (dd, *J* = 12.7, 5.7 Hz, 1H), 1.21 (td, *J* = 12.9, 4.0 Hz, 1H), 1.11 (td, *J* = 13.3, 3.9 Hz, 1H), 1.04 (td, *J* = 13.0, 4.0 Hz, 1H), 0.95 (s, 3H), 0.93 (s, 3H), 0.84 (s, 3H), 0.83 (s, 3H), 0.80 (s, 3H), 0.80–0.77 (m, 2H).

^{13}C NMR (151 MHz, $CDCl_3$): 221.4, 61.6, 58.9, 57.3, 48.3, 42.3, 41.1, 40.2, 38.2, 37.6, 35.8, 33.5, 33.5, 21.5, 18.7, 18.1, 18.0, 17.7, 17.6, 16.9, 16.2.

IR (Diamond-ATR, neat) ṽ (cm^{-1}): 2934, 2852, 1728, 1044, 1011, 1002.

HRMS (ESI): m/z: $[M+H]^+$ calc'd for $C_{22}H_{37}O^+$: 317.2839. Found: 317.2838.

Synthesis of nitrile (SI-2)

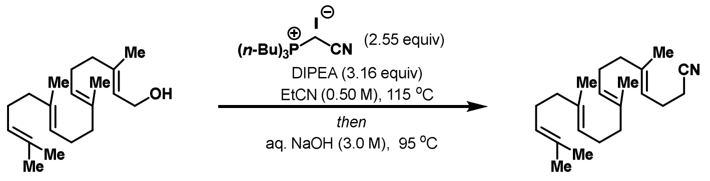

To an oven-dried 250 mL round-bottom flask equipped with a magnetic stir-bar was charged with geranylgeraniol (**13**) (14.8 g, 51.0 mmol, 1.0 equiv), and (cyanomethyl)tributylphosphonium iodide[1] (48.0 g, 130 mmol, 2.55 equiv). The flask was evacuated then backfilled with N_2 utilizing a dual-manifold Schlenk line. This process was repeated three times. The flask was fitted with an N_2-filled reflux condenser, then EtCN (102 mL, 0.50 M) was added. To the stirred reaction mixture was added *N*,*N*-diisopropylethylamine (28.0 mL, 161 mmol, 3.16 equiv) and reaction vessel was placed in a preheated oil bath at 95 °C. After 18 h, aq. NaOH (30 mL, 3.0 M) was added dropwise to the reaction mixture over 2 min at 95 °C. After 24 h, the reaction vessel was removed from the heating bath and allowed to cool to room temperature. Once at room temperature, aq. HCl (70 mL, 3.0 M) was added. The reaction mixture was transferred to a separatory funnel, and the layers were separated. The aqueous layer was extracted with EtOAc (3 x 80 mL). The combined organic extracts were washed with brine (100 mL), dried over anhydrous Na_2SO_4, filtered and concentrated *in vacuo* with the aid of a rotary evaporator. The crude reaction mixture was purified by flash column chromatography on silica gel (2% EtOAc in hexanes) to yield **SI-2** as a yellow oil (12.8 g, 80%).

^{1}H NMR (600 MHz, $CDCl_3$): δ 5.16–5.13 (m, 1H), 5.12–5.08 (m, 3H), 2.38–2.32 (m, 4H), 2.11–2.01 (m, 8H), 2.00–1.96 (m, 4H), 1.68 (s, 3H), 1.65 (s, 3H), 1.61–1.56 (m, 9H).

^{13}C NMR (151 MHz, $CDCl_3$): δ 139.3, 135.5, 135.1, 131.4, 124.5, 124.3, 123.9, 120.2, 119.8, 39.9, 39.8, 39.7, 26.9, 26.8, 26.6, 25.8, 24.2, 17.8, 17.8, 16.3, 16.2, 16.1.

IR (Diamond-ATR, neat) ṽ (cm^{-1}): 2963, 2914, 2853, 1438, 1379, 1103, 983, 831.

HRMS (ESI): m/z: $[M+H]^+$ calc'd for $C_{22}H_{36}N^+$: 314.2842. Found: 314.2837.

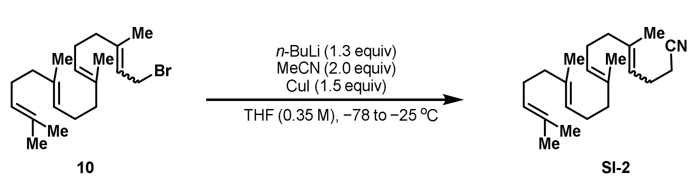

To a flame-dried 3 L three-neck round-bottom flask equipped with a magnetic stir-bar and a 250 mL addition funnel was added MeCN (40.2 mL, 0.77 mol, 2.0 equiv) and THF (1.09 L, 0.35 M). The reaction vessel was placed in a −78 °C dry ice/acetone bath. To the stirred reaction mixture, *n*-butyllithium (200.0 mL, 2.5 M solution in hexane, 500 mmol, 1.3 equiv) was added dropwise over 30 min at −78 °C via dropping funnel. After 40 min, the reaction vessel was transferred to a −10 °C ice-brine bath. Then CuI (110 g, 577 mmol, 1.5 equiv) was added to the reaction mixture. The reaction mixture was stirred at −10 °C. After 30 min, **10** (136 g, 385 mmol, 1.0 equiv) was added dropwise

over 30 min via cannula, and the reaction mixture was allowed to stir at −10 °C. After 1 h, the reaction mixture was removed from the cooling bath and allowed to gradually warm to room temperature. After stirring for 2 h at room temperature, the reaction mixture was diluted with sat. aq. NH_4Cl (800 mL), filtered through Celite (150 mL fritted funnel, 1 cm Celite powder), and eluted with EtOAc (3 x 100 mL). The filtrate was transferred to a separatory funnel, and the layers were separated. The organic layer was washed with brine (600 mL), dried over anhydrous Na_2SO_4, filtered, and concentrated *in vacuo* with the aid of a rotary evaporator. The crude mixture was diluted with hexanes (600 mL) and filtered through a pad of silica gel (150 mL fritted funnel, 4 cm silica gel), eluted with 5% EtOAc in hexanes (500 mL). The filtrate was concentrated *in vacuo* with the aid of a rotary evaporator to yield **SI-2** as a yellow oil and 3:1 mixture of *E/Z* isomers (98.6 g, 82%). The crude product was used without further purification.

Synthesis of diol (SI-3)

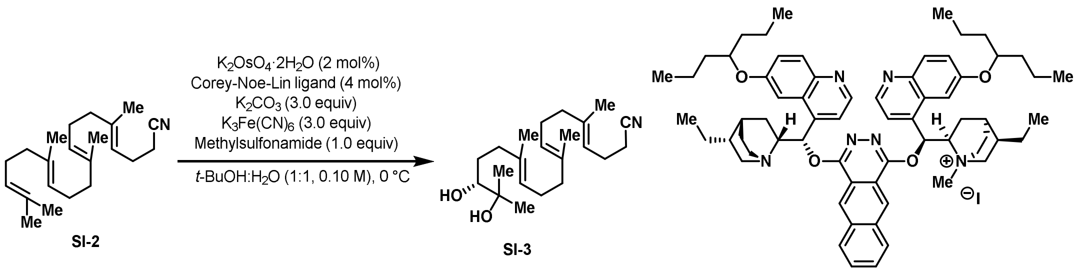

To an oven-dried 250 mL round-bottom flask equipped with a magnetic stir-bar was charged with $K_2OsO_4•2H_2O$ (81 mg, 219 μmol, 2.0 mol%), Corey-Noe-Lin Ligand[2] (500.0 mg, 439 μmol, 4.0 mol%), methylsulfonamide (971 mg, 10.2 mmol, 1.0 equiv), K_2CO_3 (4.23 g, 30.6 mmol, 3.0 equiv), $K_3Fe(CN)_6$ (10.1 g, 30.6 mmol, 3.0 equiv). Water and *t*-BuOH (102 mL, 0.1 M, 1:1 v/v) were added, and the reaction vessel was placed in a sonication bath until the reaction mixture was fully homogenous (*ca.* 10 min). The reaction vessel was removed from the sonication bath, transferred to a 0 °C ice-water bath with vigorous stirring, and **SI-2** (3.20 g, 10.2 mmol, 1.0 equiv) was added. The reaction vessel was sealed with a rubber septum and stirred at 0 °C. After 24 h, the reaction mixture was diluted sat. aq. $Na_2S_2O_3$ (15 mL) and sat. aq. Na_2SO_3 (15 mL) at 0 °C. The reaction vessel was removed from the ice-water bath and allowed to warm to room temperature, then NaOH (100 mL, 1.0 M) was added. The reaction mixture was transferred to a separatory funnel, and the layers were separated. The aqueous layer was extracted with EtOAc (3 x 300 mL). The combined organic extracts were washed with brine (150 mL), dried over anhydrous Na_2SO_4, filtered, and concentrated *in vacuo* with the aid of a rotary evaporator. The crude mixture was purified by flash column chromatography on silica gel (gradient elution: 20% to 50% EtOAc in hexanes) to yield **SI-3** as a pale-yellow oil (1.33 g, 38%). The *e.e.* of the product was determined by HPLC after derivatization with benzoyl anhydride: To a vial were added product **SI-3** (1.0 equiv), 4-dimethylaminopyridine (1.5 equiv), benzoyl anhydride (1.5 equiv) and CH_2Cl_2 (0.10 M). The reaction was stirred at room temperature for 2 h. After completion, the reaction mixture was purified by preparative thin-layer chromatography to afford the corresponding benzoylated compound. HPLC analysis was performed with a Chiralcel chiral OJ-H column. The enantiomeric excess of benzoate was determined by elution with 96:4 mixture of *n*-hexane and 2-propanol (1.0 mL/min). Retention times

for the enantiomers of benzoate were 12.93 min (minor) and 15.22 min (major), respectively; 95% *e.e.*

^{1}H NMR (600 MHz, $CDCl_3$): δ 5.17 (t, *J* = 6.9 Hz, 1H), 5.14 (t, *J* = 6.4 Hz, 1H), 5.09 (t, *J* = 6.6 Hz, 1H), 3.33 (d, *J* = 10.2 Hz, 1H), 2.37–2.28 (m, 5H), 2.26–2.14 (m, 2H), 2.11–2.03 (m, 5H), 2.03–1.96 (m, 4H),1.63 (s, 3H), 1.60 (s, 3H), 1.58 (s, 3H), 1.57–1.54 (m, 1H), 1.39 (dddd, *J* = 14.0, 10.5, 8.6, 5.5 Hz, 1H), 1.18 (s, 3H), 1.14 (s, 3H).

^{13}C NMR (151 MHz, $CDCl_3$): δ 139.2, 135.3, 135.0, 125.1, 124.1, 120.2, 119.8, 78.4, 73.1, 39.7, 39.7, 37.0, 29.8, 26.7, 26.5, 26.5, 24.1, 23.4, 17.8, 16.3, 16.1, 16.1.

IR (Diamond-ATR, neat) ṽ (cm^{-1}): 3430, 2920, 2857, 1666, 1443, 1383, 1160, 1074, 926.

HRMS (ESI): m/z: $[M+H]^+$ calc'd for $C_{22}H_{38}NO_2^+$: 348.2897. Found: 348.2886.

Specific Rotation $[\alpha]^{23}_D$: +23.7 (*c* = 1.2, $CHCl_3$).

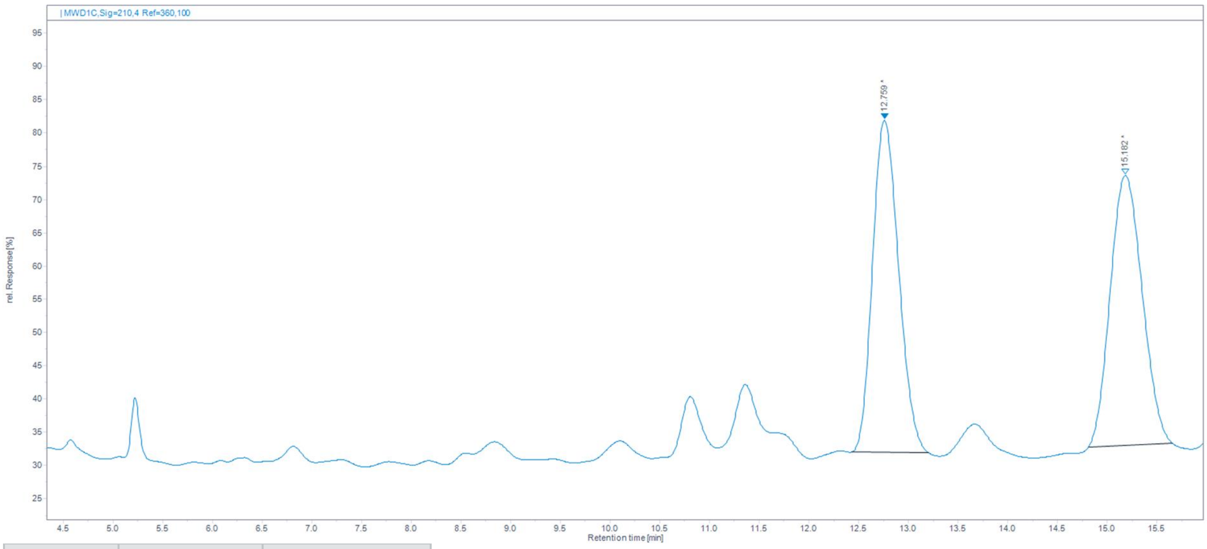

#	RT (min)	Area%
1	12.759	50.071
2	15.182	49.929

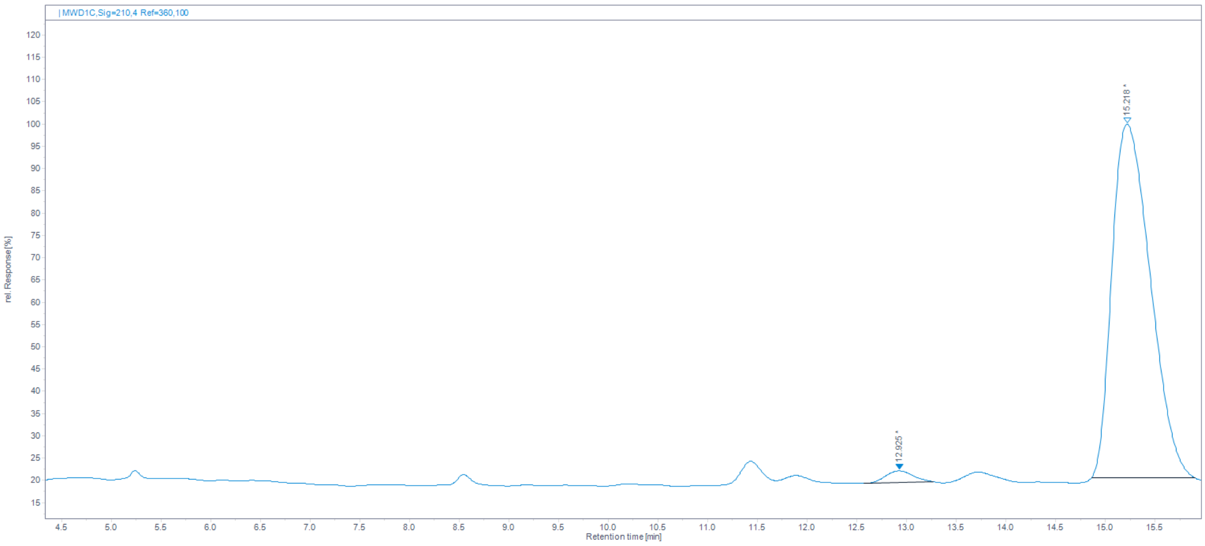

#	RT (min)	Area%
1	12.925	2.329
2	15.218	97.671

Synthesis of epoxide (5)

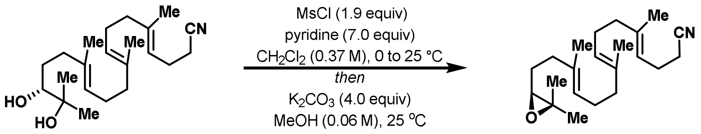

To a flame-dried 250 mL round-bottom flask equipped with a magnetic stir-bar was charged with **SI-3** (1.29 g, 3.7 mmol, 1.0 equiv). The reaction vessel was sealed with a rubber septum, evacuated, and then backfilled with N_2 utilizing a dual manifold Schlenk line. This process was repeated three times. To the reaction vessel, CH_2Cl_2 (10.5 mL, 0.37 M) and pyridine (2.0 mL, 25.9 mmol, 7.0 equiv) were added. The reaction mixture placed in a 0 °C ice-water bath, and methanesulfonyl chloride (458 μL, 5.9 mmol, 1.6 equiv) was added dropwise over 5 min. The flask was removed from the cooling bath and allowed to stir at room temperature for 4.5 h, then a second portion of methanesulfonyl chloride (86 μL, 1.1 mmol, 0.3 equiv) was added dropwise over 2 min. After an additional 3.5 h, MeOH (51 mL) and K_2CO_3 (2.05 g, 14.8 mmol, 4.0 equiv) were added sequentially to the reaction mixture. The reaction mixture was stirred at room temperature. After 24 h, the crude reaction mixture was concentrated *in vacuo* with the aid of a rotary evaporator. The reaction mixture was then diluted with water (50 mL) and Et_2O (50 mL), transferred to a separatory funnel, and the layers were separated. The aqueous layer was extracted with Et_2O (3 x 50 mL). The combined organic extracts were washed with brine (100 mL), dried over anhydrous Na_2SO_4, filtered, and concentrated *in vacuo* with the aid of a rotary evaporator. The crude mixture was purified by flash column chromatography on silica gel (gradient elution: 5% to 25% EtOAc in hexanes) to yield **5** as a pale-yellow oil (1.00 g, 82%).

^{1}H NMR (600 MHz, $CDCl_3$): δ 5.17–5.13 (m, 2H), 5.11–5.08 (m, 1H), 2.70 (t, J = 6.2 Hz, 1H), 2.37–2.32 (m, 4H), 2.15 (ddd, J = 14.6, 8.9, 6.9 Hz, 1H), 2.11–2.05 (m, 5H), 2.04–1.96 (m, 4H), 1.65 (s, 3H), 1.62–1.58 (m, 1H), 1.61 (s, 3H), 1.60 (s, 3H), 1.57–1.56 (m, 1H), 1.30 (s, 3H), 1.26 (s, 3H).

^{13}**C NMR** (151 MHz, $CDCl_3$): δ 139.3, 135.4, 134.2, 125.0, 124.0, 120.2, 119.8, 64.3, 58.5, 39.8, 39.7, 36.5, 27.6, 26.8, 26.6, 25.1, 24.1, 18.9, 17.8, 16.3, 16.2, 16.1.

IR (Diamond-ATR, neat) ṽ (cm^{-1}): 2961, 2912, 2851, 2365, 2333, 1440, 1432, 1379, 1322, 1248, 1121, 876.

HRMS (ESI): m/z: $[M+H]^+$ calc'd for $C_{22}H_{36}NO^+$: 330.2785. Found: 330.2791.

Specific Rotation $[\alpha]^{23}_D$: −2.8 (*c* = 1.0, $CHCl_3$).

Synthesis of alcohol (14)

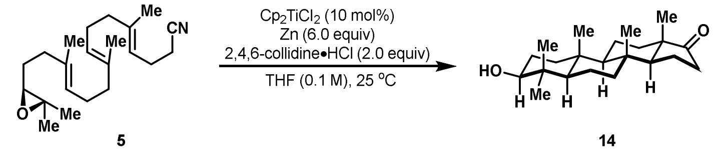

Activation of zinc: To a 500 mL round-bottom flask equipped with a magnetic stir-bar was charged with zinc dust (10.0 g, 150.0 mmol, 1.0 equiv) and aq. HCl (30.0 mL, 1.0 M). The reaction mixture was stirred at room temperature for 30 min, then filtered through a Buchner funnel fitted with two layers of filter paper. The solid was washed with H_2O (50 mL), acetone (50 mL), methanol (50 mL), and hexanes (50 mL). The resulting solid was collected into a 500 mL round-bottom flask equipped with a magnetic stir-bar and was allowed to stir under high vacuum utilizing a dual-manifold Schlenk line for 48 h to yield activated zinc.

To a flame-dried 1 L round-bottom flask equipped with a magnetic stir-bar was charged with Cp_2TiCl_2 (1.04 g, 4.16 mmol, 10 mol%), activated zinc (16.3 g, 249 mmol, 6.0 equiv), and 2,4,6-collidine hydrochloride (13.1 g, 83.1 mmol, 2.0 equiv). The reaction flask was transferred to a N_2-filled glovebox, and THF (416 mL, 0.10 M) was added. The solution was allowed to stir for 10 min, resulting in a green heterogeneous mixture. To the reaction mixture was added **5** (13.7 g, 41.6 mmol, 1.0 equiv) dropwise over 3 min. The reaction vessel was sealed with a rubber septum, removed from the glovebox, and stirred at room temperature.

After 18 h, the reaction mixture was diluted with aq. KH_2PO_4 solution (10% w/v, 400 mL), and the resulting solution was stirred at room temperature. After 30 min, the reaction mixture was filtered over a packed pad of Celite (150 mL fritted funnel, 18 mm Celite powder) and washed with EtOAc (300 mL). The filtrate was transferred to a separatory funnel and the layers were separated. The aqueous layer was extracted with EtOAc (3 x 300 mL). The combined organic extracts were washed with sat. aq. HCl (300 mL, 1.0 M), brine (300 mL), dried over anhydrous Na_2SO_4, filtered, and concentrated *in vacuo* with the aid of a rotary evaporator. The residue was purified by flash column chromatography on silica gel (20% EtOAc and 20% CH_2Cl_2 in hexanes) to yield **14** as a colorless solid (4.2 g, 30%).

^{1}**H NMR** (600 MHz, $CDCl_3$): δ 3.17 (dd, *J* = 11.6, 4.7 Hz, 1H), 2.41 (ddd, *J* = 19.3, 8.9, 0.9 Hz, 1H), 2.05 (dt, *J* = 19.2, 8.9 Hz, 1H), 1.79–1.74 (m, 2H), 1.72–1.54 (m, 7H), 1.47 (dddd, *J* = 13.0, 13.0, 13.0, 2.6 Hz, 1H), 1.44–1.36 (m, 2H), 1.27 (dd, *J* = 13.0, 5.8 Hz, 1H), 1.21 (td, *J* = 13.1, 4.0 Hz, 1H), 1.04 (td, *J* = 12.9, 4.0 Hz, 1H), 0.99–0.96 (m, 1H), 0.95 (s, 6H), 0.93 (s, 3H), 0.84 (s, 3H), 0.77 (s, 3H), 0.76 (ddd, *J* = 19.1, 12.6,

2.1 Hz, 2H).

^{13}C NMR (151 MHz, $CDCl_3$): δ 221.3, 78.9, 61.4, 58.7, 56.1, 48.3, 41.0, 39.1, 38.6, 38.0, 37.3, 35.8, 33.4, 28.1, 27.4, 18.2, 17.7, 17.6, 17.6, 16.9, 16.2, 15.4.

IR (Diamond-ATR, neat) ṽ (cm^{-1}): 3328, 2965, 2927, 2866, 2848, 1072, 1051, 1018, 995, 974.

HRMS (ESI): m/z: $[M+H]^+$ calc'd for $C_{22}H_{37}O_2^+$: 333.2788. Found: 333.2792.

Specific Rotation $[\alpha]^{23}_D$: +57.4 (*c* = 1.0, $CHCl_3$).

Synthesis of ketone (4)

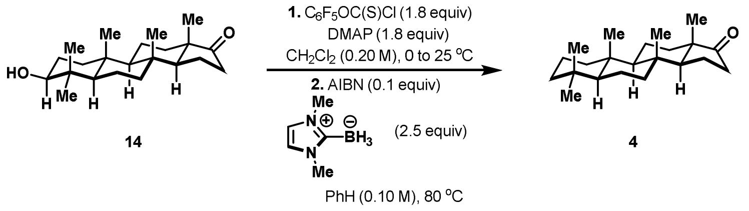

To a flame-dried reaction tube equipped with a magnetic stir-bar was charged with **14** (130 mg, 375 µmol, 1.0 equiv), 4-dimethylaminopyridine (83 mg, 675 µmol, 1.8 equiv), and CH_2Cl_2 (1.9 mL, 0.20 M). The reaction vessel was placed in a 0 °C ice-water bath. To the stirred reaction mixture, *O*-(perfluorophenyl) carbonochloridothioate (177 mg, 675 µmol, 1.8 equiv) was added. The reaction vessel was removed from the ice-water bath and allowed to stir at room temperature. After 14 h, the reaction mixture was diluted with EtOAc (20 mL) and sat. aq. $NaHCO_3$ (10 mL). The resulting mixture was transferred to a separatory funnel and the layers were separated. The aqueous layer was extracted with EtOAc (2 x 10 mL). The combined organic extracts were washed with brine (30 mL), dried over anhydrous Na_2SO_4, filtered, and concentrated *in vacuo* with the aid of a rotary evaporator. The crude product was used in the subsequent step without further purification.

To a flame-dried reaction tube equipped with a magnetic stir-bar was charged with above thiocarbonate, 2,2'-azobis(2-methylpropionitrile) (6 mg, 38 µmol, 0.1 equiv), and 1,3-dimethyl-1*H*-imidazol-3-ium-2-yl)trihydroborate (103 mg, 938 µmol, 2.5 equiv). The reaction vessel was sealed with a screw cap (phenolic top with a polyvinyl-faced pulp liner), evacuated and then backfilled with N_2 utilizing a dual manifold Schlenk line. This process was repeated three times. The tube was charged with PhH (3.75 mL, 0.10 M) and placed in a preheated oil bath at 80 °C. After 4 h, the reaction vessel was removed from the oil bath and allowed to cool to room temperature. Once at room temperature, the reaction mixture was concentrated *in vacuo* with the aid of a rotary evaporator. The residue was purified by flash column chromatography on silica gel (2% EtOAc in hexanes) to yield **4** as a colorless solid (54 mg, 48% over two steps).

^{1}H NMR (600 MHz, $CDCl_3$): δ 2.40 (ddd, *J* = 18.7, 8.8, 1.0 Hz, 1H), 2.04 (dt, *J* = 19.2, 8.7 Hz, 1H), 1.79–1.73 (m, 2H), 1.72–1.57 (m, 5H), 1.57–1.50 (m, 1H), 1.46–1.32 (m, 4H), 1.28 (dd, *J* = 12.7, 5.7 Hz, 1H), 1.21 (td, *J* = 12.9, 4.0 Hz, 1H), 1.11 (td, *J* = 13.3, 3.9 Hz, 1H), 1.04 (td, *J* = 13.0, 4.0 Hz, 1H), 0.95 (s, 3H), 0.93 (s, 3H), 0.84 (s, 3H), 0.83 (s, 3H), 0.80 (s, 3H), 0.80–0.77 (m, 2H).

^{13}C NMR (151 MHz, $CDCl_3$): 221.4, 61.6, 58.9, 57.3, 48.3, 42.3, 41.1, 40.2, 38.2, 37.6, 35.8, 33.5, 33.5, 21.5, 18.7, 18.1, 18.0, 17.7, 17.6, 16.9, 16.2.

IR (Diamond-ATR, neat) $\tilde{\nu}$ (cm^{-1}): 2934, 2852, 1728, 1044, 1011, 1002.

HRMS (ESI): m/z: $[M+H]^+$ calc'd for $C_{22}H_{37}O^+$: 317.2839. Found: 317.2838.

Specific Rotation $[\alpha]^{23}_D$: +74.2 (*c* = 1.0, $CHCl_3$).

Synthesis of *epi*-ketone (3)

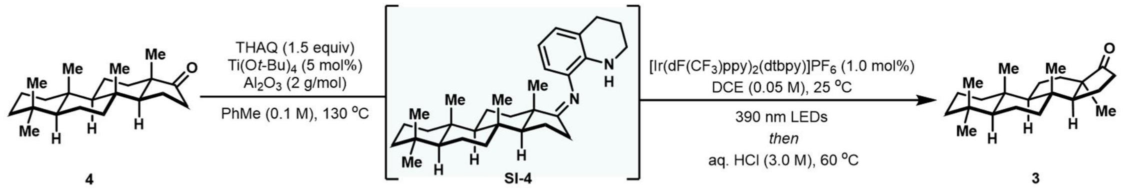

Inside a N_2-filled glovebox, a flame-dried reaction tube equipped with a magnetic stir-bar was charged with with **4** (54 mg, 0.17 mmol, 1.0 equiv), Al_2O_3 (340 mg, 2.0 g/mmol), and PhMe (1.7 mL, 0.10 M). To the stirred reaction mixture was added 1,2,3,4-tetrahydroquinolin-8-amine (38 mg, 0.26 mmol, 1.5 equiv) and Ti(O*t*-Bu)$_4$ (3 µL, 8.5 µmol, 5.0 mol%). The reaction vessel was sealed with a rubber septum, removed from the glovebox. The reaction mixture was placed in a preheated oil bath at 130 °C. After 18 h, the reaction mixture was removed from the oil bath and allowed to cool to room temperature. Once at room temperature, the reaction mixture was filtered over a short plug of SiO_2 (15 mL fritted funnel, 3 cm SiO_2, treated with 5% Et_3N in hexanes) and the reaction vessel was rinsed with hexanes (3 x 3 mL). The SiO_2 plug was eluted with 25% EtOAc in hexanes (30 mL) and the filtrate was concentrated *in vacuo* with the aid of a rotary evaporator. The crude mixture was successively azeotrope with benzene (15 mL) and CH_2Cl_2 (3 x 15 mL), then dried under high vacuum utilizing a dual-manifold Schlenk line to yield **SI-4** as a pale-yellow solid, which was used without further purification.

Note: The PhMe and Et_3N must be completely removed from the imine intermediate before proceeding to the subsequent operation.

Inside a N_2-filled glovebox, a flame-dried reaction tube equipped with a magnetic stir-bar was charged with [Ir(dF(CF_3)ppy)$_2$(dtbpy)]PF_6 (1.9 mg, 1.7 µmol, 1.0 mol%) and 1,2-dichloroethane (3.4 mL, 0.05 M). The reaction flask was sealed with a rubber septum and removed from the glovebox. The reaction vessel was subjected to LED irradiation utilizing one 30 W Kessil PR-160L 390 nm LEDs at 100% intensity at distances of 10 cm. After 4h, the reaction mixture was diluted with aq. HCl (3.4 mL, 3.0 M) and placed in a preheated oil bath at 60 ºC. After 4 h, the reaction mixture was removed from the oil bath and allowed to cool to room temperature. Once at room temperature, the reaction mixture was transferred to a separatory funnel and the layers were separated. The aqueous layer was extracted with CH_2Cl_2 (3 x 5 mL). The combined organic extracts were washed with brine (5 mL), dried over anhydrous Na_2SO_4, filtered, and concentrated *in vacuo* with the aid of a rotary evaporator. The residue was purified by flash column chromatography on silica gel (gradient elution: hexanes to 2% EtOAc in hexanes) to yield **3** as a colorless solid (32 mg, 60%)

^{1}H NMR (600 MHz, $CDCl_3$): δ 2.35 (ddd, J = 19.7, 10.3, 2.9 Hz, 1H), 2.26 (ddd, J = 13.1, 4.3, 2.0 Hz, 1H), 2.06 (dt, J = 18.7, 9.1 Hz, 1H), 2.03–1.96 (m, 1H), 1.88 (dt, 12.9, 3.4 Hz, 1H), 1.81–1.76 (m, 1H), 1.70 (dt, J = 12.7, 3.4 Hz, 1H), 1.63–1.57 (m, 1H), 1.57–1.55 (m, 1H), 1.50 (dtd, J = 13.5, 3.5, 1.9 Hz, 1H), 1.41–1.33 (m, 3H), 1.27 (dddd, J = 13.2, 13.2, 13.2, 3.3 Hz, 1H), 1.18–1.11 (m, 2H), 1.10–0.99 (m, 2H), 0.97 (s, 3H), 0.84 (s, 3H), 0.78 (s, 3H), 0.77 (s, 3H), 0.80–0.73 (m, 2H), 0.71 (dd, J = 11.9, 2.0 Hz, 1H), 0.64 (s, 3H).

^{13}C NMR (151 MHz, $CDCl_3$): δ 221.8, 58.8, 57.9, 56.8, 48.8, 42.6, 42.2, 40.2, 37.6, 37.5, 34.5, 33.5, 33.5, 31.7, 28.4, 21.6, 18.7, 18.3, 18.3, 18.2, 16.7, 16.1.

IR (Diamond-ATR, neat) ṽ (cm^{-1}): 2937, 2893, 2839, 1720, 1459, 1390, 1126, 1084.

HRMS (ESI): m/z: $[M+H]^+$ calc'd for $C_{22}H_{37}O^+$: 317.2839. Found: 317.2282.

Specific Rotation $[\alpha]^{23}_{D}$: −8.5 (c = 1.0, $CHCl_3$).

Scheme SI-4. Reaction mechanism of this quaternary center epimerization

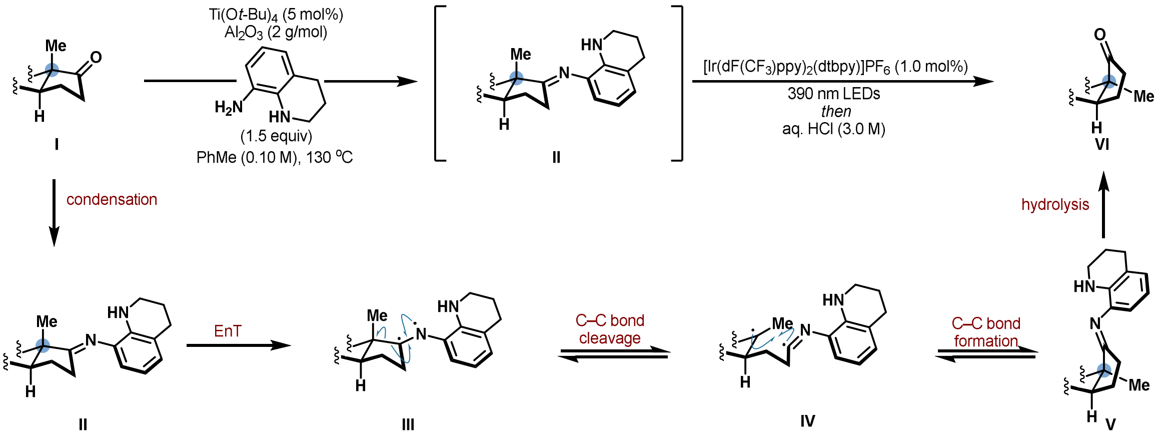

Mechanistically, the ketone was first condensed with THAQ using a Ti(IV) Lewis acid catalyst, to form the imine, which generates a triplet diradical upon energy transfer with an excited photocatalyst. Subsequent β-scission, radical center inversion, and C–C bond reformation could produce the epimerized ketone following facile hydrolysis.

Synthesis of triflate (SI-5)

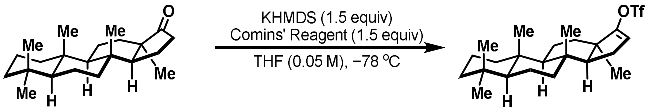

3 SI-5

To a flame-dried 25 mL round-bottom flask equipped with a magnetic stir-bar was added **3** (106 mg, 335 µmol, 1.0 equiv). The reaction vessel was sealed with a rubber septum, evacuated, and then backfilled with N_2 utilizing a dual manifold Schlenk line. This process was repeated three times. THF (3.5 mL, 0.10 M) was added, and the reaction vessel was placed in a −78 °C dry ice/acetone bath. To the stirred reaction mixture, KHMDS (0.5 mL, 502 µmol, 1.5 equiv, 1.0 M in PhMe) was added dropwise over 5 min. After 1 h, Comins' reagent (197 mg, 502 µmol, 1.5 equiv) in THF (3.5 mL, 0.14 M) was added dropwise over 5 min (final concentration: 0.05 M) at −78 °C. After 3 h, the reaction mixture was diluted with sat. aq. NH_4Cl (15 mL), transferred to a separatory funnel, and the layers were separated. The aqueous layer was extracted with Et_2O (3 x 10 mL). The combined organic extracts were washed with brine (10 mL), dried over anhydrous Na_2SO_4, filtered, and concentrated *in vacuo* with the aid of a rotary evaporator. The residue was purified by flash column chromatography on silica gel (hexanes) to yield **SI-5** as a colorless solid (143 mg, 95%).

^{1}H NMR (600 MHz, $CDCl_3$): δ 5.48 (t, J = 2.6 Hz, 1H), 2.44 (ddd, J = 17.1, 9.1, 2.1 Hz, 1H), 2.18 (dt, J = 17.1, 2.7 Hz, 1H), 1.79–1.71 (m, 1H), 1.67 (dt, J = 12.9, 3.1 Hz, 1H), 1.62–1.55 (m, 4H), 1.48 (dtd, J = 13.8, 3.4, 2.0 Hz, 1H), 1.46–1.35 (m, 4H), 1.32 (dddd, J = 13.2, 13.2, 13.2, 3.4 Hz, 1H), 1.17–1.10 (m, 1H), 1.14 (s, 3H), 0.98–0.92 (m, 2H), 0.89 (s, 3H), 0.85 (s, 3H), 0.84–0.79 (m, 2H), 0.82 (s, 3H), 0.81 (s, 3H).

^{13}C NMR (151 MHz, $CDCl_3$): δ 155.6, 118.7 (q, $J_{C\text{–}F}$ = 320.1 Hz), 112.1, 58.3, 57.0, 54.0, 45.0, 43.4, 42.2, 39.5, 38.0, 36.8, 33.6, 33.3, 28.9, 28.1, 27.4, 21.8, 18.5, 18.3, 17.4, 16.9, 15.4.

^{19}F NMR (471 MHz, $CDCl_3$): δ −73.8.

IR (Diamond-ATR, neat) ṽ (cm^{-1}): 2930, 2852, 1458, 1421, 1388, 1247, 1208, 1065, 1101, 1047, 970, 939, 900, 857, 848, 824.

HRMS (ESI): m/z: [M+H]$^+$ calc'd for $C_{23}H_{36}F_3O_3S^+$: 449.2332. Found: 449.2331.

Specific Rotation $[\alpha]^{23}_D$: +8.5 (c = 0.2, $CHCl_3$).

Synthesis of silyl enol ether (15)

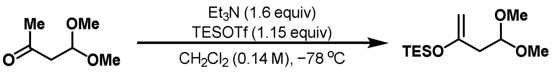

15

To a flame-dried 250 mL round-bottom flask equipped with a magnetic stir-bar was charged with 4,4-dimethoxybutan-2-one (1.0 mL, 7.53 mmol, 1.0 equiv), CH_2Cl_2 (54 mL, 0.14 M), and Et_3N (1.68 mL, 12.0 mmol, 1.6 equiv). The reaction vessel was placed in a −78 °C dry ice/acetone bath. To the stirred reaction mixture, triethylsilyl trifluoromethanesulfonate (1.96 mL, 8.66 mmol, 1.15 equiv) was added dropwise over

20 min. The reaction mixture was stirred at −78 °C. After 4 h, the reaction mixture was diluted with sat. aq. $NaHCO_3$ (20 mL), removed from the dry ice/acetone bath, and allowed to warm to room temperature. One at room temperature, the resulting mixture was transferred to a separatory funnel, and the layers were separated. The aqueous layer was extracted with CH_2Cl_2 (3 x 10 mL). The combined organic extracts were washed with brine (30 mL), dried over anhydrous Na_2SO_4, filtered, and concentrated *in vacuo* with the aid of a rotary evaporator. The residue was purified by flash column chromatography on neutral aluminum oxide (1% Et_2O in hexanes) to yield **15** as a pale-yellow liquid (696 mg, 38%)

^{1}H NMR (400 MHz, $CDCl_3$): δ 4.64 (t, *J* = 5.8 Hz, 1H), 4.11 (d, *J* = 1.1 Hz, 1H), 4.10–4.08 (m, 1H), 3.33 (s, 6H), 2.36 (dd, *J* = 5.8, 0.5 Hz, 2H), 0.98 (t, *J* = 7.9 Hz, 9H), 0.70 (q, *J* = 7.9 Hz, 6H).

^{13}C NMR (101 MHz, $CDCl_3$): δ 155.2, 102.2, 91.5, 52.9, 40.5, 6.8, 5.0.

IR (Diamond-ATR, neat) ṽ (cm^{-1}): 2953, 2912, 2876, 2829, 1624, 1458, 1416, 1359, 1310, 1262, 1236, 1184, 1121, 1061, 1001, 906, 812.

MS (EI): m/z: $[M-OMe]^+$ calc'd for $C_{11}H_{23}O_2Si^+$: 215.1. Found: 215.1.

Synthesis of acetal (16)

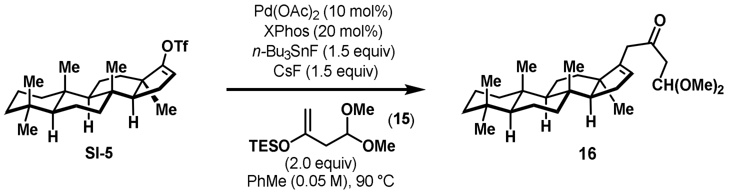

To a flame-dried reaction tube equipped with a magnetic stir-bar was charged with **SI-5** (7.2 mg, 16 µmol, 1.0 equiv), $Pd(OAc)_2$ (0.36 mg, 1.6 µmol, 10 mol%), XPhos (1.5 mg, 3.2 µmol, 20 mol%), and *n*-Bu_3SnF (7.4 mg, 24 µmol, 1.5 equiv). The reaction vessel was brought inside a N_2-filled glovebox. To the vial was added CsF (3.7 mg, 24 µmol, 1.5 equiv), PhMe (0.32 mL, 0.05 M), and silyl enol ether **15** (7.9 mg, 32 µmol, 2.0 equiv). The reaction vial was capped with a screw cap (phenolic top with a polyvinyl-faced pulp liner) and brought outside the glovebox. The reaction vial was placed in a preheated oil bath at 90 °C. After 2 h, the reaction vessel was removed from the oil bath and allowed to cool to room temperature. Once at room temperature, the reaction mixture was diluted with sat. aq. $NaHCO_3$ (2 mL), transferred to a separatory funnel, and the layers were separated. The aqueous layer was extracted with CH_2Cl_2 (3 x 2 mL). The combined organic extracts were washed with brine (2 mL), dried over anhydrous Na_2SO_4, filtered, and concentrated *in vacuo* with the aid of a rotary evaporator. The residue was purified by flash column chromatography on silica gel (gradient elution: hexanes to 10% EtOAc in hexanes) to yield **16** as a colorless solid (6.0 mg, 87%)

^{1}H NMR (600 MHz, $CDCl_3$): δ 5.31 (t, *J* = 2.6 Hz, 1H), 4.78 (t, *J* = 5.6 Hz, 1H), 3.35 (s, 6H), 3.00 (s, 2H), 2.77 (d, *J* = 5.7 Hz, 2H), 2.41–2.34 (m, 1H), 2.10 (dt, *J* = 17.2, 2.6 Hz, 1H), 1.66 (dt, *J* = 13.0, 3.0 Hz, 2H), 1.62–1.57 (m, 3H), 1.56–1.49 (m, 3H), 1.47–1.42 (m, 1H), 1.39–1.33 (m, 3H), 1.33–1.27 (m, 3H), 1.12 (td, *J* = 13.3, 4.8 Hz, 2H), 0.95 (s, 3H), 0.84 (s, 3H), 0.84 (s, 3H), 0.80 (s, 3H), 0.72 (s, 3H).

^{13}C NMR (151 MHz, $CDCl_3$): δ 206.4, 144.2, 126.4, 102.1, 61.1, 57.0, 55.2, 54.2, 54.1, 48.5, 45.6, 43.7, 43.0, 42.3, 39.7, 37.9, 37.1, 33.6, 33.3, 31.6, 31.4, 29.5, 21.8, 18.6, 18.4, 18.2, 17.1, 15.7.

IR (Diamond-ATR, neat) ṽ (cm^{-1}): 2926, 2849, 2359, 1716, 1458, 1386, 1364, 1299, 1191, 1115, 1057, 1009, 941, 900, 815.

HRMS (ESI): m/z: $[M+H]^+$ calc'd for $C_{28}H_{47}O_3^+$: 431.3520. Found: 431.3514.

Specific Rotation $[\alpha]^{23}_D$: +3.3 (*c* = 0.5, $CHCl_3$).

Synthesis of habiterpenol (1)

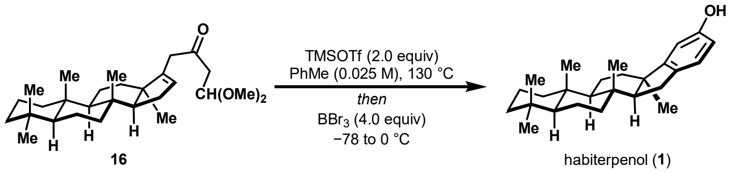

To a flame-dried reaction tube equipped with a stir-bar was charged with **16** (8.0 mg, 19 μmol, 1.0 equiv). The reaction vessel was sealed with a screw cap (phenolic top with a polyvinyl-faced pulp liner), evacuated and then backfilled with N_2 utilizing a dual manifold Schlenk line. This process was repeated three times. The tube was charged with PhMe (0.75 mL, 0.025 M) and placed in a 0 °C ice-water bath. To the stirred reaction mixture, trimethylsilyl trifluoromethanesulfonate (6.9 μL, 37 μmol, 2.0 equiv) was added, and the vessel was transferred to a preheated oil bath at 130 °C and allowed to stir. After 45 min, the reaction vessel was removed from the oil bath and allowed to cool to room temperature. Once at room temperature, the vessel was transferred to a −78 °C dry ice/acetone bath, and BBr_3 (74 μL, 74 μmol, 4.0 equiv, 1.0 M in hexanes) was added dropwise over 5 min. The reaction mixture was placed in a 0 °C ice-water bath and allowed to stir. After 1 h, the reaction mixture was diluted with $NaHCO_3$ (2 mL) and allowed to warm to room temperature. Once at room temperature, the reaction mixture was transferred to a separatory funnel, and the layers were separated. The aqueous layer was extracted with CH_2Cl_2 (3 × 2 mL). The combined organic extracts were dried with anhydrous Na_2SO_4, filtered, and concentrated *in vacuo* with the aid of a rotary evaporator. The residue was purified by flash column chromatography on silica gel (gradient elution: hexanes to 5% EtOAc in hexanes) to yield habiterpenol (**1**) as a colorless solid (2.9 mg, 43%).

^{1}H NMR (600 MHz, $CDCl_3$): δ 6.96 (d, *J* = 7.5 Hz, 1H), 6.55 (dd, *J* = 7.5, 2.5 Hz, 1H), 6.54 (s, 1H), 4.53 (br s, 1H), 2.93 (dd, *J* = 16.3, 6.6 Hz, 1H), 2.58 (d, *J* = 16.0 Hz, 1H), 2.25 (dt, *J* = 13.9, 4.1 Hz, 1H), 1.82 (dt, *J* = 12.9, 3.3 Hz, 1H), 1.72 (d, *J* = 6.9 Hz, 1H), 1.72–1.70 (m, 1H), 1.61–1.57 (m, 1H), 1.57–1.53 (m, 1H), 1.50–1.45 (m, 2H), 1.42–1.38 (m, 1H), 1.37–1.33 (m, 1H), 1.27–1.23 (m, 2H), 1.19–1.15 (m, 1H), 1.15–1.11 (m, 1H), 1.06 (s, 3H), 1.02 (td, *J* = 13.0, 4.2 Hz, 1H), 0.85 (s, 3H), 0.84–0.83 (m, 1H), 0.83–0.81 (m, 1H), 0.78 (s, 3H), 0.73 (s, 3H), 0.33 (s, 3H).

^{13}C NMR (151 MHz, $CDCl_3$): δ 154.1, 153.8, 135.9, 124.6, 112.6, 107.8, 63.1, 57.9, 56.8, 46.2, 43.0, 42.2, 40.1, 37.4, 37.4, 34.9, 33.5, 33.4, 33.3, 31.0, 21.5, 18.6, 18.3, 18.2, 16.4, 16.4.

IR (Diamond-ATR, neat) ṽ (cm^{-1}): 3342, 2932, 2849, 1609, 1466, 1387, 1365, 1262, 1217, 1190, 1131, 909, 807

HRMS (ESI): m/z: $[M+H]^+$ calc'd for $C_{26}H_{39}O^+$: 367.2995. Found: 367.2991.

Specific Rotation $[\alpha]^{23}_D$: −18.7 (*c* = 0.1, CH_3OH). lit.: $[\alpha]^{26}_D$: −26.7 (*c* = 0.1, CH_3OH).

Comparison of Natural and Synthetic 1

Table SI-3: Spectroscopic Comparison of Habiterpenol (**1**) by ^{1}H NMR.

Carbon #	This Report: Habiterpenol ($CDCl_3$)	Tomoda & Co-workers: Habiterpenol ($CDCl_3$)[3]	Absolute Difference
1α	2.93, dd, *J* = 16.3, 6.6 Hz	2.94, ddd, *J* = 16.0, 7.0, 1.0 Hz	0.01
1β	2.58, d, *J* = 16.0 Hz	2.58, d, *J* = 16.0 Hz	0.00
2	1.72, d, *J* = 6.9 Hz	1.72, d, *J* = 7.0 Hz	0.00
3	–	–	–
4	2.25, dt, *J* = 13.7, 4.1 Hz	2.25, dt, *J* = 14.0, 4.0 Hz	0.00
5	1.50–1.45, m	1.47, m	–
6	1.15–1.11, m	1.15, m	–
7	–	–	–
8α	1.82, dt, *J* = 12.9, 3.3 Hz	1.82, dt, *J* = 13.0, 3.0 Hz	0.00
8β	1.02, td, *J* = 13.0, 4.2 Hz	1.02, dt, *J* = 13.0, 4.0 Hz	0.00
9α	1.50–1.45, m	1.47, m	–
9β	1.27–1.23, m	1.26, m	–
10	0.83–0.81, m	0.82, m	–
11	–	–	–
12α	1.72–1.70, m	1.72, m	
12β	0.84–0.83, m	0.84, dt, *J* = 11.5, 2.5 Hz	–
13α	1.61–1.57, m	1.60, m	–
13β	1.42–1.38, m	1.40, m	–
14α	1.37–1.33, m	1.36, m	–
14β	1.19–1.15, m	1.15, m	–
15	–	–	–
16	1.06, s	1.06, s	0.00
17	0.33, s	0.33, s	0.00
18	0.73, s	0.73, s	0.00
19	0.78, s	0.78, s	0.00
20	0.85, s	0.85, s	0.00
1’	–	–	–
2’	6.96, d, *J* = 7.5 Hz	6.96, d, *J* = 7.5 Hz	0.00
3’	6.55, dd, *J* = 7.5, 2.5 Hz	6.55, dd, *J* = 7.5, 2.5 Hz	0.00
4’	–	–	–
5’	6.54, s	6.54, s	0.00
6’	–	–	–
4’-OH	4.53, s	4.67, s	0.14

The ^{1}H-NMR data for synthetic habiterpenol listed in the table above are reported in ppm relative to $CDCl_3$ calibrated to 7.26 ppm

Table SI-4: Spectroscopic Comparison of Habiterpenol (**1**) by ^{13}C NMR.

Carbon #	This Report: Habiterpenol ($CDCl_3$)	Tomoda & Co-workers: Habiterpenol ($CDCl_3$)[3]	Absolute Difference
1	31.0	30.9	0.1
2	63.1	63.1	0.0
3	46.2	46.3	0.1
4	34.9	34.9	0.0
5	18.2	18.2	0.0
6	57.9	57.9	0.0
7	37.4	37.4	0.0
8	43.0	43.0	0.0
9	18.3	18.4	0.1
10	56.8	56.8	0.0
11	37.4	37.4	0.0
12	40.1	40.1	0.0
13	18.6	18.6	0.0
14	42.2	42.2	0.0
15	33.3	33.3	0.0
16	33.5	33.5	0.0
17	16.4	16.43	0.03
18	16.4	16.42	0.02
19	21.5	21.5	0.0
20	33.4	33.4	0.0
1’	135.9	135.9	0.0
2’	124.6	124.6	0.0
3’	112.6	112.6	0.0
4’	154.1	154.2	0.1
5’	107.8	107.8	0.0
6’	153.8	153.8	0.0

The ^{13}C-NMR data for synthetic habiterpenol listed in the table above are reported in ppm relative to $CDCl_3$ calibrated to 77.0 ppm.

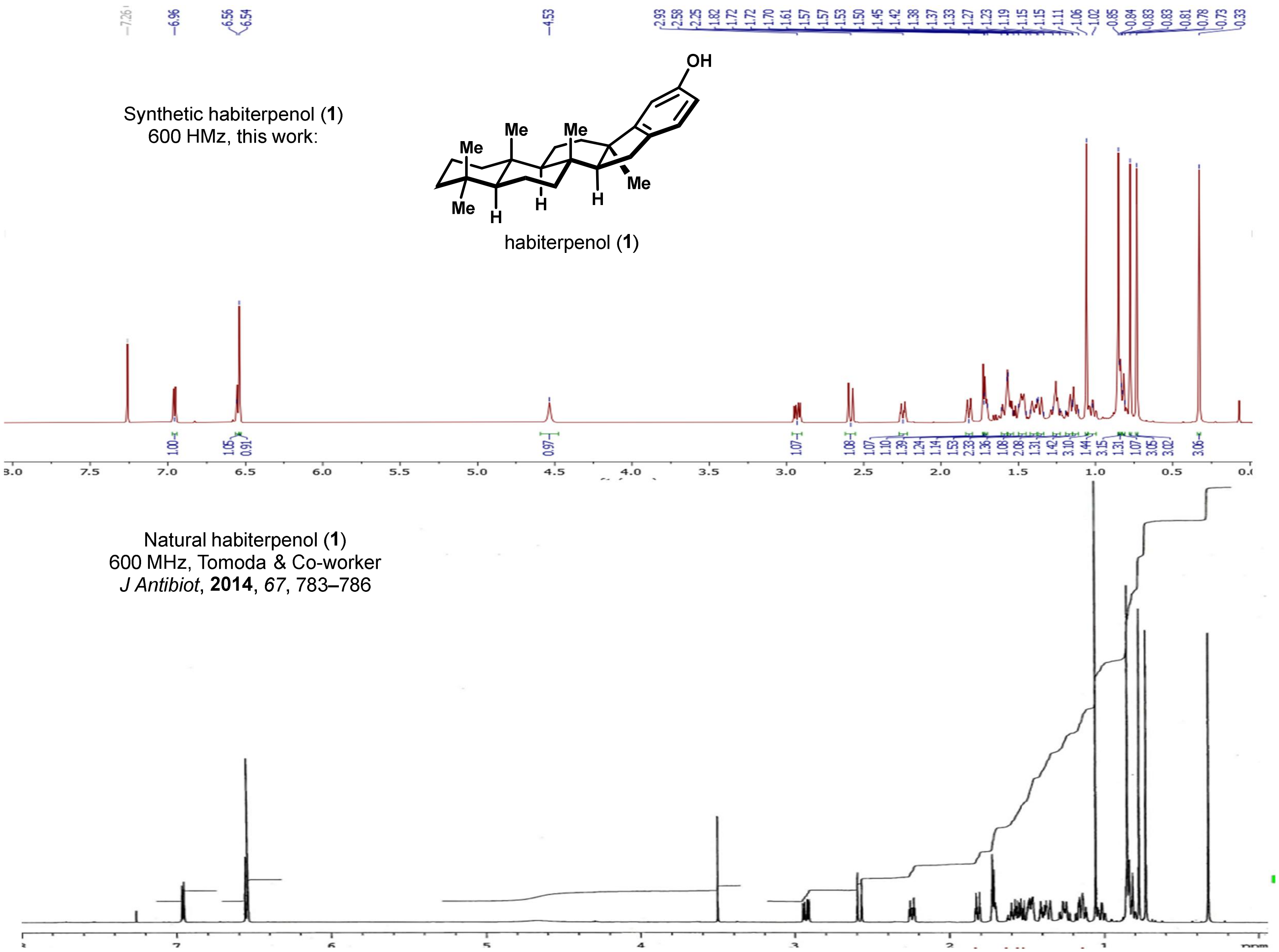

Synthetic habiterpenol (**1**)
600 HMz, this work:

habiterpenol (**1**)

Natural habiterpenol (**1**)
600 MHz, Tomoda & Co-worker
J Antibiot, **2014**, *67*, 783–786

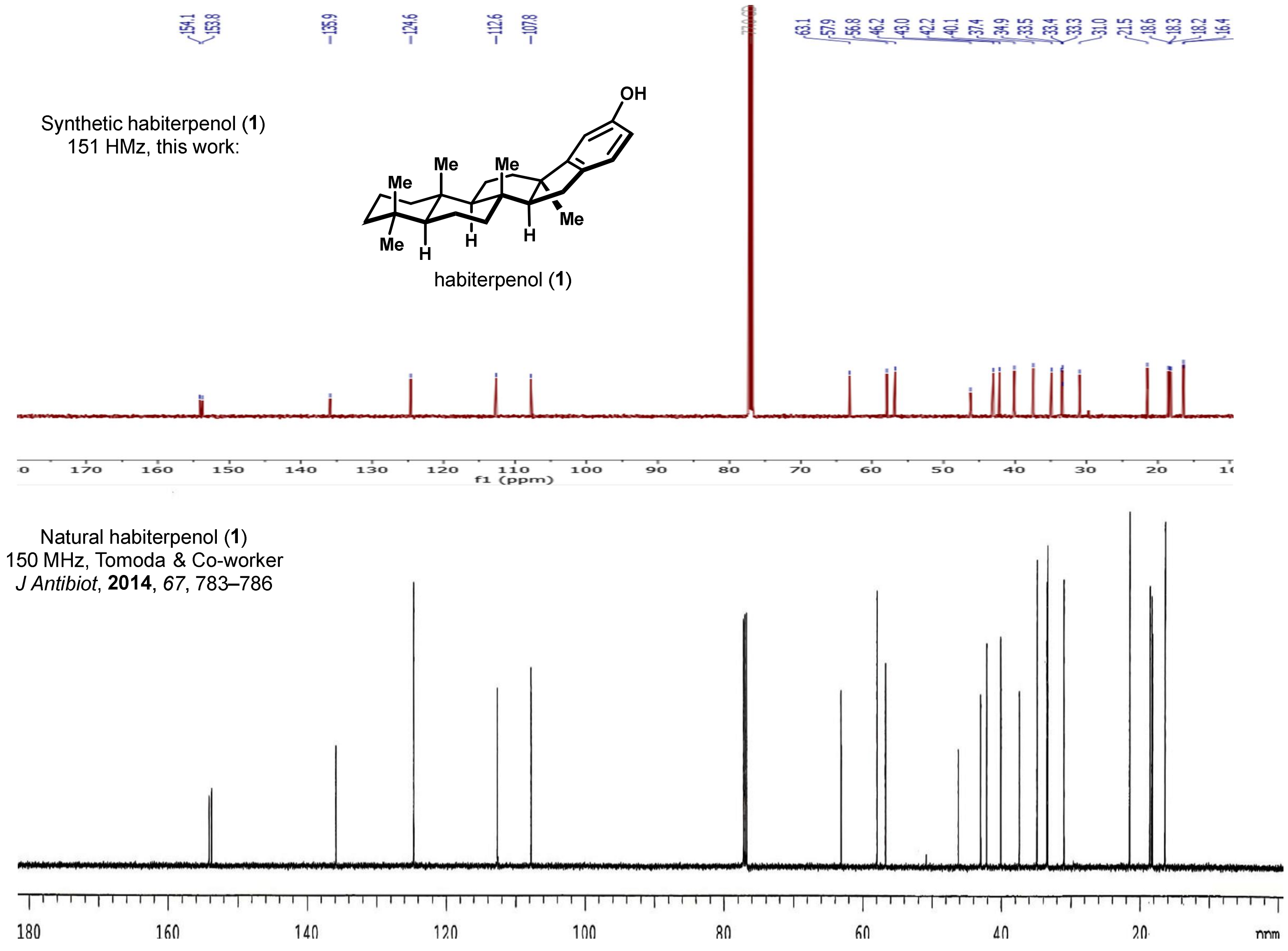
154.1
153.8
135.9
124.6
112.6
107.8
63.1
57.9
56.8
46.2
43.0
42.2
40.1
37.4
34.9
33.5
33.4
33.3
31.0
21.5
18.6
18.3
18.2
16.4
Synthetic habiterpenol (1)
151 HMz, this work:
OH
Me
Me
Me
Me
Me
H
H
H
habiterpenol (1)
f1 (ppm)
Natural habiterpenol (1)
150 MHz, Tomoda & Co-worker
J Antibiot, 2014, 67, 783–786
ppm

4. Synthesis toward dasyscyphin A (2)

Synthesis of nitrile (19)

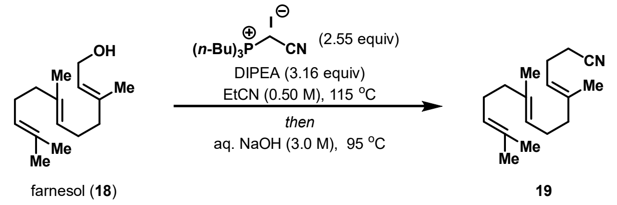

To an oven-dried 250 mL round-bottom flask equipped with a magnetic stir-bar was charged with **18** (10.0 g, 45.0 mmol, 1.0 equiv) and (cyanomethyl)tributylphosphonium iodide (42.3 g, 114.7 mmol, 2.55 equiv). The reaction vessel was sealed with a rubber septum and evacuated then backfilled with N_2 utilizing a dual manifold Schlenk line. This process was repeated three times. The flask was then attached with a N_2-filled reflux condenser. To the stirred reaction mixture, EtCN (90 mL, 0.50 M) and *N*,*N*-diisopropylethylamine (24.8 mL, 142 mmol, 3.16 equiv) were added. Then the reaction vessel was placed in a preheated oil bath at 115 °C. After 18 h, aq. NaOH (30 mL, 3.0 M) was added to the flask at 95 °C. After 24 h, the reaction vessel was removed from the oil bath and allowed to cool to room temperature. Once at room temperature, the reaction mixture was diluted with aq. HCl (70 mL, 3.0 M), transferred to a separatory funnel, and the layers were separated. The aqueous layer was extracted with EtOAc (3 x 80 mL). The combined organic extracts were washed with brine (100 mL), dried over anhydrous Na_2SO_4, filtered, and concentrated *in vacuo* with the aid of a rotary evaporator. The crude mixture was purified by flash column chromatography on silica gel (2% EtOAc in hexanes) to yield **19** as yellow oil (7.7 g, 70%).

All spectroscopic data for **19** was consistent with that which was previously reported.[4]

Synthesis of diol (SI-6)

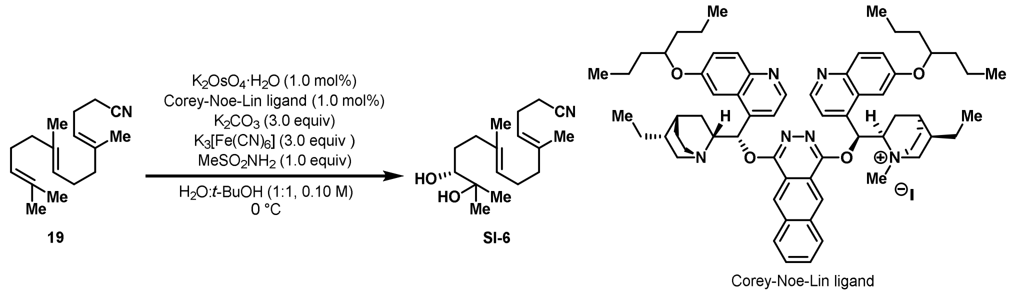

To an oven-dried 1 L round-bottom flask equipped with a magnetic stir-bar was charged with K_2CO_3 (16.9 g, 120.0 mmol, 3.0 equiv), $K_3Fe(CN)_6$ (40.2 g, 122 mmol, 3.0 equiv), $K_2OsO_4{\bullet}2H_2O$ (150 mg, 407 µmol, 1 mol%), Corey-Noe-Lin ligand (464 mg, 407 µmol, 1 mol%), and methylsulfonamide (3.9 g, 40.7 mmol, 1.0 equiv.). Water and *t*-BuOH (408 mL, 0.1 M, 1:1 v/v) were added and the resulting solution was placed in a sonication bath until a homogenous mixture was observed (*ca.* 20 min). The reaction vessel was removed from the sonication bath, transferred to a 0 °C ice-water bath with vigorous stirring, and **19** (10.0 g, 40.7 mmol, 1.0 equiv) was added. The reaction vessel was sealed with a rubber septum and stirred at 0 °C. After 6 h, the reaction mixture was diluted with sat. aq. Na_2SO_3 (200 mL) at 0 °C and allowed to warm to room temperature. After 45 min, the reaction mixture was diluted with EtOAc (300 mL) and

transferred to a separatory funnel. The layers were separated and the aqueous layer was extracted with EtOAc (3 x 300 mL). The combined organic extracts were washed with aq. NaOH (400 mL, 1.0 M), brine (400 mL), dried over anhydrous Na_2SO_4, filtered, and concentrated *in vacuo* with the aid of a rotary evaporator. The residue was purified by flash column chromatography on silica gel (gradient elution: 25% to 60% EtOAc in hexanes) to yield **SI-6** as a yellow oil (5.7 g, 50%). The *e.e.* of the product was determined by HPLC after derivatization with benzoyl anhydride: To a vial were added product **SI-6** (1.0 equiv), 4-dimethylaminopyridine (1.5 equiv), benzoyl anhydride (1.5 equiv) and CH_2Cl_2 (0.10 M). The reaction was stirred at room temperature for 2 h. After completion, the reaction mixture was purified by preparative thin-layer chromatography to afford the corresponding benzoylated compound. HPLC analysis was performed with a Chiralcel chiral OJ-H column. The enantiomeric excess of benzoate was determined by elution with 96:4 mixture of *n*-hexane and 2-propanol (1.0 mL/min). Retention times for the enantiomers of benzoate were 15.98 min (minor) and 19.79 min (major), respectively; 97% *e.e.*

^{1}H NMR (600 MHz, $CDCl_3$): δ 5.18–5.12 (m, 2H), 3.32 (dd, *J* = 10.6, 1.2 Hz, 1H), 2.37–2.32 (m, 4H), 2.23 (ddd, *J* = 14.0, 8.7, 5.6 Hz, 1H), 2.10 (tt, *J* = 14.6, 7.2 Hz, 2H), 2.07–2.02 (m, 3H), 1.63 (s, 3H), 1.60 (s, 3H), 1.59–1.55 (m, 1H), 1.39 (dddd, *J* = 15.7, 10.5, 8.7, 5.6 Hz, 1H), 1.18 (s, 3H), 1.14 (s, 3H).

^{13}C NMR (151 MHz, $CDCl_3$): δ 139.0, 135.3, 124.5, 120.4, 119.8, 78.2, 73.0, 39.5, 36.8, 29.8, 26.5, 26.1, 24.0, 23.4, 17.8, 16.2, 16.1.

IR (Diamond-ATR, neat) ṽ (cm^{-1}): 3437, 2973, 2930, 1439, 1371, 1273, 1259, 1158, 1073, 929.

HRMS (ESI): m/z: $[M+H]^+$ calc'd for $C_{17}H_{30}NO_2^+$: 280.2271. Found: 280.2272.

Specific Rotation $[\alpha]^{23}_D$: +20.5 (*c* = 1.0, $CHCl_3$).

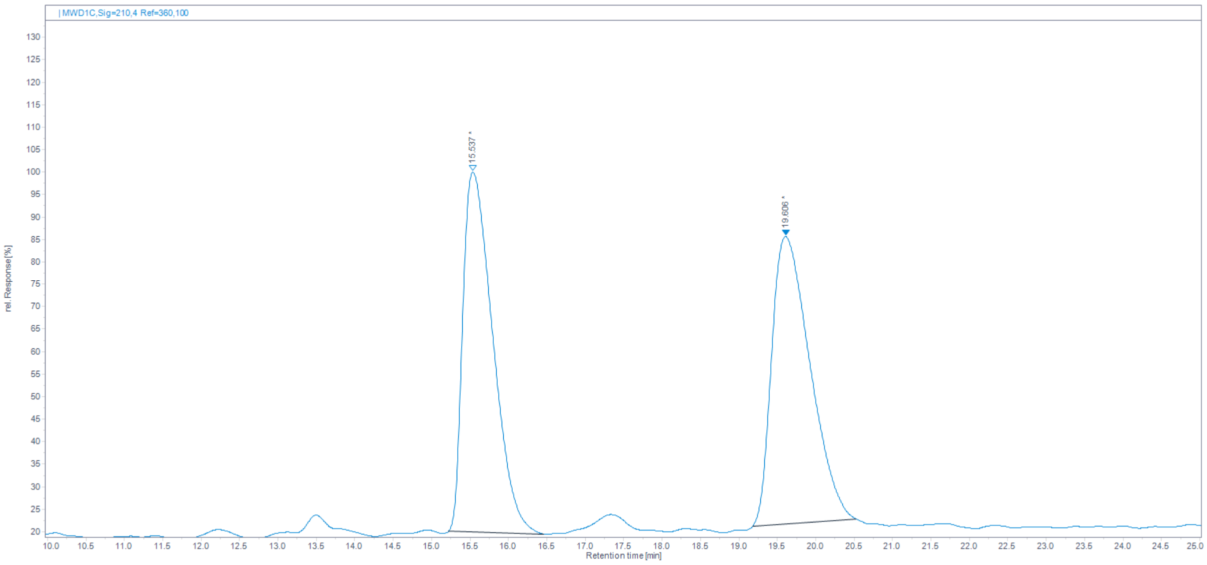

#	RT (min)	Area%
1	15.537	49.464
2	19.606	50.536

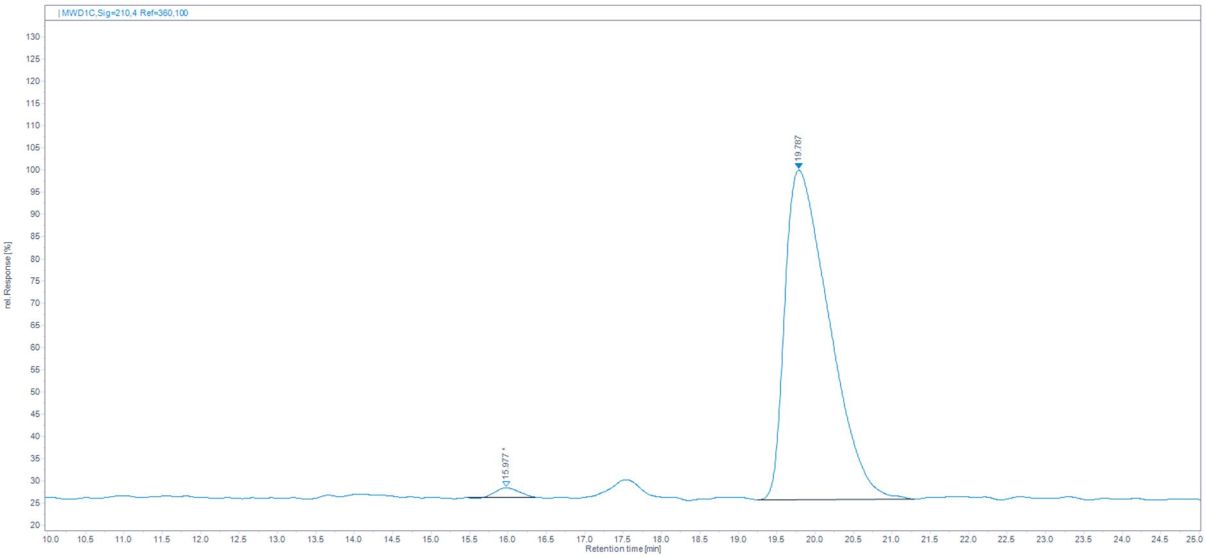

#	RT (min)	Area%
1	15.977	1.477
2	19.787	98.523

Synthesis of epoxide (8)

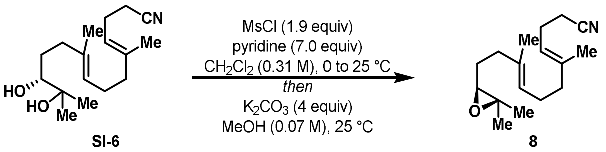

SI-6 8

To a flame-dried 2 L round-bottom flask equipped with a magnetic stir-bar was charged with **SI-6** (13.4 g, 47.9 mmol, 1.0 equiv). The reaction vessel was sealed with a rubber septum and evacuated then backfilled with N_2 utilizing a dual manifold Schlenk line. This process was repeated three times. The reaction vessel was placed in a 0 °C ice-water bath. To the reaction mixture, pyridine (56.8 mL, 714 mmol, 14.9 equiv) and CH_2Cl_2 (117 mL, 0.31 M) were added. The reaction mixture was cooled to 0 °C in an ice-water bath, and methanesulfonyl chloride (5.93 mL, 76.7 mmol, 1.6 equiv) was added dropwise over 5 min. After 45 min, the reaction vessel was removed from the ice-water bath and was allowed to stir at room temperature. After 21 h, K_2CO_3 (64.2 g, 465 mmol, 9.7 equiv.) and MeOH (621 mL, 0.07 M) were added and the mixture was stirred for an additional 5.5 h. The reaction mixture was then diluted with H_2O (600 mL), transferred to a separatory funnel, and the layers were separated. The aqueous layer was extracted with CH_2Cl_2 (3 x 300 mL). The combined organic extracts were washed with brine (600 mL), dried over anhydrous Na_2SO_4, filtered, and concentrated *in vacuo* with the aid of a rotary evaporator. The residue was purified by flash column chromatography on silica gel (50% EtOAc in hexanes) to yield epoxide **8** as a yellow oil (10.2 g, 81%).

^{1}H NMR (600 MHz, $CDCl_3$): δ 5.16–5.13 (m, 2H), 2.70 (t, *J* = 6.2 Hz, 1H), 2.36–2.33 (m, 4H), 2.18–2.05 (m, 4H), 2.05–2.01 (m, 2H), 1.65 (s, 3H), 1.64–1.59 (m, 2H), 1.62 (s, 3H), 1.30 (s, 3H), 1.26 (s, 3H).

^{13}C NMR (151 MHz, $CDCl_3$): δ 139.2, 134.6, 124.5, 120.3, 119.8, 64.3, 58.5, 39.6, 36.4, 27.6, 26.5, 25.1, 24.1, 18.9, 17.8, 16.3, 16.2.

IR (Diamond-ATR, neat) ṽ (cm^{-1}): 2953, 2920, 2855, 1451, 1373, 1250, 1114, 802.

Specific Rotation $[\alpha]^{23}_{D}$: −3.2 (*c* = 1.0, $CHCl_3$).

All spectroscopic data for **8** was consistent with that which was previously reported.[4]

Synthesis of alcohol (20)

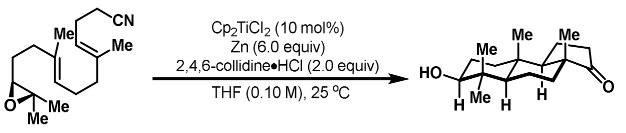

To a flame-dried 500 mL round-bottom flask equipped with a magnetic stir-bar was charged with Cp_2TiCl_2 (343 mg, 1.38 mmol, 10 mol%), activated Zn (5.40 g, 82.6 mmol, 6.0 equiv), and 2,4,6-collidine hydrochloride (4.34 g, 27.5 mmol, 2.0 equiv). The reaction flask was transferred to a N_2-filled glovebox and THF (138 mL, 0.10 M) was added. The resulting solution was allowed to stir for 10 min to give a green heterogenous mixture. To the reaction mixture was added **8** (3.60 g, 13.8 mmol, 1.0 equiv). The reaction vessel was sealed with a rubber septum, removed from the glovebox, and allowed to stir at room temperature. After 18 h, the reaction mixture was diluted with aq. KH_2PO_4 solution (10% w/v, 138 mL), and the resulting solution was stirred. After 30 min, the reaction mixture was filtered over a packed pad of Celite (150 mL fritted funnel, 18 mm Celite powder) and washed with EtOAc (50 mL). The filtrate was transferred to a separatory funnel and the layers were separated. The aqueous layer was extracted with EtOAc (3 x 50 mL). The combined organic extracts were washed with sat. aq. HCl (100 mL, 1.0 M), brine (100 mL), dried over anhydrous Na_2SO_4, filtered, and concentrated *in vacuo* with the aid of a rotary evaporator. The residue was purified by flash column chromatography on silica gel (gradient elution: 14% EtOAc and 14% CH_2Cl_2 in hexanes to 17% EtOAc and 17% CH_2Cl_2 in hexanes) to yield **20** as a colorless solid (1.80 g, 49%).

^{1}H NMR (600 MHz, $CDCl_3$): δ 3.20 (dd, *J* = 11.3, 4.9 Hz, 1H), 2.41 (ddd, *J* = 19.3, 8.9, 1.1 Hz, 1H), 2.05 (dt, *J* = 19.2, 8.9 Hz, 1H), 1.81–1.73 (m, 2H), 1.72–1.64 (m, 3H), 1.63–1.58 (m, 2H), 1.48 (dddd, *J* = 13.2, 13.2, 13.2, 3.5 Hz, 1H), 1.26–1.20 (m, 2H), 1.08 (dt, *J* = 13.7, 4.1 Hz, 1H), 0.97 (s, 3H), 0.96 (s, 3H), 0.93 (s, 3H), 0.79 (s, 3H), 0.78–0.76 (m, 1H).

^{13}C NMR (151 MHz, $CDCl_3$): δ 221.1, 79.0, 58.3, 56.0, 48.3, 39.0, 37.6, 37.5, 35.8, 33.4, 28.3, 27.0, 18.5, 18.2, 17.0, 16.6, 15.3.

IR (Diamond-ATR, neat) ṽ (cm^{-1}): 3491, 2961, 2916, 2838, 2362, 2325, 1726, 1475, 1451, 1377, 1258, 1090, 1061, 1003, 839.

HRMS (ESI): m/z: $[M+H]^+$ calc'd for $C_{17}H_{29}O_2$: 265.2162. Found: 265.2162.

Specific Rotation $[\alpha]^{23}_{D}$: −29.1 (*c* = 0.7, $CHCl_3$).

Synthesis of ketone (7)

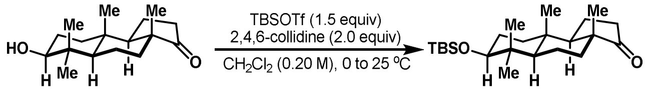

20 7

To an oven-dried 250 mL round-bottom flask equipped with a magnetic stir-bar was charged with **20** (1.80 g, 6.81 mmol, 1.0 equiv). The reaction vessel was sealed with a rubber septum and evacuated then backfilled with N_2 utilizing a dual manifold Schlenk line. This process was repeated three times. The reaction vessel was placed in a 0 °C ice-water bath. To the stirred reaction mixture, CH_2Cl_2 (34 mL, 0.2 M) and 2,4,6-collidine (1.80 mL, 13.6 mmol, 2.0 equiv) were added. Following this, *tert*-butyldimethylsilyl trifluoromethanesulfonate (2.35 mL, 10.2 mmol, 1.5 equiv) was added to the reaction mixture dropwise over 3 min. Then the reaction vessel was removed from the ice-water bath and allowed to stir at room temperature. After 22 h, the reaction mixture was diluted with sat. aq. NH_4Cl (20 mL). The resulting mixture was transferred to a separatory funnel and the layers were separated. The aqueous layer was extracted with CH_2Cl_2 (3 x 15 mL). The combined organic extracts were washed with aq. HCl (30 mL, 1.0 M), brine (30 mL), dried over anhydrous Na_2SO_4, filtered, and concentrated *in vacuo* with the aid of a rotary evaporator. The residue was purified by flash column chromatography on silica gel (gradient elution: 1% EtOAc to 2% EtOAc in hexanes) to yield **7** as a colorless solid (2.10 g, 81%).

^{1}H NMR (600 MHz, $CDCl_3$): δ 3.17 (dd, *J* = 11.6, 4.6 Hz, 1H), 2.41 (ddd, *J* = 19.2, 8.8, 1.6 Hz, 1H), 2.05 (dt, *J* = 19.3, 8.9 Hz, 1H), 1.80–1.73 (m, 2H), 1.72–1.66 (m, 2H), 1.65–1.59 (m, 1H), 1.57–1.50 (m, 2H), 1.46 (dddd, *J* = 13.2, 13.2, 13.2, 3.4 Hz, 1H), 1.25–1.19 (m, 2H), 1.08–1.02 (m, 1H), 0.96 (s, 3H), 0.93 (s, 3H), 0.89 (s, 3H), 0.87 (s, 9H), 0.77–0.74 (m, 4H), 0.02 (s, 3H), 0.02 (s, 3H).

^{13}C NMR (151 MHz, $CDCl_3$): δ 221.1, 79.6, 58.5, 56.1, 48.3, 39.5, 37.5, 37.4, 35.8, 33.5, 28.7, 27.5, 26.0, 18.7, 18.2, 17.0, 16.6, 15.8, −3.6, −4.8.

IR (Diamond-ATR, neat) ṽ (cm^{-1}): 2953, 2929, 2863, 2362, 2329, 1738, 1455, 1254, 1098, 1065, 1008, 868, 839.

HRMS (ESI): m/z: $[M+H]^+$ calc'd for $C_{23}H_{43}O_2Si$: 379.3027. Found: 379.3026.

Specific Rotation $[\alpha]^{23}_D$: −66.5 (*c* = 1.0, $CHCl_3$).

Synthesis of *epi*-ketone (6)

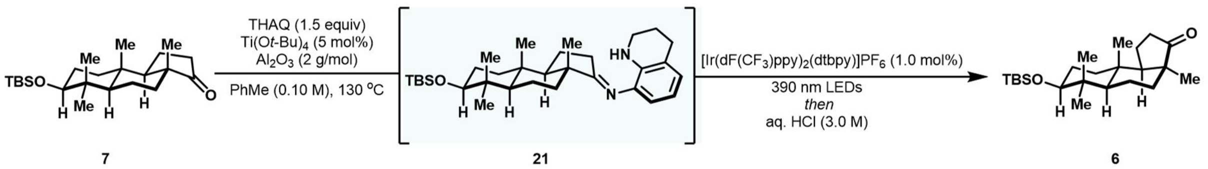

7 21 6

Inside a N_2-filled glovebox, a flame-dried 250 mL round-bottom flask equipped with a magnetic stir-bar was charged with **7** (2.05 g, 5.41 mmol, 1.0 equiv), Al_2O_3 (10.8 g, 2.0

g/mmol), and PhMe (54 mL, 0.10 M). To the stirred reaction mixture was added 1,2,3,4-tetrahydroquinolin-8-amine (1.20 g, 8.12 mmol, 1.5 equiv) and Ti(O*t*-Bu)$_4$ (106 µL, 271 µmol, 5.0 mol%). The reaction flask was sealed with a rubber septum, removed from the glovebox, and equipped with a Dean-Stark apparatus fitted with a reflux condenser and a N_2-filled balloon. The reaction apparatus was placed in a preheated oil bath at 130 °C. After 18 h, the reaction mixture was removed from the oil bath and allowed to cool to room temperature. Once at room temperature, the reaction mixture was filtered over a short plug of SiO_2 (150 mL fritted funnel, 3 cm SiO_2, treated with 5% Et_3N in hexanes) and the reaction flask was rinsed with hexanes (3 x 15 mL). The SiO_2 plug was eluted with 25% EtOAc in hexanes (100 mL) and the filtrate was concentrated *in vacuo* with the aid of a rotary evaporator. The residue was transferred to a separate flame-dried 500 mL flask using CH_2Cl_2 (100 mL) and concentrated *in vacuo* with the aid of a rotary evaporator. The crude mixture was successively azeotroped with benzene (50 mL) and CH_2Cl_2 (3 x 50 mL), then dried under high vacuum utilizing a dual-manifold Schlenk line to yield **21** as a pale-yellow solid, which was used without further purification.

Note: The PhMe and Et_3N must be completely removed from the imine intermediate before proceeding to the subsequent operation.

Inside a N_2-filled glovebox, the above 500 mL reaction flask equipped with a magnetic stir-bar was charged with [Ir(dF(CF$_3$)ppy)$_2$(dtbpy)]PF$_6$ (61 mg, 54 µmol, 1.0 mol%) and 1,2-dichloroethane (108 mL, 0.05 M). The reaction flask was sealed with a rubber septum and removed from the glovebox. The reaction vessel was subjected to LED irradiation utilizing two 30 W Kessil PR-160L 390 nm LEDs at 100% intensity at distances of 10 cm. To the flask was added aq. HCl (108 mL, 3.0 M) and placed in a preheated oil bath at 60 ºC. After 4 h, the reaction mixture was removed from the oil bath and allowed to cool to room temperature. Once at room temperature, the reaction mixture was transferred to a separatory funnel and the layers were separated. The aqueous layer was extracted with CH_2Cl_2 (3 x 50 mL). The combined organic extracts were washed with brine (50 mL), dried over anhydrous Na_2SO_4, filtered, and concentrated *in vacuo* with the aid of a rotary evaporator. The residue was purified by flash column chromatography on silica gel (gradient elution: hexanes to 10% EtOAc in hexanes) to yield **6** as a colorless solid (1.42 g, 69%).

^{1}H NMR (600 MHz, $CDCl_3$): δ 3.18 (dd, *J* = 11.3, 4.5 Hz, 1H), 2.39–2.32 (m, 1H), 2.31–2.27 (m, 1H), 2.07 (dt, *J* = 18.9, 9.6 Hz, 1H), 2.05–1.98 (m, 1H), 1.82–1.76 (m, 1H), 1.74 (dt, *J* = 13.4, 3.4 Hz, 1H), 1.57–1.40 (m, 4H), 1.19–1.07 (m, 2H), 1.01 (dd, *J* = 13.4, 3.5 Hz, 1H), 0.97 (s, 3H), 0.90 (s, 3H), 0.87 (s, 9H), 0.75 (dd, *J* = 11.8, 1.6 Hz, 1H), 0.71 (s, 3H), 0.63 (s, 3H), 0.02 (s, 3H), 0.02 (s, 3H).

^{13}C NMR (151 MHz, $CDCl_3$): δ 221.5, 79.7, 58.1, 52.2, 48.8, 39.4, 38.7, 37.0, 34.4, 31.7, 29.1, 28.4, 27.5, 26.0, 19.3, 18.4, 18.2, 16.4, 15.2, −3.7, −4.8.

IR (Diamond-ATR, neat) ṽ (cm^{-1}): 2953, 2916, 2852, 1730, 1244, 1099, 1059, 839.

HRMS (ESI): m/z: [M+H]$^+$ calc'd for $C_{23}H_{43}O_2Si$: 379.3027. Found: 379.3019.

Specific Rotation $[\alpha]^{23}_D$: +16.2 (*c* = 1.0, $CHCl_3$).

Synthesis of alkene alcohol (22)

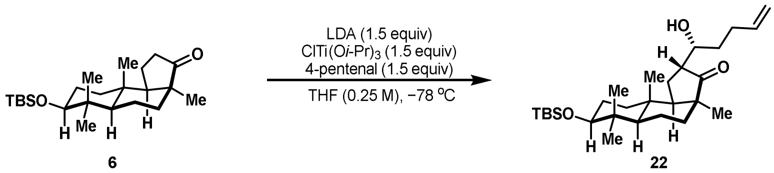

To a flame-dried 50 mL round-bottom flask equipped with a magnetic stir-bar was charged with *i*-Pr_2NH (224 μL, 1.58 mmol, 1.5 equiv) and THF (4.2 mL, 0.25 M). The reaction vessel was placed in a −78 °C dry ice/acetone bath. To the stirred reaction mixture, *n*-butyllithium (634 μL, 1.58 mmol, 1.5 equiv, 2.5 M in hexanes) was added over 2 min and the reaction mixture was allowed to stir for 1 h at −78 °C to form the LDA solution.

To a separate flame-dried 25 mL round-bottom flask was added **6** (400.0 mg, 1.06 mmol, 1.0 equiv) and THF (4.2 mL, 0.25 M). The resulting solution was added dropwise to the LDA solution over 2 min at −78 °C. After stirring at −78 °C for 2 h, chlorotitanium triisopropoxide (413 mg, 1.58 mmol, 1.5 equiv) in THF (1.58 mL,1.0 M) was added to the reaction mixture over 1 min. After 10 min, 4-pentenal (133 mg, 1.58 mmol, 1.5 equiv) was added and the reaction was stirred at –78 °C. After 1 h, the reaction mixture was diluted with sat. aq. NH_4Cl (10 mL). The reaction vessel was removed from the dry ice/acetone bath and allowed to warm to room temperature. Once at room temperature, the resulting mixture was filtered over a packed pad of Celite (60 mL fritted funnel, 18 mm Celite powder) and washed with EtOAc (20 mL). The filtrate was transferred to a separatory funnel, and the layers were separated. The organic extract was washed with brine (20 mL), dried over anhydrous Na_2SO_4, filtered, and concentrated *in vacuo* with the aid of a rotary evaporator. The residue was purified by flash column chromatography on silica gel (gradient elution: 2% to 2.5% EtOAc in hexanes) to yield **22** as a colorless solid (364 mg, 75%).

^{1}H NMR (600 MHz, $CDCl_3$): δ 5.83 (ddt, *J* = 16.9, 10.2, 6.6 Hz, 1H), 5.05 (ddt, *J* = 17.2, 1.8, 1.4 Hz, 1H), 4.97 (dd, *J* = 10.2, 1.0 Hz, 1H), 3.98 (s, 1H), 3.55 (td, *J* = 9.0, 2.6 Hz, 1H), 3.20 (dd, *J* = 11.2, 4.6 Hz, 1H), 2.34 (ddt, *J* = 13.8, 2.2, 2.0 Hz, 1H), 2.27 (ddt, *J* = 15.2, 9.2, 5.6 Hz, 1H), 2.23–2.11 (m, 2H), 1.93 (dd, *J* = 14.1, 9.4 Hz, 1H), 1.77 (dt, *J* = 13.3, 3.5 Hz, 1H), 1.70–1.59 (m, 2H), 1.57–1.52 (m, 2H), 1.52–1.43 (m, 3H), 1.20 (td, *J* = 13.5, 5.6 Hz, 1H), 1.13–1.02 (m, 2H), 0.99 (s, 3H), 0.91 (s, 3H), 0.88 (s, 9H), 0.77 (dd, *J* = 11.8, 2.4 Hz, 1H), 0.72 (s, 3H), 0.67 (s, 3H), 0.04 (s, 3H), 0.03 (s, 3H).

^{13}C NMR (151 MHz, $CDCl_3$): δ 225.6, 138.6, 114.9, 79.6, 74.8, 56.8, 51.9, 51.8, 49.5, 39.4, 39.0, 36.9, 35.0, 32.5, 30.1, 29.2, 28.5, 27.6, 26.0, 23.7, 19.5, 18.2, 16.6, 16.5, −3.6, −4.8.

IR (Diamond-ATR, neat) ṽ (cm^{-1}): 3499, 2937, 2855, 2366, 2338, 1722, 1467, 1381, 1361, 1250, 1106, 880, 831.

HRMS (ESI): m/z: [M+H]$^+$ calc'd for $C_{28}H_{51}O_3Si$: 463.3602. Found: 463.3599.

Specific Rotation $[\alpha]^{26}_D$: +37.6 (*c* = 1.0, $CHCl_3$).

Synthesis of OTES alkene (SI-7)

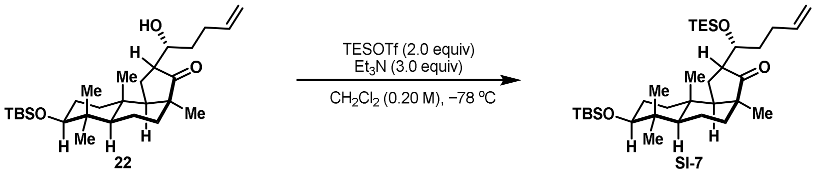

To a flame-dried 10 mL round-bottom flask equipped with a magnetic stir-bar was charged with **22** (281 mg, 0.61 mmol, 1.0 equiv). The reaction vessel was sealed with a rubber septum and evacuated then backfilled with N_2 utilizing a dual manifold Schlenk line. This process was repeated three times. The reaction vessel was placed in a –78 °C dry ice/acetone bath and charged with CH_2Cl_2 (3.0 mL, 0.20 M) and Et_3N (253 µL, 1.82 mmol, 3.0 equiv). To the stirred reaction mixture, triethylsilyl trifluoromethanesulfonate (272 µL, 1.21 mmol, 2.0 equiv) was added dropwise over 2 min. The reaction was stirred at –78 °C. After 1 h, the reaction mixture was diluted with sat. aq. NH_4Cl (10 mL), transferred to a separatory funnel and the layers were separated. The aqueous layer was extracted with CH_2Cl_2 (3 x 5 mL). The combined organic extracts were washed with brine (10 mL), dried over anhydrous Na_2SO_4, filtered, and concentrated *in vacuo* with the aid of a rotary evaporator. The residue was purified by flash column chromatography on silica gel (2% EtOAc in hexanes) to yield **SI-7** as a colorless solid (332 mg, 95%).

^{1}H NMR (600 MHz, $CDCl_3$): δ 5.80 (ddt, *J* = 16.9, 10.2, 6.6 Hz, 1H), 5.00 (ddt, *J* = 17.1, 1.7, 1.6 Hz, 1H), 4.93 (ddt, *J* = 10.2, 1.5, 1.4 Hz, 1H), 3.87 (td, *J* = 6.4, 4.7 Hz, 1H), 3.19 (dd, *J* = 11.3, 4.5 Hz, 1H), 2.47 (td , *J* = 11.1, 9.3, 4.8 Hz, 1H), 2.32 (ddt, *J* = 13.6, 2.4, 2.0 Hz, 1H), 2.15–2.07 (m, 2H), 1.96 (ddd, *J* = 14.0, 11.0, 7.8 Hz, 1H), 1.81 (dd, *J* = 13.8, 9.5 Hz, 1H), 1.78–1.71 (m, 2H), 1.60–1.52 (m, 2H), 1.51–1.46 (m, 2H), 1.41 (ddt, *J* = 13.5, 5.4, 2.3 Hz, 1H), 1.17 (td, *J* = 13.4, 5.5 Hz, 1H), 1.12–1.00 (m, 2H), 0.96–0.92 (m, 12H), 0.90 (s, 3H), 0.87 (s, 9H), 0.75 (dd, *J* = 12.0, 1.8 Hz, 1H), 0.71 (s, 3H), 0.68 (s, 3H), 0.59 (q, *J* = 7.9 Hz, 6H), 0.03 (s, 3H), 0.03 (s, 3H).

^{13}C NMR (151 MHz, $CDCl_3$): δ 220.3, 138.6, 114.7, 79.8, 72.9, 56.7, 52.0, 51.2, 49.0, 39.4, 39.3, 37.0, 33.8, 32.9, 30.2, 29.2, 27.7, 27.2, 26.0, 22.9, 19.6, 18.2, 16.6, 16.5, 7.1, 5.2, −3.7, −4.8.

IR (Diamond-ATR, neat) ṽ (cm^{-1}): 2954, 1718, 1466, 1361, 1250, 1093, 1077, 1002, 881, 832.

HRMS (ESI): m/z: $[M+H]^+$ calc'd for $C_{34}H_{65}O_3Si_2$: 577.4467. Found: 577.4464.

Specific Rotation $[\alpha]^{25}_D$: +36.0 (*c* = 1.0, $CHCl_3$).

Synthesis of aldehyde (23)

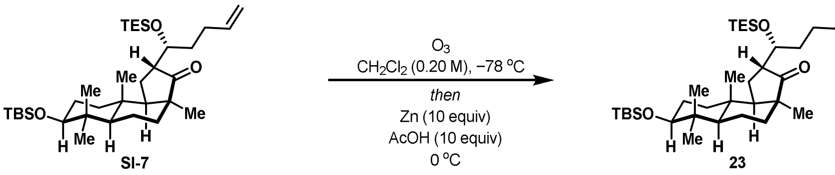

To an oven dried 25 mL round-bottom flask equipped with a magnetic stir-bar was charged with **SI-7** (300.0 mg, 0.52 mmol, 1.0 equiv) and CH_2Cl_2 (5.2 mL, 0.10 M). The reaction vessel was placed in a –78 °C dry ice/acetone bath. O_3 was bubbled through the reaction mixture until a blue colored solution was observed. After 10 min, N_2 was bubbled through the reaction solution for 10 min. To the resulting reaction mixture was added Zn (340 mg, 5.20 mmol, 10.0 equiv) and AcOH (298 μL, 5.20 mmol, 10.0 equiv). The reaction vessel was placed in a 0 °C ice-water bath and stirred. After 4 h, the reaction mixture was filtered over a packed pad of Celite (30 mL fritted funnel, 18 mm Celite powder) and eluted with CH_2Cl_2 (20 mL). The filtrate was transferred to a separatory funnel and the organic layer was washed with sat. aq. $NaHCO_3$ (20 mL), brine (30 mL), dried over anhydrous Na_2SO_4, filtered, and concentrated *in vacuo* with the aid of a rotary evaporator. The residue was purified by flash column chromatography on silica gel (gradient elution: 25% to 33% EtOAc in hexanes) to yield **23** as a colorless oil (273 mg, 91%).

^{1}H NMR (600 MHz, $CDCl_3$): 9.78 (t, *J* = 1.5 Hz 1H), 3.92 (dt *J* = 6.2, 5.9 Hz, 1H), 3.20 (dd, *J* = 11.2, 4.6 Hz, 1H), 2.57–2.47 (m, 2H), 2.42 (td, *J* = 10.0, 5.2 Hz, 1H), 2.31 (ddt, *J* = 13.8, 2.3, 1.9 Hz, 1H), 1.96 (ddd, *J* = 14.0, 10.9, 7.8 Hz, 1H), 1.91–1.80 (m, 3H), 1.77 (dt, *J* = 13.2, 3.4 Hz, 1H), 1.55–1.52 (m, 1H), 1.52–1.47 (m, 2H), 1.43 (ddt, *J* = 13.0, 5.8, 2.6 Hz, 1H), 1.18 (td, *J* = 13.3, 5.8 Hz, 1H), 1.11–1.00 (m, 2H), 0.97–0.93 (m, 12H), 0.91 (s, 3H), 0.88 (s, 9H), 0.76 (dd, *J* = 11.5, 1.7 Hz, 1H), 0.72 (s, 3H), 0.68 (s, 3H), 0.60 (q, *J* = 7.9 Hz, 6H), 0.04 (s, 3H), 0.03 (s, 3H).

^{13}C NMR (151 MHz, $CDCl_3$): δ 220.1, 202.3, 79.7, 72.4, 56.6, 51.9, 51.3, 49.0, 40.4, 39.4, 39.2, 37.0, 32.9, 29.2, 27.6, 27.3, 27.0, 26.0, 22.7, 19.5, 18.2, 16.6, 16.5, 7.0, 5.1, −3.7, −4.8.

IR (Diamond-ATR, neat) ṽ (cm^{-1}): 2947, 2878, 1728, 1466, 1391, 1365, 1250, 1100, 1002, 881, 832.

HRMS (ESI): m/z: $[M+H]^+$ calc'd for $C_{33}H_{63}O_4Si_2$: 579.4259. Found: 579.4248.

Specific Rotation $[\alpha]^{26}_D$: +31.4 (*c* = 1.0, $CHCl_3$).

Synthesis of diol (SI-8)

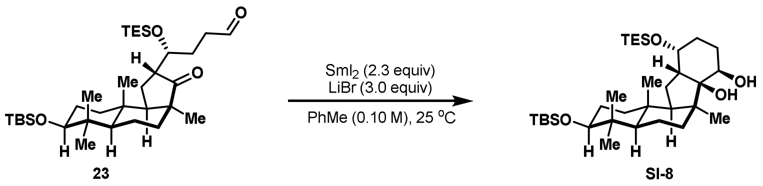

Preparation of dried LiBr: To a 25 mL round-bottom flask was added LiBr (200 mg). The reaction vessel was sealed with a rubber septum, then evacuated and backfilled with N_2 utilizing a dual manifold Schlenk line. This process was repeated three times. The LiBr was dried under vacuum by heating with a butane torch for 1 min. After cooling to room temperature, the LiBr was stored in a N_2-filled glovebox.

In a N_2-filled glovebox, a flame-dried 50 mL round-bottom flask equipped with a magnetic stir-bar was charged with dried LiBr (67 mg, 0.77 mmol, 3.0 equiv), PhMe (12.8 mL, 0.20 M), and SmI_2 (5.9 mL, 0.59 mmol, 2.3 equiv, 0.10 M in THF). The reaction mixture was stirred for 10 min and resulted in a blue solution. To a separate flame-dried 100 mL round-bottom flask was added **23** (148 mg, 0.26 mmol, 1.0 equiv) and PhMe (12.8 mL, 0.20 M). The resulting solution was added dropwise over 10 min to the stirred SmI_2 solution at room temperature (final reaction concentration: 0.10 M). The reaction flask was sealed with a rubber septum and stirred at room temperature. After 30 min, the reaction vessel was removed from the glovebox and diluted with sat. aq. $NaHCO_3$ (10 mL). The resulting mixture was transferred to a separatory funnel and the layers were separated. The aqueous layer was extracted with EtOAc (3 x 5 mL). The combined organic extracts were washed with brine (10 mL), dried over anhydrous Na_2SO_4, filtered, and concentrated *in vacuo* with the aid of a rotary evaporator. The residue was purified by flash column chromatography on silica gel (7% EtOAc in hexanes) to yield **SI-8** as a colorless solid (70 mg, 47%).

^{1}H NMR (600 MHz, $CDCl_3$): δ 3.94 (dt, *J* = 9.6, 4.9 Hz, 1H), 3.88 (dd, *J* = 10.3, 4.9 Hz, 1H), 3.17 (dd, *J* = 11.3, 4.1 Hz, 1H), 2.32–2.25 (m, 2H), 1.78–1.73 (m, 2H), 1.69–1.53 (m, 9H), 1.49 (dd, *J* = 13.9, 7.1 Hz, 1H), 1.44–1.40 (m, 1H), 1.33–1.29 (m, 1H), 1.26–1.23 (m, 2H), 1.18–1.12 (m, 4H), 0.94 (t, *J* = 8.0 Hz, 9H), 0.88 (s, 9H), 0.86 (s, 9H), 0.63–0.53 (m, 6H), 0.03 (s, 3H), 0.02 (s, 3H).

^{13}C NMR (151 MHz, $CDCl_3$): δ 83.2, 80.2, 69.4, 69.0, 56.6, 49.8, 48.1, 45.4, 41.3, 39.9, 37.0, 30.4, 29.8, 28.1, 28.0, 27.2, 26.3, 26.0, 22.0, 18.2, 17.4, 15.6, 15.5, 7.0, 4.9, −3.7, −4.8.

IR (Diamond-ATR, neat) ṽ (cm^{-1}): 3519, 2950, 2937, 1453, 1381, 1358, 1250, 1093, 1074, 998, 877, 841, 809.

HRMS (ESI): m/z: $[M+H]^+$ calc'd for $C_{33}H_{65}O_4Si_2$: 593.4416. Found: 593.4401.

Specific Rotation $[\alpha]^{26}_D$: −2.1 (*c* = 1.0, $CHCl_3$).

Synthesis of ketone (24)

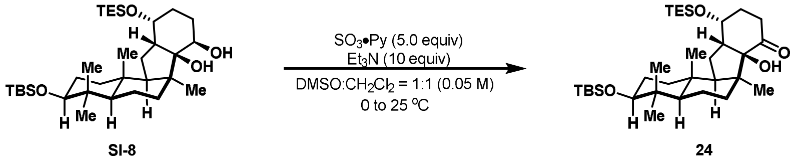

To a flame-dried reaction tube equipped with a stir-bar was charged with **SI-8** (60.0 mg, 0.10 mmol, 1.0 equiv), CH_2Cl_2 (1.0 mL, 0.10 M), and Et_3N (140 µL, 1.0 mmol, 10.0 equiv). The reaction vial was sealed with a screw cap (phenolic top with a polyvinyl-faced pulp liner) and placed in 0 °C ice-water bath. To the reaction mixture was added a solution of SO_3•Py (82 mg, 0.52 mmol, 5.0 equiv) in DMSO (1.0 mL, 0.5 M) over 1 min (final concentration: 0.05 M). The reaction mixture was removed from the ice-water bath and stirred at room temperature. After 17 h, the reaction mixture was diluted with H_2O (2 mL) and EtOAc (5 mL). The resulting mixture was transferred to a separatory funnel and the layers were separated. The aqueous layer was extracted with EtOAc (3 x 5 mL). The combined organic extracts were washed with brine (3 x 5 mL), dried over anhydrous Na_2SO_4, filtered, and concentrated *in vacuo* with the aid of a rotary evaporator. The residue was purified by flash column chromatography on silica gel (gradient elution: 1.4% to 2.5% EtOAc in hexanes) to yield **24** as a colorless solid (55 mg, 92%).

^{1}H NMR (600 MHz, $CDCl_3$): δ 4.08–4.05 (m, 1H), 3.38 (s, 1H), 3.18 (dd, *J* = 11.1, 4.0 Hz, 1H), 2.62 (dt, *J* = 17.7, 7.4 Hz, 1H), 2.47 (ddd, *J* = 7.4, 6.8, 6.8 Hz, 1H), 2.38 (dt, *J* = 17.6, 6.0 Hz, 1H), 2.13–2.06(m, 1H), 1.98 (dt, *J* = 14.3, 6.0 Hz, 1H), 1.92–1.78 (m, 3H), 1.60 (t, *J* = 12.9 Hz, 2H), 1.57–1.48 (m, 2H), 1.48–1.42 (m, 2H), 1.25 (s, 1H), 1.06 (dt, *J* = 14.5, 7.1 Hz, 1H), 1.00–0.95 (m, 15H), 0.88 (s, 12H), 0.83–0.79 (m, 1H), 0.81 (s, 3H), 0.64 (q, *J* = 7.9 Hz, 6H), 0.04 (s, 3H), 0.03 (s, 3H).

^{13}C NMR (151 MHz, $CDCl_3$): δ 216.0, 87.9, 80.1, 68.5, 62.8, 54.7, 50.3, 48.6, 41.3, 39.7, 36.8, 36.5, 30.9, 29.9, 28.8, 28.6, 28.1, 26.6, 26.0, 19.9, 18.3, 16.4, 16.1, 7.1, 5.0, −3.6, −4.8.

IR (Diamond-ATR, neat) ṽ (cm^{-1}): 3487, 2944, 2882, 2852, 2362, 2329, 1688, 1463, 1093, 1074, 1002, 838.

HRMS (ESI): m/z: $[M+H]^+$ calc'd for $C_{33}H_{63}O_4Si_2$: 591.4259. Found: 591.4252.

Specific Rotation $[\alpha]^{23}_D$: −46.4 (*c* = 1.0, $CHCl_3$).

Synthesis of dasyscyphin A (2)

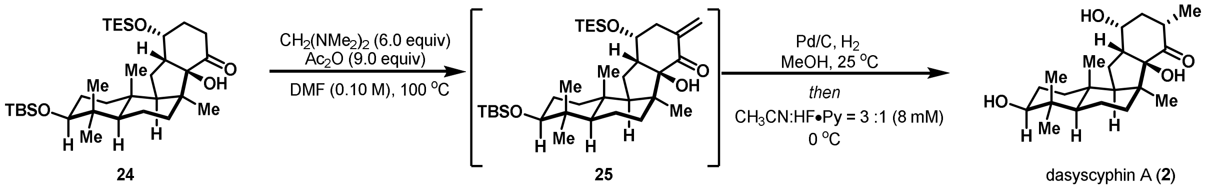

To a flame-dried reaction tube equipped with a stir-bar was charged with **24** (18 mg, 31 μmol, 1.0 equiv), DMF (3.1 mL, 10 mM), *N,N,N',N'*-tetramethyldiaminomethane (25 μL, 184 μmol, 6.0 equiv), and Ac_2O (26 μL, 277 μmol, 9.0 equiv). The reaction vial was sealed with a screw cap (phenolic top with a polyvinyl-faced pulp liner) and placed in preheated oil bath at 100 °C. After 2h, the reaction mixture was removed from the oil bath and allowed to cool to room temperature.

The reaction vessel was charged with MeOH (3.1 mL), and Pd/C (1.8 mg, 2 μmol, 6 mol%, 10% wt on activated charcoal) (final concentration: 5 mM). The reaction vial was sparged with H_2 by puncturing the septum with an exit needle and carefully submerging the needle connected to the H_2-filled balloon in the solvent of the reaction mixture until bubbles were observed. After 15 min of bubbling H_2 through the reaction mixture, the needle attached to the H_2-filled balloon was removed from the reaction solvent and placed in the headspace of the reaction vessel. Following this, the exit needle was removed from the septum. The reaction mixture was allowed to stir at room temperature. After 30 min, the H_2 balloon was removed from the reaction vessel. Using a needle connected to a dual manifold Schlenk line, a gentle stream of N_2 was directed into the flask, and the H_2 in the headspace was displaced with an exit needle. (Caution: ensure that H_2 is fully removed by carefully bubbling N_2 through the solution. This will reduce the risk of fire during the subsequent filtration). The reaction mixture was filtered over a packed pad of Celite (30 mL fritted funnel, 18 mm Celite powder) and washed with EtOAc (20 mL). The filtrate was washed with brine (3 x 10 mL) dried over Na_2SO_4, filtered, and concentrated *in vacuo* with the aid of a rotary evaporator give the crude ketone which was used without further purification.

To a 15 mL plastic scintillation vial equipped with a magnetic stir-bar was charged with above crude ketone and CH_3CN (3.0 mL, 10 mM). The reaction vessel was placed in a 0 °C ice-water bath. To the reaction mixture was added HF•Py (1.0 mL, 70% wt) (the final concentration was 7.5 mM). After 30 min, the reaction mixture transferred to a 50 mL plastic bottle and was diluted with sat. aq. $NaHCO_3$ (30 mL), transferred to a separatory funnel and the layers were separated. The aqueous layer was extracted with EtOAc (3 x 30 mL). The combined organic extracts were washed with brine (3 x 30 mL), dried over anhydrous Na_2SO_4, filtered, and concentrated *in vacuo* with the aid of a rotary evaporator. The residue was purified by flash column chromatography on silica gel (gradient elution: 50% to 60% EtOAc in hexanes) to yield **2** as a colorless solid (9.4 mg, 84% over two steps).

^{1}H NMR (600 MHz, $CDCl_3$ with 5% CD_3OD): δ 4.11 (dt, *J* = 10.6, 6.6 Hz, 1H), 3.17 (dd, *J* = 10.6, 5.5 Hz, 1H), 2.78 (dt, *J* = 11.5, 8.3 Hz, 1H), 2.36 (ddq, *J* = *J* = 11.3, 7.6, 7.3 Hz, 1H), 1.96 (ddd, *J* = 14.6, 3.8, 3.0 Hz, 1H),1.90–1.85 (m, 3H), 1.75 (dd, *J* = 14.2, 8.8 Hz, 1H), 1.71 (ddd, *J* = 13.1, 3.6, 3.6 Hz, 1H), 1.59–1.54 (m, 2H), 1.51 (dddd, *J* = 12.5, 12.5, 12.5, 4.7 Hz, 1H), 1.43 (ddt, *J* = 13.3, 5.6, 2.6 Hz, 1H), 1.36 (d, *J* = 8.3 Hz, 1H), 1.16–1.09 (m, 1H),1.12 (d, *J* = 7.1 Hz, 3H), 0.98 (s, 3H), 0.96–0.92 (m, 1H), 0.95

(s, 3H), 0.80 (s, 3H), 0.76 (s, 3H), 0.70 (dd, J = 11.9, 2.4 Hz, 1H).

^{13}C NMR (151 MHz, $CDCl_3$ with 5% CD_3OD): δ 219.2, 87.1, 79.1, 67.2, 61.9, 51.8, 51.7, 48.6, 41.9, 40.4, 38.4, 36.3, 34.8, 34.5, 30.2, 28.3, 27.1, 24.5, 20.4, 15.7, 15.5, 15.1.

IR (Diamond-ATR, neat) ṽ (cm^{-1}): 3384, 2933, 2851, 1681, 1467, 1340, 1168, 1181, 1081, 1032, 1016, 995.

HRMS (ESI): m/z: $[M+H]^+$ calc'd for $C_{22}H_{37}O_4$: 365.2686. Found: 365.2689.

Specific Rotation $[\alpha]^{26}_{D}$: −6.0 (c = 0.8, MeOH); Lit: $[\alpha]^{20}_{D}$: −6.0 (c = 0.8, MeOH).[5]

Comparison of natural and synthetic 2

Table SI-3: Spectroscopic Comparison of Dasyscyphin A (**2**) by ^{1}H NMR.

Carbon #	This Report: Dasyscyphin A ($CDCl_3$ containing 5% CD_3OD, 600 MHz)	Sterner & Co-workers: Dasyscyphin A ($CDCl_3$ containing 5% CD_3OD, 600 MHz)[5]	Absolute Difference
1α	0.96–0.92; m	0.94; m	-
1β	1.71; ddd; *J* = 13.1, 3.6, 3.6 Hz	1.69; ddd; *J* = 13.1, 3.8, 3.2 Hz	0.02
2α	1.59–1.54; m	1.54; m	–
2β	1.59–1.54; m	1.54; m	–
3	3.17; dd; *J* = 10.6, 5.5 Hz	3.15; dd; *J* = 9.8, 5.9 Hz	0.02
4	–	–	–
5	0.70; dd; *J* = 11.9, 2.4 Hz	0.69; dd; *J* = 11.7, 2.5 Hz	0.01
6α	1.43; ddt; *J* = 13.3, 5.6, 2.6 Hz	1.41; dddd; *J* = 13.3, 5.9, 3.0, 2.5 Hz	0.02
6β	1.51; dddd; *J* = 12.5, 12.5, 12.5, 4.7 Hz	1.50; dddd; *J* = 13.3, 12.2, 11.7, 4.4 Hz	0.01
7α	1.16-1.09; m	1.11; ddd; *J* = 14.7, 12.2, 5.9 Hz	–
7β	1.96; ddd; *J* = 14.7, 3.8, 3.0 Hz	1.94; ddd; *J* = 14.7, 4.4, 3.0 Hz	0.02
8	–	–	–
9	1.36; d; *J* = 8.3 Hz	1.34; d; *J* = 8.1 Hz	0.02
11α	1.71; ddd; *J* = 13.1, 3.6, 3.6 Hz	1.72; dd; *J* = 14.2, 8.8 Hz	0.01
11β	1.90–1.80; m	1.82; ddd; *J* = 14.2, 12.4, 8.1 Hz	–
12	2.78; dt; *J* = 11.5, 8.3 Hz	2.76; ddd; *J* = 12.4, 8.8, 6.4 Hz	0.02
13	4.11 dt; *J* = 10.6, 6.6 Hz	4.09; ddd; *J* = 8.2, 7.6, 6.4 Hz	0.02
14α	1.90–1.80; m	1.82; m	–
14β	1.90–1.80; m	1.82; m	–
15	2.36; ddq; *J* = 11.3, 7.6, 7.3 Hz	2.37; ddq; *J* = 10.3, 8.7, 7.1 Hz	0.01
16	–	–	–
17	–	–	–
18	0.80; s	0.79; s	0.01
19	0.95; s	0.93; s	0.02
20	0.76; s	0.74; s	0.02
21	0.98; s	0.96; s	0.02
22	1.12; d; *J* = 7.1 Hz	1.10; d; *J* = 7.1 Hz	0.02

The ^{1}H-NMR data for synthetic dasyscyphine A listed in the table above are reported in ppm relative to $CDCl_3$ calibrated to 7.26 ppm.

Table SI-4: Spectroscopic Comparison of Dasyscyphin A (**2**) by ^{13}C NMR.

Carbon #	This Report: Dasyscyphin A ($CDCl_3$ containing 5% CD_3OD, 151 MHz)	Sterner & Co-workers: Dasyscyphin A ($CDCl_3$ containing 5% CD_3OD, 125 MHz)[5]	Absolute Difference
1	40.4	40.6	0.2
2	27.1	27.1	0.0
3	79.1	79.1	0.0
4	38.4	38.5	0.1
5	51.8	51.8	0.0
6	20.4	20.5	0.1
7	34.8	34.7	0.1
8	48.6	48.6	0.0
9	61.9	61.9	0.0
10	36.3	36.5	0.2
11	24.5	24.5	0.0
12	51.7	51.8	0.1
13	67.2	67.1	0.1
14	34.5	34.7	0.2
15	41.9	42.0	0.1
16	219.2	219.3	0.1
17	87.1	87.2	0.1
18	30.2	30.2	0.0
19	28.3	28.4	0.1
20	15.5	15.6	0.1
21	15.7	15.8	0.1
22	15.1	15.2	0.1

The ^{13}C-NMR data for synthetic dasyscyphin A listed in the table above are reported in ppm relative to $CDCl_3$ calibrated to 77.0 ppm

5. General procedure for the optimization of polyolefin cyclization

To a flame-dried reaction tube equipped with a magnetic stir-bar was charged with Cp_2TiCl_2 (2.5 mg, 10 μmol, 10 mol%), activated Zn (39 mg, 0.6 mmol, 6.0 equiv), and 2,4,6-collidine hydrochloride (32 mg, 0.2 mmol, 2.0 equiv). The reaction vessel was transferred to a N_2-filled glovebox, and THF (1 mL, 0.10 M) was added. The solution was allowed to stir for 10 min, resulting in a green heterogeneous mixture. To the reaction mixture was added **5** (33 mg, 0.1 mmol, 1.0 equiv) dropwise over 1 min. The reaction vessel was sealed with a rubber septum, removed from the glovebox, and stirred at room temperature.

After 18 h, the reaction mixture was diluted with aq. KH_2PO_4 solution (10% w/v, 2 mL), and the resulting solution was stirred at room temperature. After 30 min, the reaction mixture was filtered over a packed pad of Celite (15 mL fritted funnel, 18 mm Celite powder) and washed with EtOAc (10 mL). The filtrate was transferred to a separatory funnel and the layers were separated. The aqueous layer was extracted with EtOAc (3 x 5 mL). The combined organic extracts were washed with sat. aq. HCl (10 mL, 1.0 M), brine (10 mL), dried over anhydrous Na_2SO_4, filtered, and concentrated *in vacuo* with the aid of a rotary evaporator. The yield of **14** was determined by ^1H NMR spectroscopy of the crude mixture utilizing CH_2Br_2 as the internal standard.

Scheme SI-5. Optimization of Polyolefin Cyclization

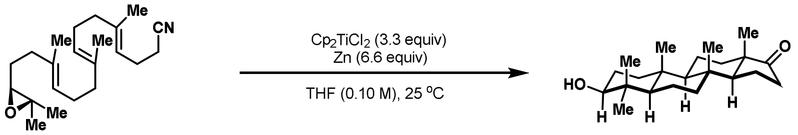

5 14

entry	variation from standard conditions	yield (%)[a]
1	none [*ref.* 15]	0
2	60 °C	28
3	2,4,6-collidine·HCl (2.0 equiv)	39
	catalytic polyolefin cyclization with 10 mol% Cp_2TiCl_2	
4	2,4,6-collidine·HCl (2.0 equiv)	42
5	2,4,6-collidine·TFA (2.0 equiv)	22
6	2,4,6-collidine·TfOH (2.0 equiv)	8
7	2,4,6-collidine·HCl (2.0 equiv), Mn (6.0 equiv)	57

[a]Reactions performed on a 0.10 mmol scale and the yields was determined by ^1H NMR spectroscopy of the crude reaction mixtures utilizing CH_2Br_2 as the internal standard.

6. Additional attempts toward E-ring construction of habiterpenol

Scheme SI-6. Prins Cyclization at Lower Temperature

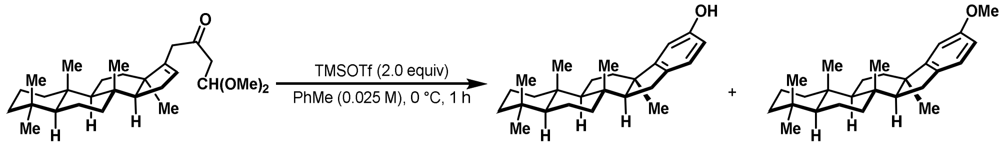

16 — habiterpenol (**1**) N.D. — **17** 10%

Scheme SI-7. Attempted the Cyclization with Enone

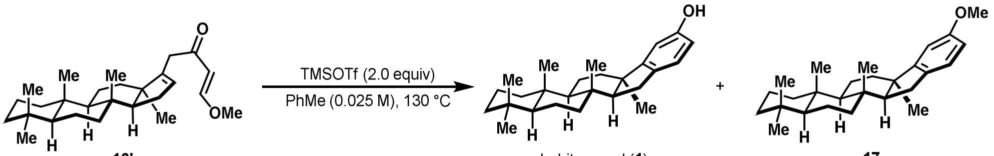

16' — habiterpenol (**1**) N.D. complex mixture — **17** N.D.

Scheme SI-8. Investigation of the Methyl Ether Formation

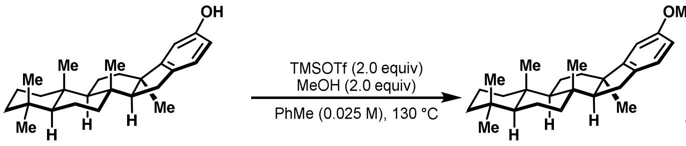

habiterpenol (**1**) — **17** N.D. 10 % **1** recovery

-Resubjection of **1** to the reaction conditions did not produce **17**. This indicated that the formation of the ethereal linkage likely occurs prior to the formation of the phenol substructure.

7. Additional attempts toward the D-ring construction of dasyscyphin A

Scheme SI-9. Initial Investigation of the SmI_2-Mediated Cyclization

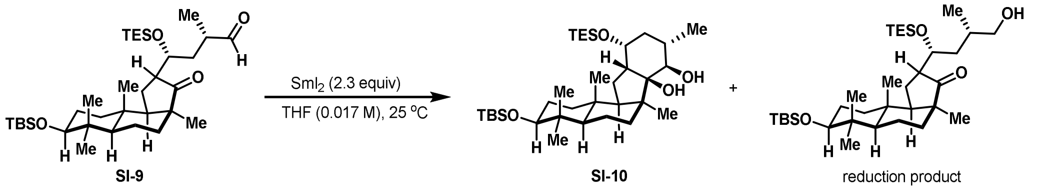

Entry	Variation from above	Yield of **SI-10**[a]
1	-	trace product
2	HMPA (8.0 equiv), −78 °C	N.D.
3	*t*-BuOH (8.0 equiv), −78 °C	reduction product
4	MeOH (5.0 equiv), −78 °C	reduction product
5	LiBr (8.0 equiv), −78 °C	reduction product

[a]Reactions performed on a 8.4 μmol scale and the yields was determined by ^{1}H NMR spectroscopy of the crude reaction mixtures utilizing CH_2Br_2 as the internal standard.

Scheme SI-10. Mechanistic Probe for the SmI_2-Mediated Cyclization

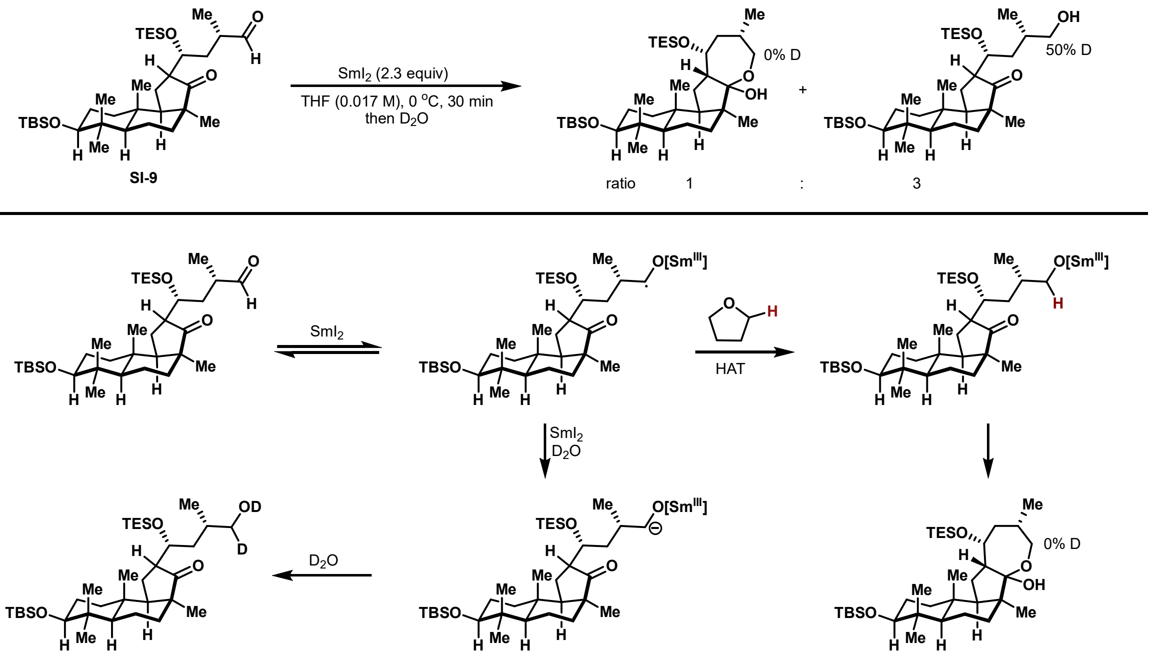

Scheme SI-11. Optimization of the SmI_2-Mediated Cyclization

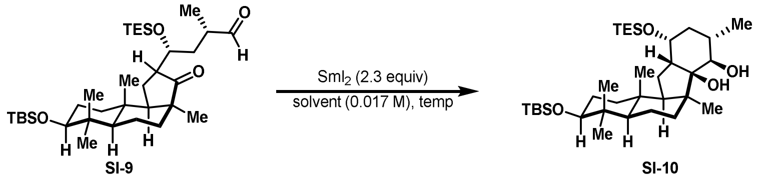

Entry	Solvent	Temp.	Additive	Yield of **SI-10**[a]
1	2-MeTHF	0 °C	-	<5%
2	CH_3CN	0 °C	-	<5%
3	PhMe	0 °C	-	8%
4	Heptane	0 °C	-	<5%
5	$PhCF_3$	0 °C	-	<5%
6	PhMe	−78 °C, −40 °C or −20 °C	-	N.D. (only SM)
7	PhMe	−78 °C	HMPA (10.0 equiv)	N.D. (reduction)
8	PhMe	0 °C	TBAB (8.0 equiv)	39%
9	PhMe	0 °C	LiCl (8.0 equiv)	N.D.
10	PhMe	−78 °C to r.t.	-	39%
11	PhMe	−78 °C to r.t.	TBAB (8.0 equiv)	49%
12	PhMe	−78 °C to r.t.	$ZnBr_2$ (8.0 equiv)	N.D.
13	PhMe	−78 °C to r.t.	$MgBr_2$•OEt_2 (2.0 equiv)	25%
14	PhMe	−78 °C to r.t.	LiBr (8.0 equiv)	25%

[a]Reactions performed on a 8.4 μmol scale and the yields was determined by ^1H NMR spectroscopy of the crude reaction mixtures utilizing CH_2Br_2 as the internal standard.

Scheme SI-12. Optimization of the SmI_2-Mediated Cyclization on **23**

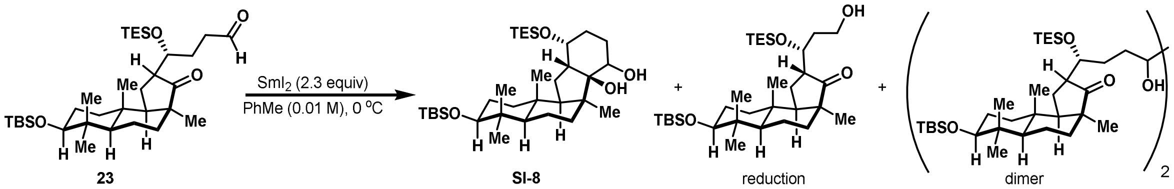

Entry	Additive	Yield of **SI-8**[a]	Yield of **reduction**[a]	Yield of **dimer**[a]
1[b]	TBAB (8.0 equiv)	33%	10%	27%
2[c]	TBAB (8.0 equiv)	32%	29%	10%
3[c]	LiBr (8.0 equiv)	45%	35%	<5%
4[c]	LiBr (5.0 equiv)	47%	6%	12%
5[c]	LiBr (3.0 equiv)	57%	N.D.	17%

[a]Reactions performed on a 8.4 μmol scale and the yields was determined by ^1H NMR spectroscopy of the crude reaction mixtures utilizing CH_2Br_2 as the internal standard. [b]SmI_2 (0.10 M in THF) solution was added dropwise into the aldehyde **23** and additive soulution in PhMe (0.01 M). [c]Aldehyde **23** soulution in PhMe (0.02 M) was added into the mixture of SmI_2 (0.10 M in THF) and additive in PhMe (0.02 M).

Scheme SI-13. Unsuccessful Attempts toward the D-Ring Formation

Reductive cyclization onto nitrile group

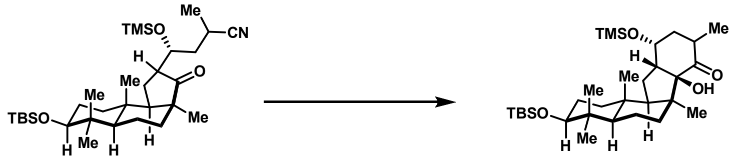

Entry	Conditions	Result
1	SmI_2 (2.2 equiv), THF, 25 °C	only SM
2	SmI_2 (2.2 equiv), THF, 40 °C or 390 nm LEDs	only SM
3	SmI_2 (2.2 equiv), *t*-BuOH (8 equiv), THF, 25 °C	ketone reduction
4	SmI_2 (2.2 equiv), HMPA (11 equiv), THF, 25 °C	unknown product
5	Cp_2TiPh (2.2 equiv), THF, 25 °C	most SM, trace ketone reduction
6	Cp_2TiCl_2 (10 mol%), TMSCl (2.0 equiv), Collidine•HCl (2.0 equiv), Zn (3.0 equiv), THF, 40 °C	only SM

Cyclization of ketyl anion or ketyl radical onto alkyne group

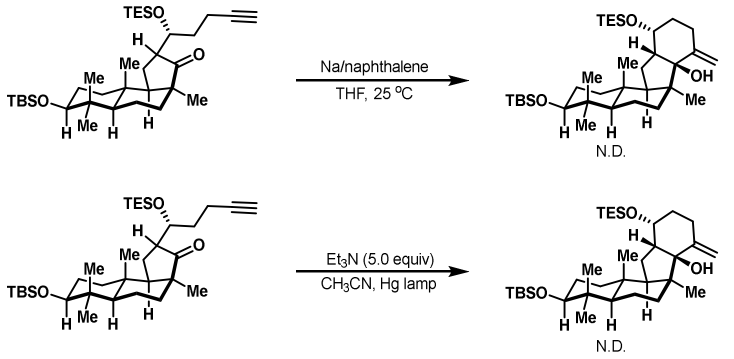

Cyclometalation of ketone and alkyne

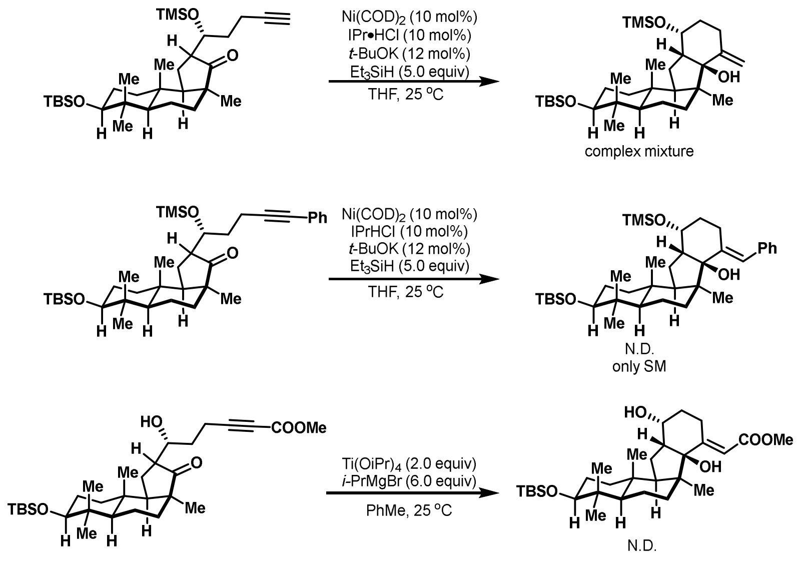

NHC-catalyzed intramolecular benzoin condensation

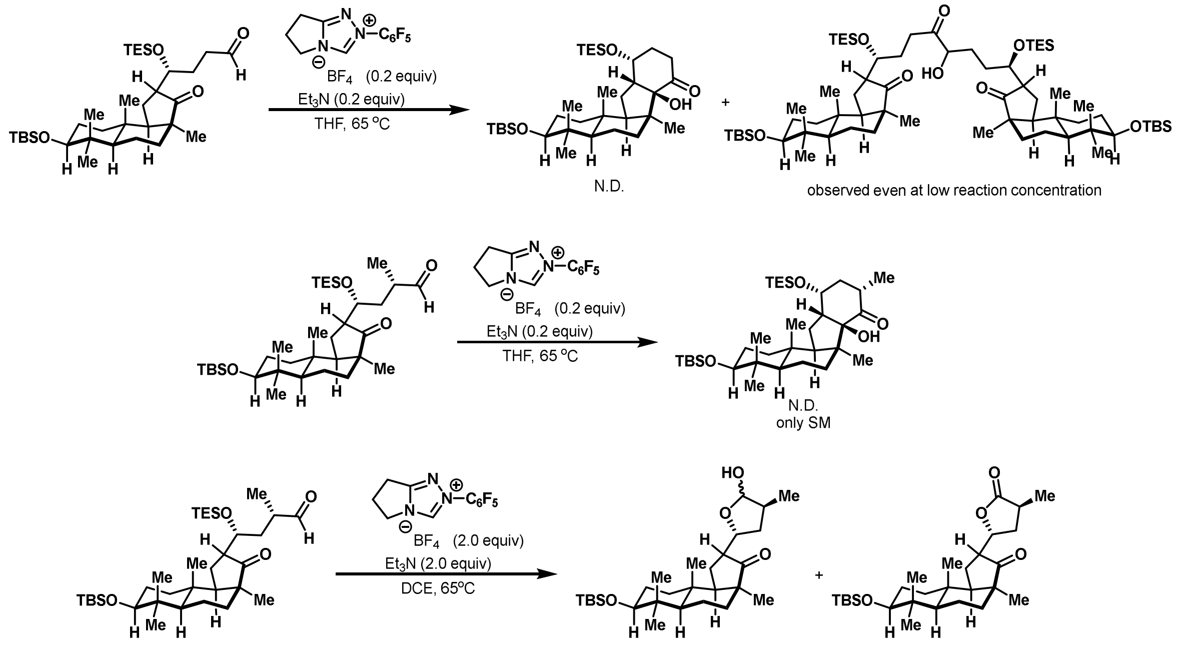

Ti^III mediated reductive cyclization

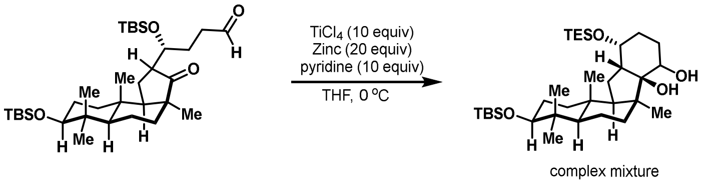

Intramolecular vinyl-Cerium reagent addition

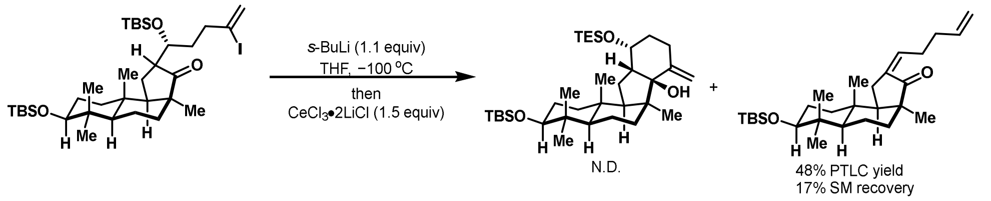

Scheme SI-14. Attempts at the Diol Oxidation

Attempted to the diol oxidation

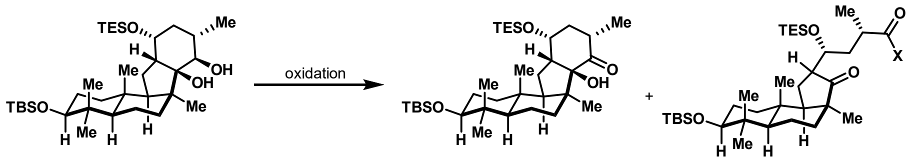

SI-10 → SI-11

Entry	Conditions	Results
1	DMP (2.0 equiv), CH_2Cl_2	SM
2	DMP (2.0 equiv), CH_2Cl_2:DMF = 10:1	SM
3	PIFA (1.5 equiv), HFIP	complex mixture
4	IBX (2.0 equiv), DMSO	complex mixture
5	NBS, H_2O:acetone:AcOH = 10:1:1	complex mixture
6	PDC (2.0 equiv), Celite, CH_2Cl_2	ring-opening to aldehyde
7	TPAP (0.3 equiv), NMO (2.0 equiv)	ring-opening to acid
8	$(COCl)_2$ (1.5 equiv), DMSO (1.5 equiv), Et_3N (3.0 equiv), CH_2Cl_2	SM
9	NCS (1.5 equiv), DMS (1.5 equiv), PhMe	SM
10	SO_3Py (5.0 equiv), Et_3N(10 equiv), DMSO	SM
11	9-Azabicyclo[3.3.1]nonane *N*-oxyl (0.2 equiv), $NaNO_3$, O_2	desilylation

---

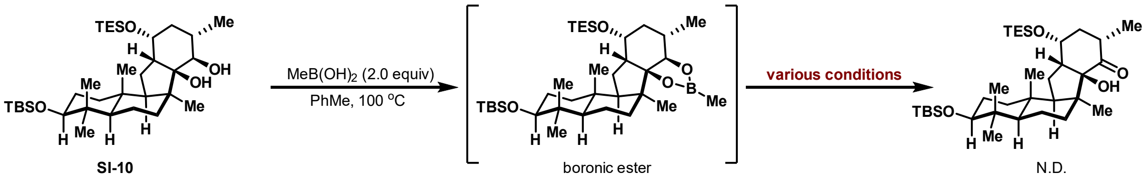

SI-10 → boronic ester → N.D.

8. Additional mechanistic probe for quaternary center epimerization

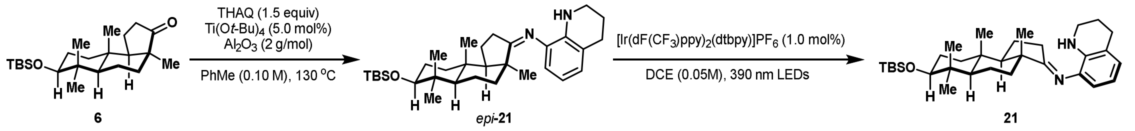

Inside a N_2-filled glovebox, a flame-dried reaction tube equipped with a magnetic stir-bar was charged with **6** (50.0 mg, 132 µmol, 1.0 equiv), Al_2O_3 (264 mg, 2.0 g/mmol), and PhMe (1.3 mL, 0.10 M). To the stirred reaction mixture were added 1,2,3,4-tetrahydroquinolin-8-amine (29 mg, 198 µmol, 1.5 equiv) and Ti(O*t*-Bu)$_4$ (2.6 µL, 6.6 µmol, 5.0 mol%). The reaction tube was sealed with a rubber septum, removed from the glovebox, fitted with a N_2-filled balloon. The reaction vessel was placed in a preheated oil bath at 130 °C. After 48 h, the reaction mixture was removed from the oil bath and allowed to cool to room temperature. Once at room temperature, the reaction mixture was filtered over a short plug of SiO_2 (15 mL fritted funnel, 3 cm SiO_2, treated with 5% Et_3N in hexanes) and the reaction flask was rinsed with hexanes (3 x 1 mL). The SiO_2 plug was eluted with 10% EtOAc in hexanes (50 mL) and the filtrate was concentrated *in vacuo* with the aid of a rotary evaporator. The residue was transferred to a separate flame-dried 50 mL flask using CH_2Cl_2 (20 mL) and concentrated *in vacuo* with the aid of a rotary evaporator. The crude mixture was successively azeotroped with benzene (15 mL) and CH_2Cl_2 (3 x 5 mL), then dried under high vacuum utilizing a dual-manifold Schlenk line to yield *epi*-**21** as a pale-yellow solid (58 mg, 86%), which was used without further purification.

Inside a N_2-filled glovebox, a flame-dried reaction tube equipped with a magnetic stir-bar was charged with *epi*-**21** (15 mg, 29.5 µmol, 1.0 equiv), [Ir(dF(CF$_3$)ppy)$_2$(dtbpy)]PF$_6$ (0.3 mg, 0.29 µmol, 1.0 mol%), and 1,2-dichloroethane (0.6 mL, 0.05 M). The reaction tube was sealed with a septum cap and removed from the glovebox. The reaction vessel was subjected to LED irradiation utilizing one 30 W 390 nm LED at 50% intensity with distances of 10 cm for 5 h. The reaction mixture was diluted with CH_2Cl_2 (3 x 2 mL), transferred to a 25 ml round-bottom flask, and concentrated *in vacuo* with the aid of a rotary evaporator. The yield was determined by ^{1}H NMR spectroscopy of the crude reaction mixture using 1,1,2,2-tetrachlotoethane (3.8 mg, 23 µmol) as the internal standard (*epi*-**21**, 100% and **21**, 0%).

^{1}H NMR of crude reaction mixture (400 MHz, $CDCl_3$)
TCE (3.8 mg) used as the internal standard

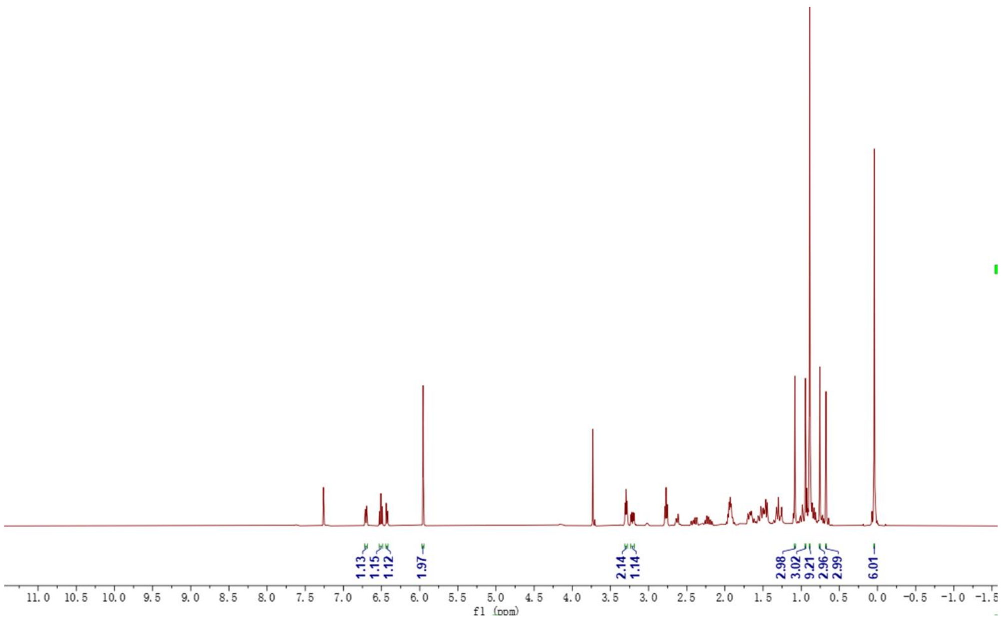

^{1}H NMR of crude reaction mixture (400 MHz, $CDCl_3$)
after 390nm irradiation

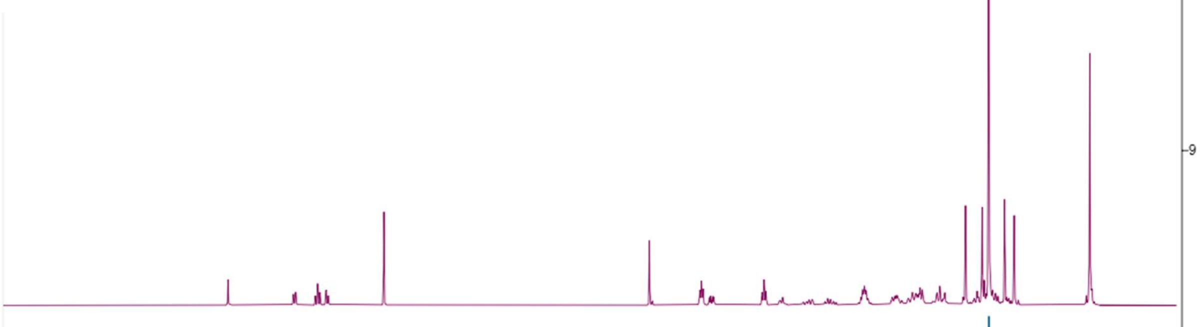

^{1}H NMR of *epi*-**21** (400 MHz, $CDCl_3$)

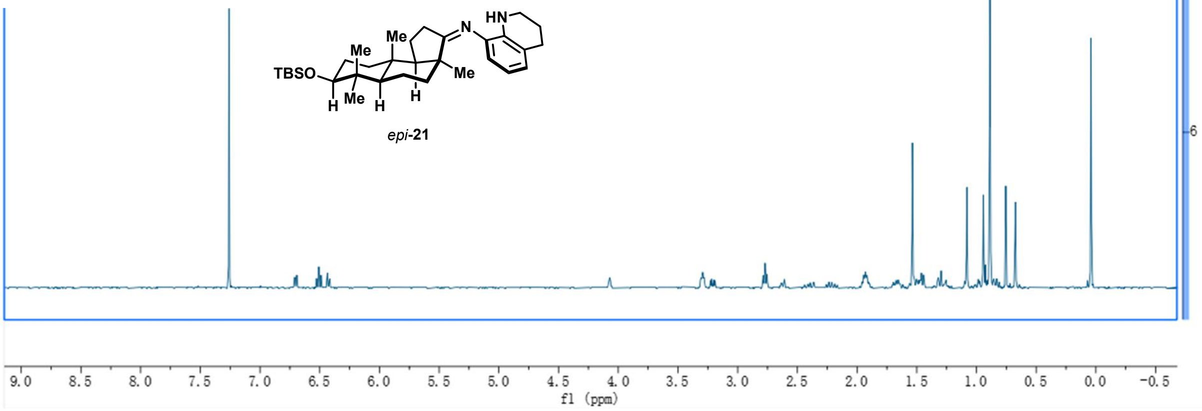

Scheme SI-15. Energy Profiles of Ketones and Imines

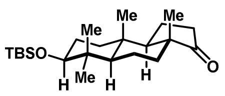

7
ΔG = 0 kcal/mol

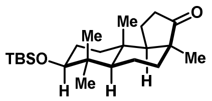

6
ΔG = −2.7 kcal/mol

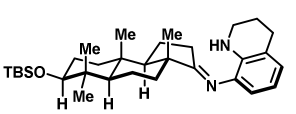

21
ΔG = 0 kcal/mol

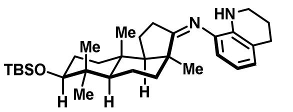

epi-**21**
ΔG = −2.4 kcal/mol

9. Computational details

Density functional theory (DFT) calculations were performed with Gaussian 16.[6] Geometry optimizations and frequency calculations were performed using the M06-2X functional and def2-SVP basis set, and single-point energy calculations were performed with the def2-TZVP[7,8] basis set. Frequency calculations confirmed the optimized structures as minima (zero imaginary frequencies) on the potential energy surface. Initial structures were made using GaussView.[9] Triplet transition state free energy barriers were calculated by subtracting the Gibbs free energies of reactants from the Gibbs free energy of the triplet transition state. These free energies were obtained using single point electronic energies with thermal corrections at the geometry optimization level of theory using Shermo.[10]

Coordinates

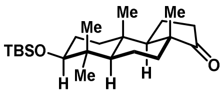

7

Charge = 0 Multiplicity = 1

C	-0.49588	1.43437	0.6645
C	-1.02939	0.2245	-0.09409
C	-0.20365	-1.06262	0.14524
C	1.27985	-0.72836	-0.17628
C	1.87826	0.503	0.57917
C	0.96449	1.71551	0.3248
C	2.22666	-1.93986	-0.14464
C	3.59415	-1.63044	-0.77072
C	4.22762	-0.42267	-0.09069
C	3.23711	0.75824	-0.09948
C	5.39767	0.21464	-0.83628
C	5.39005	1.7204	-0.53983
C	4.09488	1.98196	0.25289
O	6.18722	-0.35886	-1.53491
C	4.82202	-0.81424	1.27778
C	2.03403	0.30518	2.1008
C	-0.44467	-1.59509	1.56368
C	-0.72211	-2.12128	-0.83925
O	-2.37663	-0.00311	0.25047
Si	-3.69993	0.46193	-0.6749
C	-5.1906	-0.09912	0.34462
C	-3.64009	-0.38552	-2.35268
C	-3.67319	2.32392	-0.93612
C	-5.04767	-1.59759	0.64378
C	-6.48806	0.15366	-0.43307
C	-5.21454	0.67999	1.66644
H	-1.11982	2.30641	0.4162
H	-0.63326	1.2579	1.7426
H	-0.94385	0.44733	-1.18046
H	1.25527	-0.40323	-1.23582
H	1.03698	1.99301	-0.74222
H	1.32772	2.58243	0.90037
H	2.3602	-2.29111	0.89181
H	1.7728	-2.77814	-0.69058
H	4.26239	-2.50277	-0.71099
H	3.46139	-1.40771	-1.84258
H	2.99046	0.88873	-1.17268
H	5.40355	2.24794	-1.50542
H	6.31463	1.99971	-0.01484
H	4.29663	2.03623	1.33317
H	3.61289	2.92579	-0.03536
H	4.10865	-1.37535	1.89193
H	5.69499	-1.46034	1.10231
H	5.15846	0.0563	1.85936

H	1.15128	0.66351	2.64528
H	2.18091	-0.74403	2.38502
H	2.89289	0.87658	2.48064
H	-0.28807	-0.8402	2.34263
H	-1.48406	-1.94028	1.64777
H	0.21662	-2.44853	1.77625
H	-0.31176	-3.11733	-0.623
H	-1.81601	-2.19022	-0.7559
H	-0.46771	-1.86133	-1.8798
H	-2.68568	-0.1815	-2.86211
H	-4.44661	-0.0131	-3.00262
H	-3.74666	-1.47658	-2.26521
H	-4.56205	2.65735	-1.49326
H	-3.6442	2.86454	0.02127
H	-2.78817	2.61943	-1.52126
H	-4.11705	-1.80442	1.19307
H	-5.89436	-1.94656	1.25945
H	-5.03975	-2.20036	-0.27818
H	-6.62129	1.22047	-0.67402
H	-7.3601	-0.15968	0.16602
H	-6.51554	-0.41346	-1.37686
H	-4.27209	0.55111	2.22028
H	-5.36982	1.75767	1.50141
H	-6.03837	0.32102	2.30691

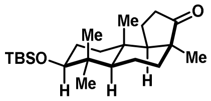

6

Charge = 0 Multiplicity = 1

C	-0.45676	-1.52731	-0.31422
C	-1.01743	-0.20881	0.20217
C	-0.15139	1.00997	-0.18727
C	1.32335	0.73175	0.23996
C	1.93543	-0.64265	-0.15985
C	0.94691	-1.7567	0.23133
C	2.29353	1.86141	-0.11913
C	3.57344	1.74609	0.70001
C	4.20698	0.35808	0.67084
C	3.23406	-0.84343	0.68165
C	5.04271	0.09814	-0.58302
C	5.13804	-1.41277	-0.78805
C	4.11812	-2.0215	0.19093
O	5.54804	0.93866	-1.27651
C	5.20825	0.2447	1.83885
C	2.22966	-0.74336	-1.66692
C	-0.34706	1.33677	-1.67664
C	-0.67776	2.213	0.61191
O	-2.33561	-0.0198	-0.25935
Si	-3.71944	-0.316	0.64639
C	-5.13741	0.05177	-0.54923
C	-3.75682	0.81561	2.14824
C	-3.7277	-2.1017	1.23604
C	-4.95733	1.4709	-1.10515
C	-6.48348	-0.05474	0.17785
C	-5.08847	-0.95425	-1.70694
H	-1.12755	-2.34147	-0.00031
H	-0.46922	-1.52177	-1.41521
H	-1.00382	-0.26036	1.3134
H	1.28054	0.70017	1.34783
H	0.88884	-1.79931	1.33336
H	1.33074	-2.73509	-0.09816
H	2.52967	1.84109	-1.19383
H	1.83696	2.84202	0.07492
H	4.32814	2.4686	0.35214
H	3.33994	1.99655	1.74857
H	2.90521	-1.02556	1.71822
H	4.93012	-1.63292	-1.84387
H	6.17398	-1.73183	-0.59942
H	4.63178	-2.47815	1.04765
H	3.52096	-2.81937	-0.26993
H	5.91509	1.08648	1.80592
H	4.66653	0.2804	2.79576
H	5.79059	-0.6875	1.8108
H	1.33129	-0.56452	-2.26609

H	2.98776	-0.02481	-2.00718
H	2.58731	-1.75187	-1.92066
H	-0.32354	0.45123	-2.32337
H	-1.3324	1.8002	-1.81884
H	0.41703	2.04426	-2.02971
H	-0.22408	3.15694	0.27942
H	-1.76374	2.29961	0.46431
H	-0.48144	2.09647	1.68993
H	-2.83523	0.71027	2.74124
H	-4.60281	0.56056	2.80463
H	-3.85162	1.8733	1.86215
H	-4.64958	-2.32836	1.79324
H	-3.65309	-2.80549	0.39416
H	-2.87964	-2.28968	1.91319
H	-3.99095	1.57365	-1.62094
H	-5.75875	1.70248	-1.82751
H	-5.00105	2.23098	-0.30908
H	-6.64419	-1.05989	0.59956
H	-7.31297	0.1449	-0.52191
H	-6.56253	0.67457	0.99943
H	-4.11095	-0.93013	-2.21237
H	-5.26915	-1.98368	-1.36008
H	-5.86474	-0.7155	-2.45402

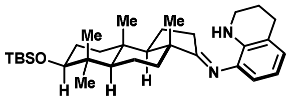

21

Charge = 0 Multiplicity = 1

C	2.81579	-1.15805	-1.25058
C	3.09256	-0.0761	-0.21309
C	2.39455	-0.33382	1.14403
C	0.88294	-0.55123	0.85356
C	0.54427	-1.65481	-0.19946
C	1.321	-1.34253	-1.49167
C	0.00099	-0.6672	2.10752
C	-1.495	-0.55713	1.78054
C	-1.90041	-1.59651	0.74201
C	-0.96146	-1.48519	-0.4796
C	-3.22462	-1.34622	0.04315
C	-3.16348	-2.01176	-1.33109
C	-1.66198	-2.29934	-1.57364
C	-2.01757	-2.99268	1.38527
C	0.88217	-3.0904	0.25197
C	3.07598	-1.49269	1.88316
C	2.58168	0.9301	1.99622
O	4.48249	0.05577	-0.01824
Si	5.43963	1.25063	-0.71019
C	7.19433	0.80677	-0.16169
C	4.91106	2.94106	-0.07804
C	5.24866	1.22011	-2.58084
C	7.21873	0.69077	1.36842
C	8.18041	1.88964	-0.61671
C	7.58238	-0.54426	-0.77703
H	3.32207	-0.88111	-2.18785
H	3.28913	-2.09296	-0.91253
H	2.66786	0.87582	-0.60153
H	0.56886	0.39083	0.36091
H	0.91202	-0.41561	-1.93231
H	1.15151	-2.14286	-2.23031
H	0.20333	-1.61734	2.62884
H	0.26343	0.12793	2.81895
H	-2.10674	-0.66052	2.68983
H	-1.70565	0.44476	1.36981
H	-1.06938	-0.42894	-0.79635
H	-3.60528	-1.33727	-2.08086
H	-3.76273	-2.93297	-1.35337
H	-1.44981	-3.37436	-1.47511
H	-1.34406	-1.99958	-2.58132
H	-1.14386	-3.24502	1.99721
H	-2.89987	-2.99657	2.04249
H	-2.14944	-3.79049	0.63979
H	1.88934	-3.38609	-0.06812
H	0.84188	-3.21914	1.34049
H	0.18436	-3.81314	-0.19383

H	3.17514	-2.39724	1.27237
H	4.08828	-1.18654	2.18009
H	2.51724	-1.75424	2.79435
H	2.27036	0.7707	3.03786
H	3.64554	1.20668	2.00832
H	2.00589	1.77758	1.58959
H	3.83729	3.10573	-0.25725
H	5.45972	3.7397	-0.60024
H	5.09087	3.05019	1.00145
H	5.9021	1.9682	-3.05532
H	5.49779	0.23288	-2.99672
H	4.21258	1.45909	-2.86805
H	6.50979	-0.0744	1.71827
H	8.22787	0.40781	1.71389
H	6.95839	1.64381	1.85548
H	8.184	2.00759	-1.71219
H	9.20709	1.62487	-0.31104
H	7.9458	2.86907	-0.17081
H	6.85847	-1.32705	-0.50353
H	7.63151	-0.49243	-1.87593
H	8.57675	-0.85784	-0.41553
N	-4.12544	-0.61198	0.5516
C	-5.34671	-0.32912	-0.08428
C	-5.80958	1.00694	0.04672
C	-6.14056	-1.27314	-0.73733
C	-7.04683	1.37316	-0.51795
C	-7.36907	-0.91194	-1.28973
H	-5.80301	-2.30879	-0.77996
C	-7.8025	0.40694	-1.18461
H	-7.98429	-1.65806	-1.79307
H	-8.76082	0.70209	-1.61938
N	-5.00915	1.92548	0.69186
H	-4.29014	1.49747	1.26647
C	-5.5655	3.19126	1.10874
H	-6.19399	3.09308	2.01732
H	-4.74075	3.87505	1.35514
C	-6.41239	3.76076	-0.02381
H	-5.76517	3.90271	-0.90342
H	-6.81261	4.74442	0.25943
C	-7.54621	2.79432	-0.36261
H	-8.0601	3.10849	-1.28347
H	-8.30296	2.82818	0.44006

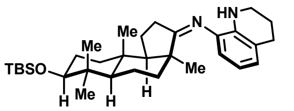

epi-21

Charge = 0 Multiplicity = 1

C	-2.52925	0.11589	1.53798
C	-3.17641	0.35858	0.18076
C	-2.25728	-0.01401	-1.00409
C	-0.88242	0.69764	-0.81076
C	-0.20315	0.57029	0.58337
C	-1.24084	0.91869	1.66712
C	0.13211	0.39897	-1.91893
C	1.23534	1.44887	-1.92462
C	1.8926	1.66062	-0.56271
C	0.93359	1.63827	0.6554
C	2.91681	0.60392	-0.18868
C	3.05803	0.57436	1.32167
C	1.89778	1.44144	1.85085
C	2.67463	2.98989	-0.59766
C	0.33784	-0.84476	0.85369
C	-2.18434	-1.54267	-1.15121
C	-2.9201	0.5312	-2.27941
O	-4.389	-0.35391	0.08243
Si	-5.91464	0.31346	0.30166
C	-7.08479	-1.16726	0.17777
C	-6.25317	1.59083	-1.03646
C	-6.00913	1.159	1.97896
C	-6.82463	-1.89077	-1.15045
C	-8.54297	-0.69603	0.23653
C	-6.79913	-2.12736	1.34013
H	-3.24256	0.40723	2.32398
H	-2.35058	-0.96295	1.66605
H	-3.36592	1.45183	0.0982
H	-1.12544	1.77638	-0.89795
H	-1.48925	1.99129	1.58019
H	-0.80248	0.78058	2.6681
H	0.57021	-0.60208	-1.78631
H	-0.35739	0.39317	-2.90294
H	2.02455	1.1834	-2.64409
H	0.80111	2.41003	-2.24892
H	0.43006	2.61672	0.72675
H	3.02413	-0.46808	1.67014
H	4.05457	0.9588	1.59059
H	2.27213	2.41778	2.18778
H	1.39909	0.98753	2.71771
H	3.3281	3.00514	-1.4832
H	1.97817	3.83901	-0.66813
H	3.30572	3.13329	0.29165
H	-0.45357	-1.59773	0.78716
H	1.12879	-1.14334	0.15269

H	0.75243	-0.90753	1.87013
H	-2.0633	-2.06627	-0.19513
H	-3.1213	-1.90986	-1.59097
H	-1.35766	-1.83598	-1.81434
H	-2.42079	0.16854	-3.18856
H	-3.96475	0.19087	-2.31919
H	-2.91189	1.633	-2.29731
H	-5.45694	2.35092	-1.05931
H	-7.20355	2.11211	-0.8452
H	-6.30765	1.13323	-2.03509
H	-7.01287	1.57589	2.15346
H	-5.77955	0.46156	2.79777
H	-5.29252	1.99371	2.03207
H	-5.7789	-2.22565	-1.21957
H	-7.47612	-2.77732	-1.23628
H	-7.03278	-1.24317	-2.0168
H	-8.76506	-0.16441	1.17571
H	-9.2269	-1.56	0.17783
H	-8.78865	-0.02344	-0.60041
H	-5.74361	-2.43949	1.34629
H	-7.02541	-1.66664	2.31442
H	-7.42234	-3.03356	1.24971
N	3.53889	-0.09202	-1.04851
C	4.51782	-1.00621	-0.59786
C	4.2553	-2.37378	-0.59466
C	5.78849	-0.52678	-0.2019
C	5.22259	-3.27903	-0.15699
H	3.27634	-2.71291	-0.93768
C	6.76627	-1.44109	0.22893
C	6.46383	-2.80594	0.25378
H	5.00559	-4.3475	-0.1424
H	7.2297	-3.50475	0.59861
C	8.13247	-0.94022	0.64843
H	8.85088	-1.11644	-0.17054
H	8.49273	-1.52807	1.50583
C	7.39207	1.31566	-0.11436
H	7.94109	1.17718	-1.06769
H	7.38146	2.39274	0.10539
C	8.11196	0.54856	0.98667
H	7.57523	0.71242	1.93454
H	9.1338	0.93353	1.11103
N	6.02306	0.84936	-0.20223
H	5.4357	1.35025	-0.86115

10. References

1. Zaragoza, F.; Stephensen, H. (Cyanomethyl)trialkylphosphonium Iodides: Efficient Reagents for the Intermolecular Alkylation of Amines with Alcohols in Solution and on Solid Phase. *J. Org. Chem.* **2001**, *66*, 2518–2521.

2. Corey, E. J.; Noe, M. C.; Lin, S. A Mechanistically Designed *Bis*-cinchona Alkaloid Ligand Allows Position- and Enantioselective Dihydroxylation of Farnesol and Other Oligoprenyl Derivatives at the Terminal Isopropylidene Unit. *Tetrahedron Lett.* **1995**, *36*, 8741–8744.

3. Uchida, R.; Yokota, S.; Tomoda, H. Structure Elucidation of Meroterpenoid Habiterpenol, a Novel Abrogator of Bleomycin-Induced G2 Arrest in Jurkat Cells, Produced by *Phytohabitans suffuscus* 3787_5. *J. Antibiot.* **2014**, *67*, 783–786.

4. Chen, X.; Zhang, D.; Xu, D.; Zhou, H.; Xu, G. Remote C−H Activation Strategy Enables Total Syntheses of Nortriterpenoids (±)-Walsucochin B and (±)-Walsucochinoids M and N. *Org. Lett.* **2020**, *22*, 6993–6997.

5. Rojas de la Parra, V.; Mierau, V.; Anke, T.; Sterner, O. Cytotoxic Terpenoids from Dasyscyphus niveus. *Tetrahedron* **2006**, *62,* 1828–1832.

6. Frisch, M. J.; Trucks, G. W.; Schlegel, H. B.; Scuseria, G. E.; Robb, M. A.; Cheeseman, J. R.; Scalmani G.; Barone, V.; Petersson, G. A.; Nakatsuji, H.; Li, X.; Caricato, M.; Marenich, A. V.; Bloino, J.; Janesko, B. G.; Gomperts, R.; Mennucci, B.; Hratchian, H. P.; Ortiz, J. V.; Izmaylov, A. F.; Sonnenberg, J. L.; Williams-Young, D.; Ding, F.; Lipparini, F.; Egidi, F.; Goings, J.; Peng, B.; Petrone, A.; Henderson, T., Ranasinghe, D.; Zakrzewski, V. G.; Gao, J.; Rega, N.; Zheng, G.; Liang, W.; Hada, M.; Ehara, M.; Toyota, K.; Fukuda, R.; Hasegawa, J.; Ishida, M.; Nakajima, T.; Honda, Y.; Kitao, O.; Nakai, H.; Vreven, T.; Throssell, K.; Montgomery, J. A., Jr.; Peralta, J. E.; Ogliaro, F.; Bearpark, M. J.; Heyd, J. J.; Brothers, E. N.; Kudin, K. N.; Staroverov, V. N.; Keith, T. A.; Kobayashi, R.; Normand, J.; Raghavachari, K.; Rendell, A. P.; Burant, J. C.; Iyengar, S. S.; Tomasi, J.; Cossi, M.; Millam, J. M.; Klene, M.; Adamo, C.; Cammi, R.; Ochterski, J. W.; Martin, R. L.; Morokuma, K.; Farkas, O.; Foresman, J. B.; Fox, D. J. *Gaussian 16 Rev. A.03*. Gaussian, Inc., Wallingford, CT. **2016**.

7. Weigend, F.; Ahlrichs, R. Balanced basis sets of split valence, triple zeta valence and quadruple zeta valence quality for H to Rn: design and assessment of accuracy. *Phys. Chem. Chem. Phys.* **2005**, *7*, 3297–3305.

8. Weigend, F. Accurate Coulomb-fitting basis sets for H to Rn. *Phys. Chem. Chem. Phys*. **2006**, *8*, 1057–1065.

9. Dennington, R.; Keith, T. A.; Millam, J. M. *GaussView Version 6*; Semichem Inc.: Shawnee Mission, KS, 2016.

10. Lu, T.; Chen, Q. Shermo: a general code for calculating molecular thermochemistry properties. *Comput. Theor. Chem.* **2020**, 113249.

11. NMR spectra

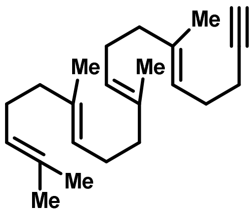

11, 3:1 *E/Z*

^{1}H NMR, 600 MHz, $CDCl_3$

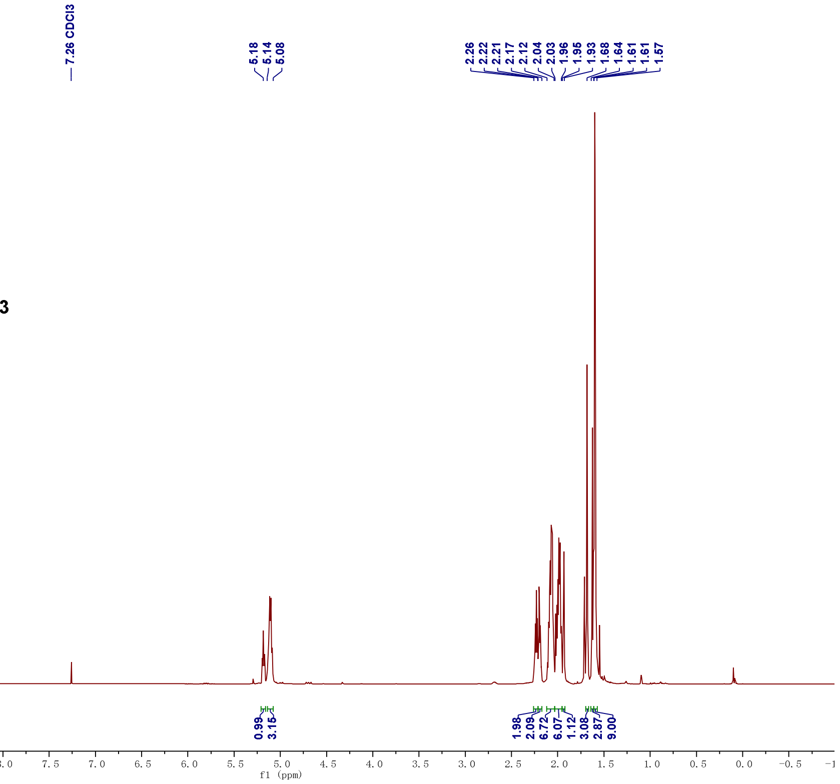

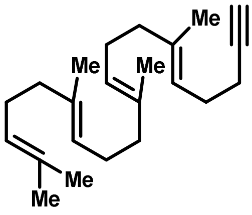

11, 3:1 *E/Z*

^{13}C NMR, 151 MHz, $CDCl_3$

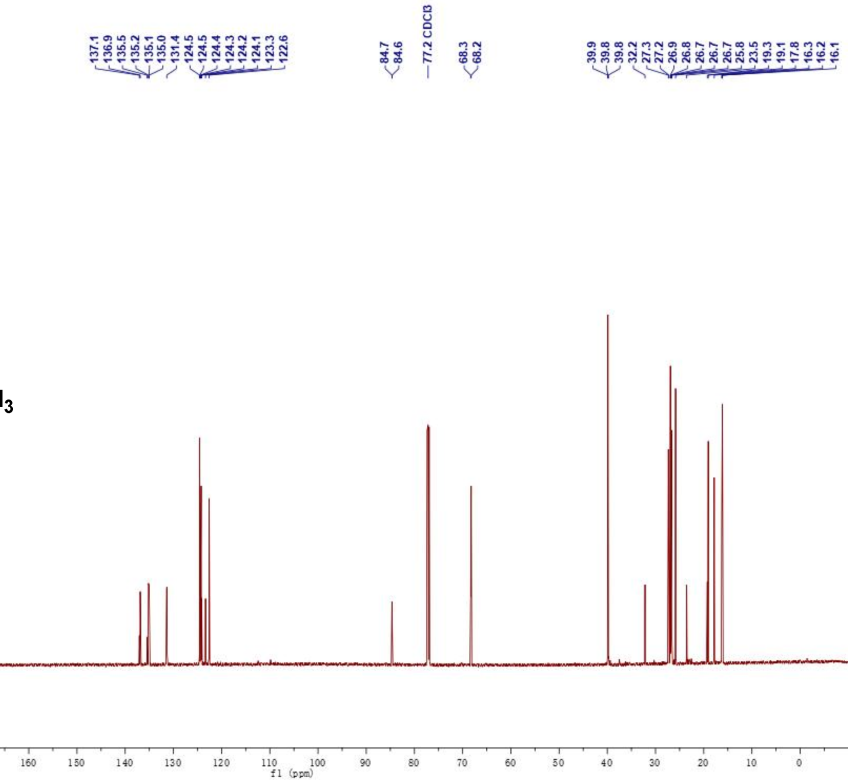

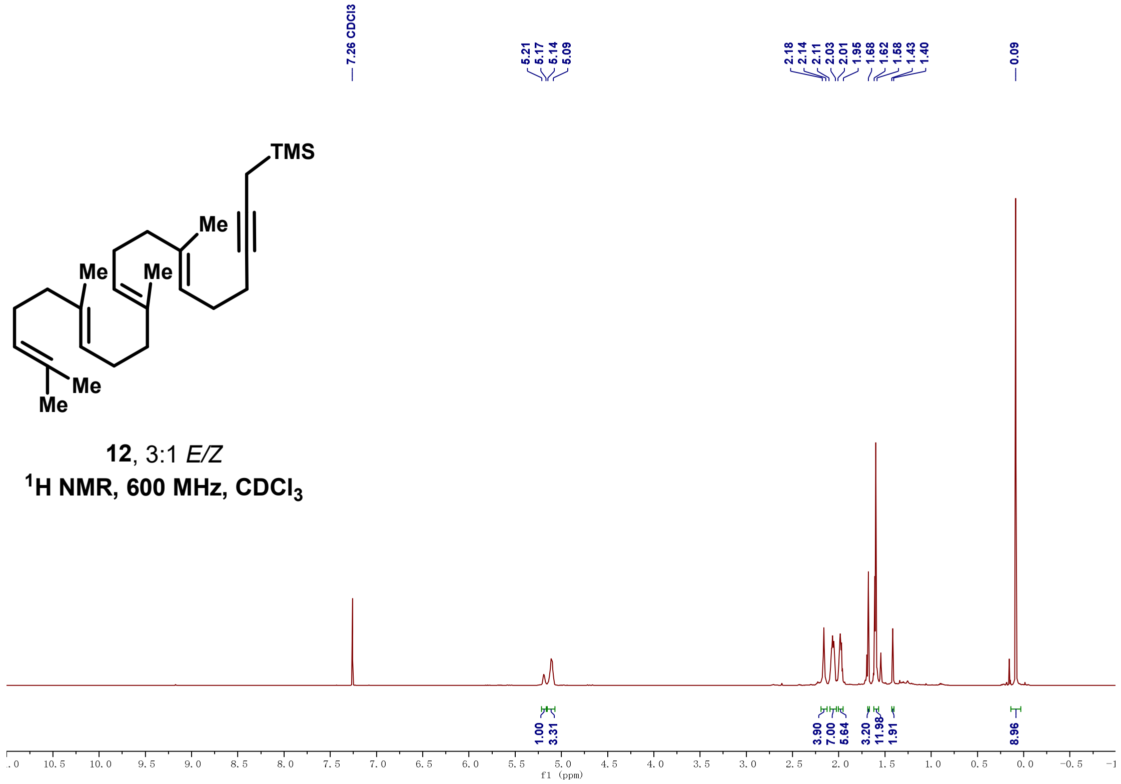
TMS
Me
Me
Me
Me
Me
12, 3:1 E/Z
^{1}H NMR, 600 MHz, $CDCl_3$
7.26 CDCl3
5.21
5.17
5.14
5.09
2.18
2.14
2.11
2.03
2.01
1.96
1.68
1.62
1.58
1.43
1.40
0.09
1.00
3.31
3.90
7.00
5.64
3.20
11.98
1.91
8.96
f1 (ppm)

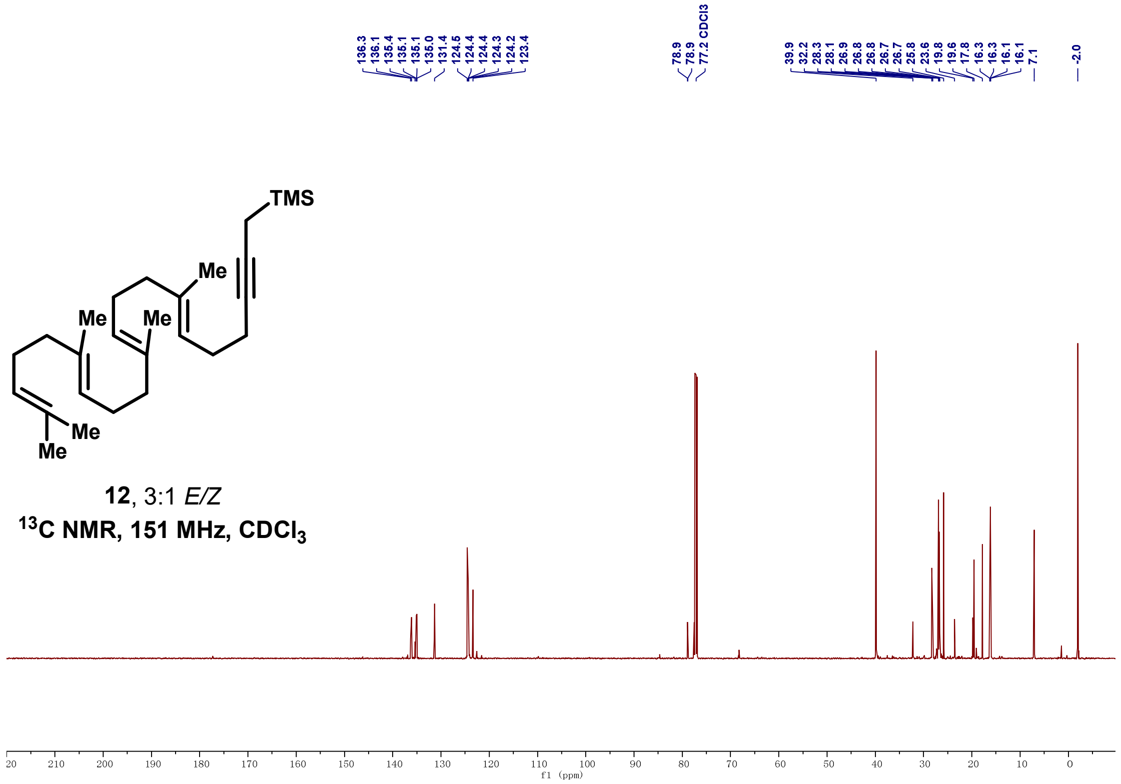
136.3
136.1
135.4
135.1
135.1
135.0
131.4
124.5
124.4
124.4
124.3
124.2
123.4
78.9
78.9
77.2 CDCl3
39.9
32.2
28.3
28.1
26.9
26.8
26.8
26.7
26.7
25.8
23.6
19.8
19.6
17.8
16.3
16.1
16.1
7.1
-2.0
TMS
Me
Me
Me
Me
Me
12, 3:1 E/Z
^{13}C NMR, 151 MHz, $CDCl_3$
f1 (ppm)

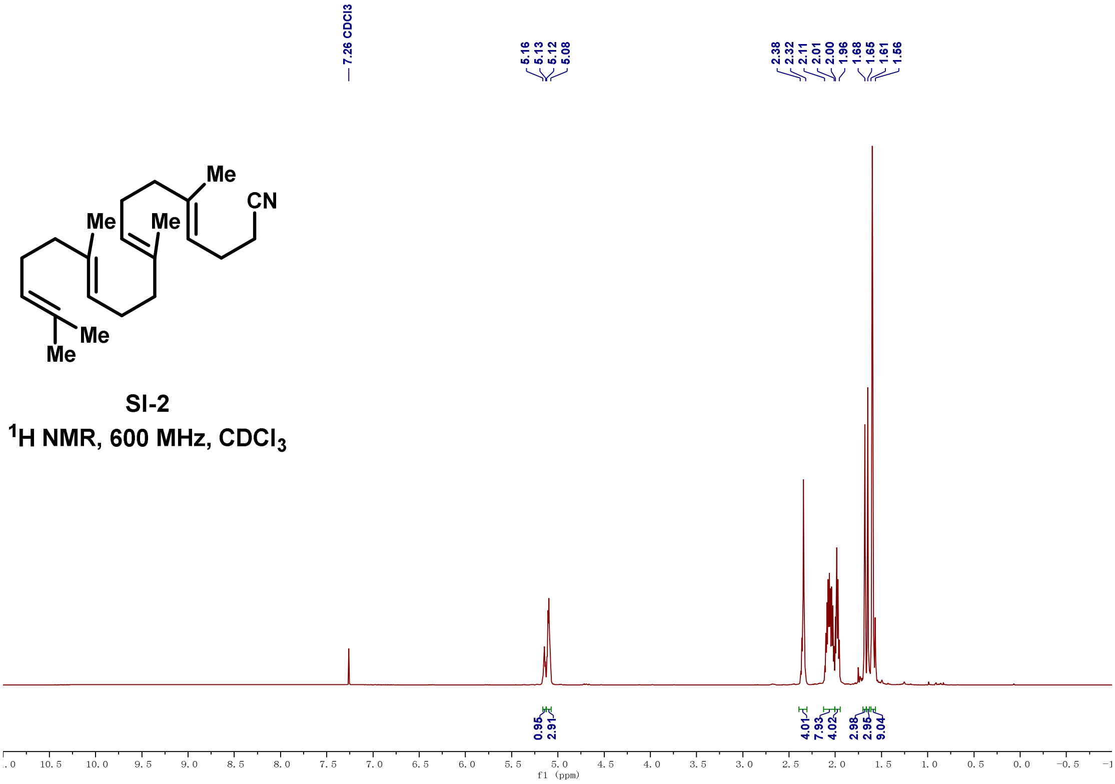

SI-2
^{1}H NMR, 600 MHz, $CDCl_3$

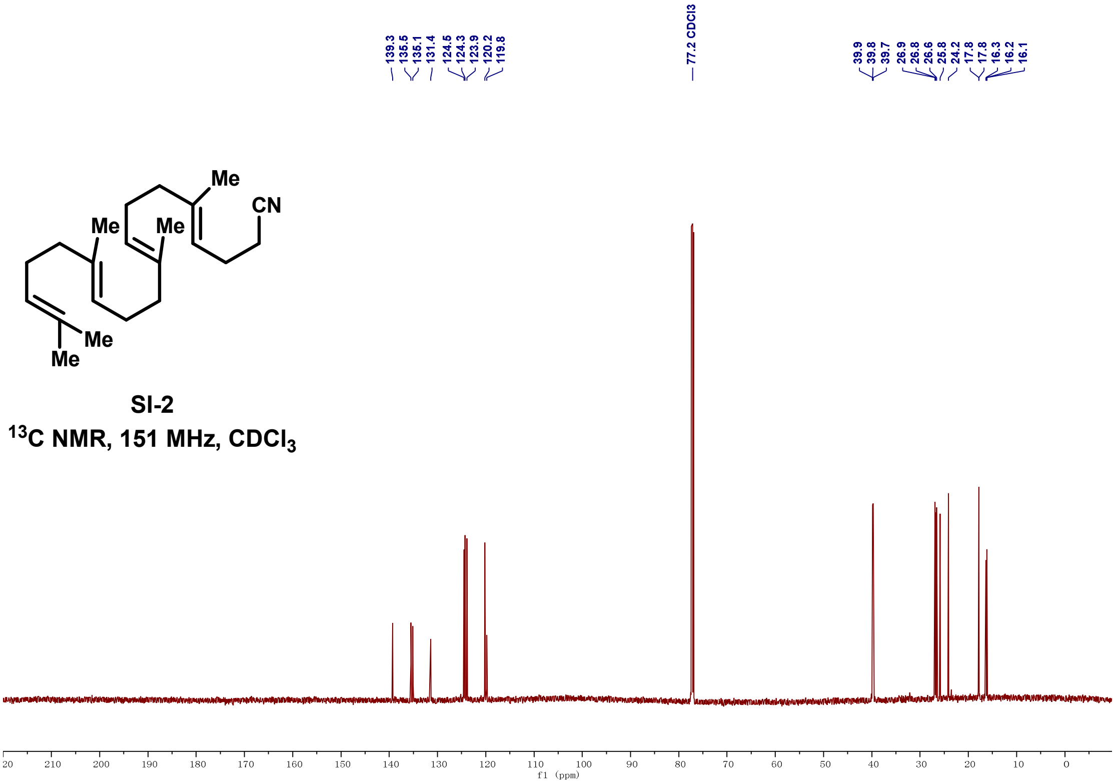

SI-2
^{13}C NMR, 151 MHz, $CDCl_3$

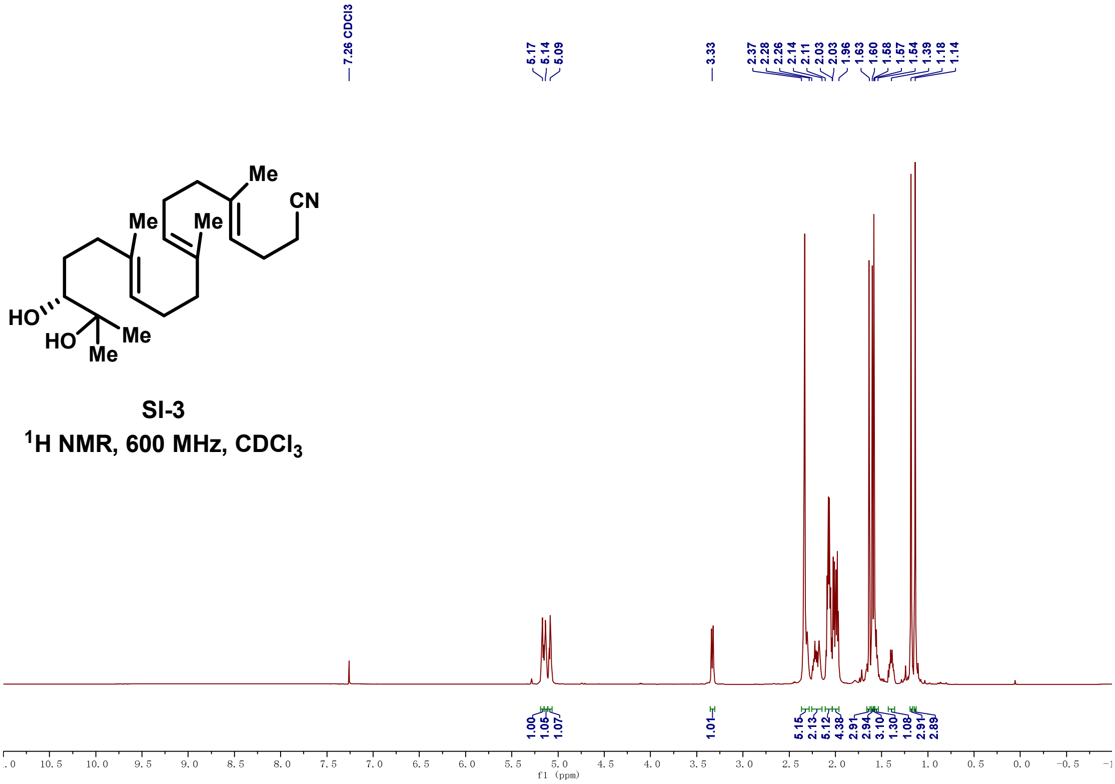

SI-3
^{1}H NMR, 600 MHz, $CDCl_3$

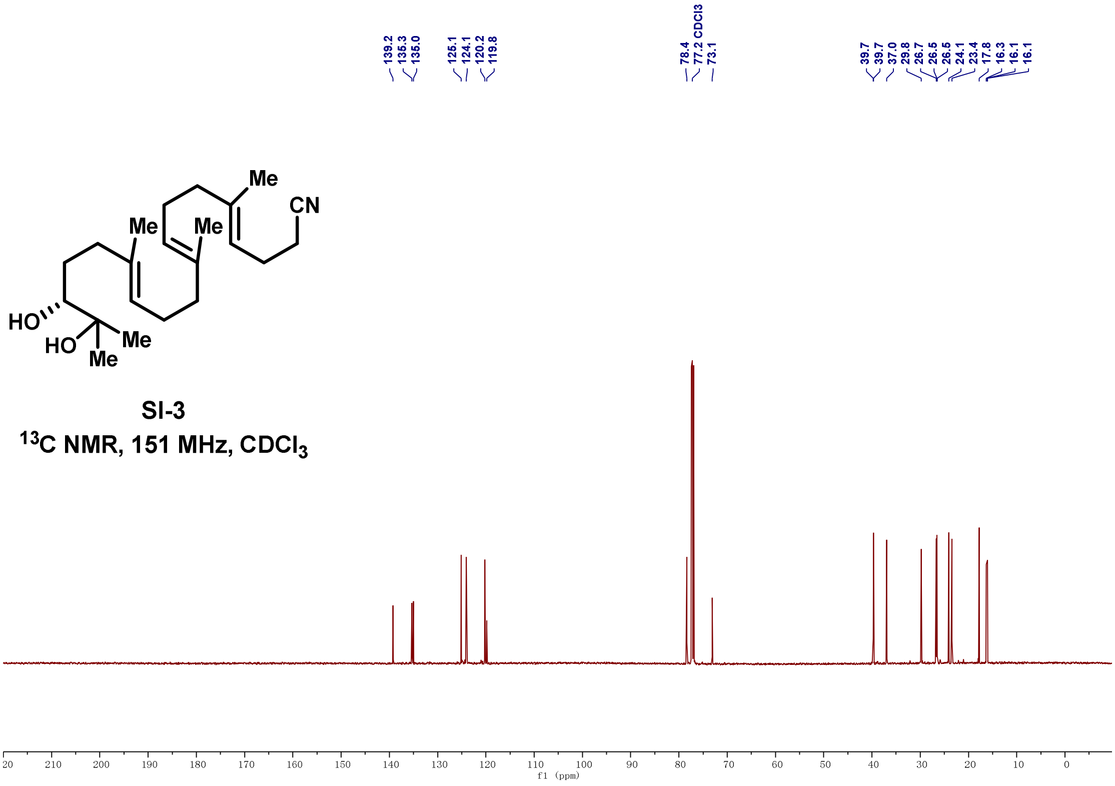

SI-3
^{13}C NMR, 151 MHz, $CDCl_3$

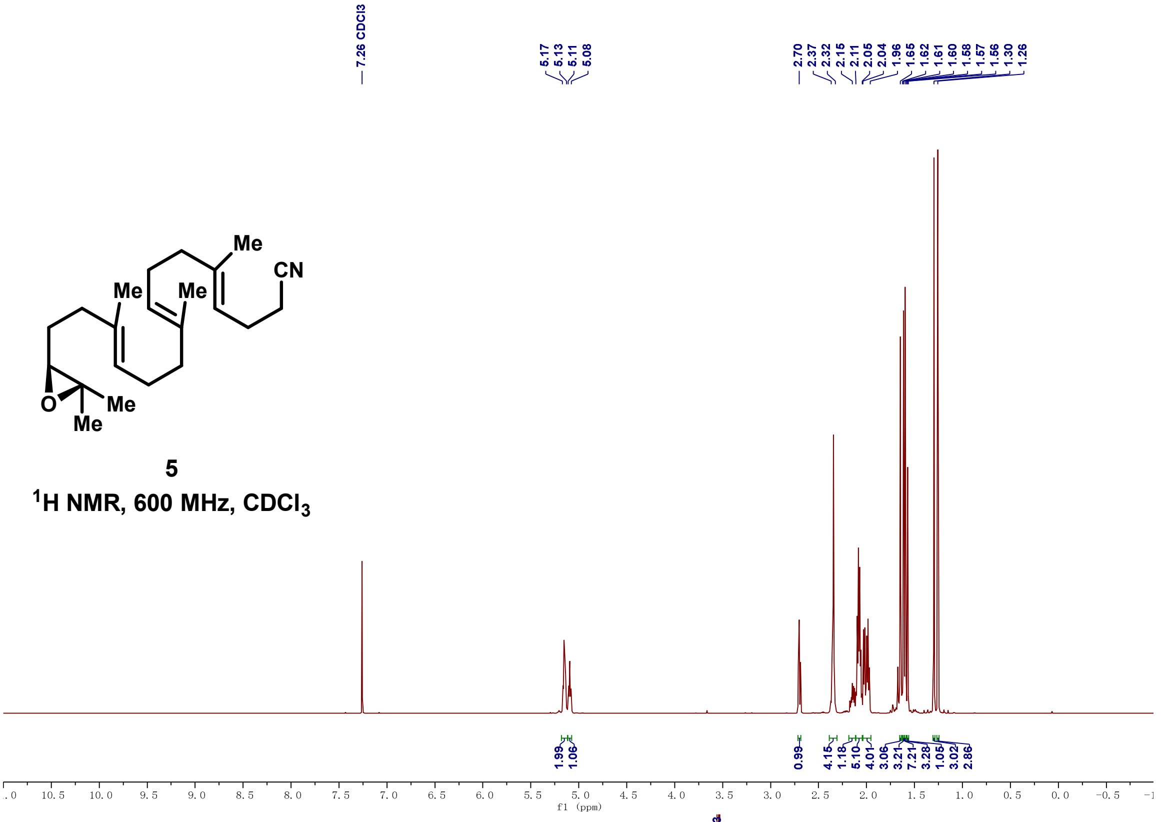

5

^{1}H NMR, 600 MHz, $CDCl_3$

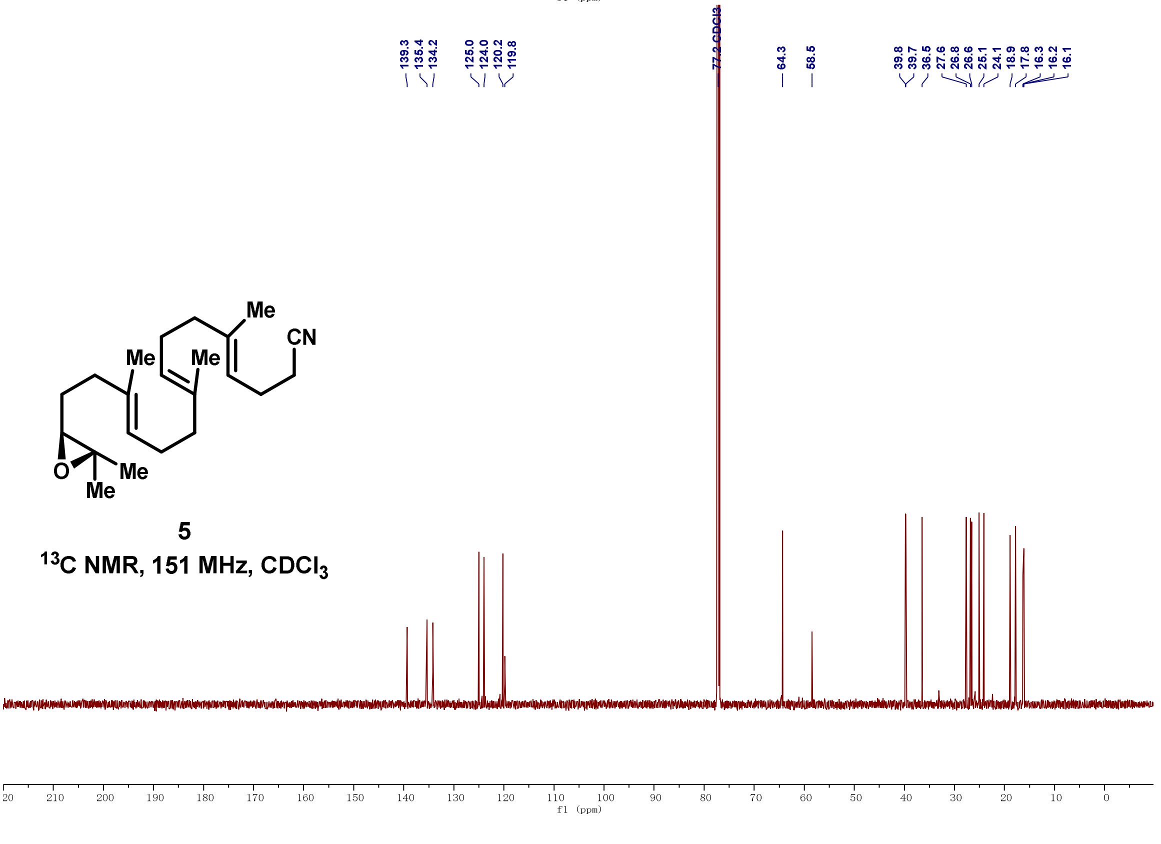

5

^{13}C NMR, 151 MHz, $CDCl_3$

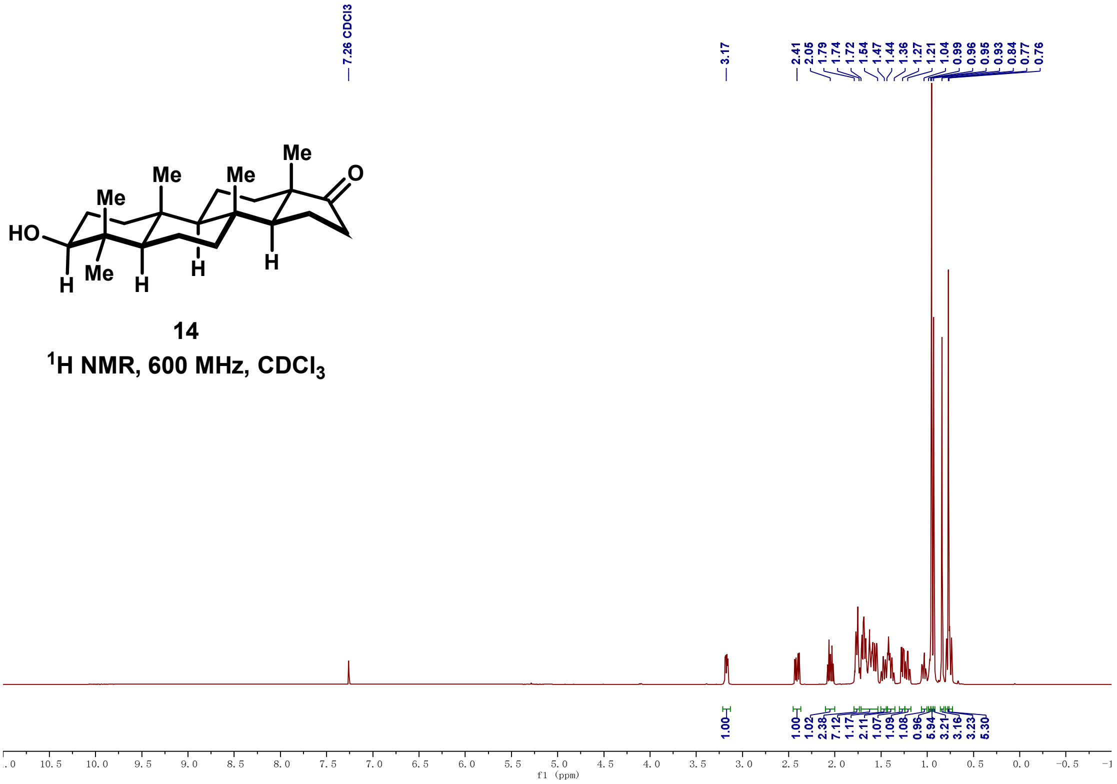
7.26 CDCl3
3.17
2.41 2.06 1.79 1.74 1.72 1.54 1.47 1.44 1.36 1.27 1.21 1.04 0.99 0.96 0.95 0.93 0.84 0.77 0.76
1.00 1.02 2.38 7.12 1.17 2.11 1.07 1.09 1.08 0.96 5.94 3.21 3.16 3.23 6.30
f1 (ppm)
14
^{1}H NMR, 600 MHz, $CDCl_3$

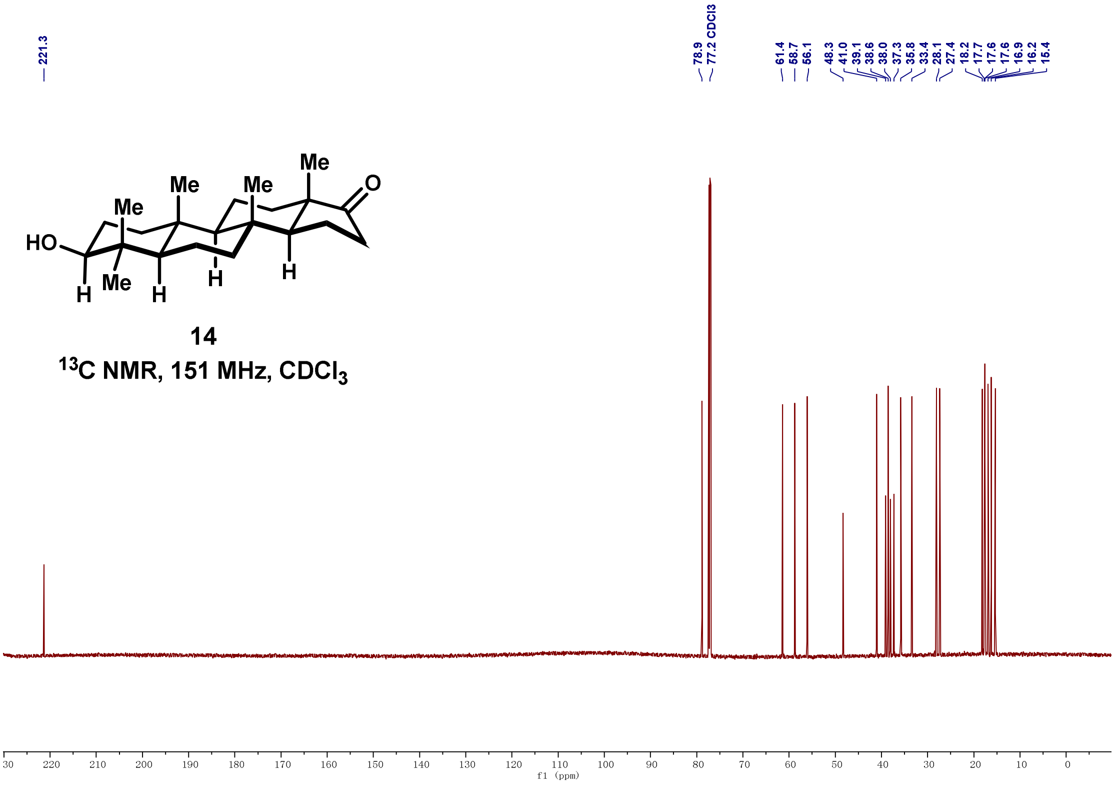
221.3
78.9 77.2 CDCl3
61.4 58.7 56.1
48.3 41.0 39.1 38.6 38.0 37.3 35.8 33.4 28.1 27.4 18.2 17.7 17.6 16.9 16.2 15.4
f1 (ppm)
14
^{13}C NMR, 151 MHz, $CDCl_3$

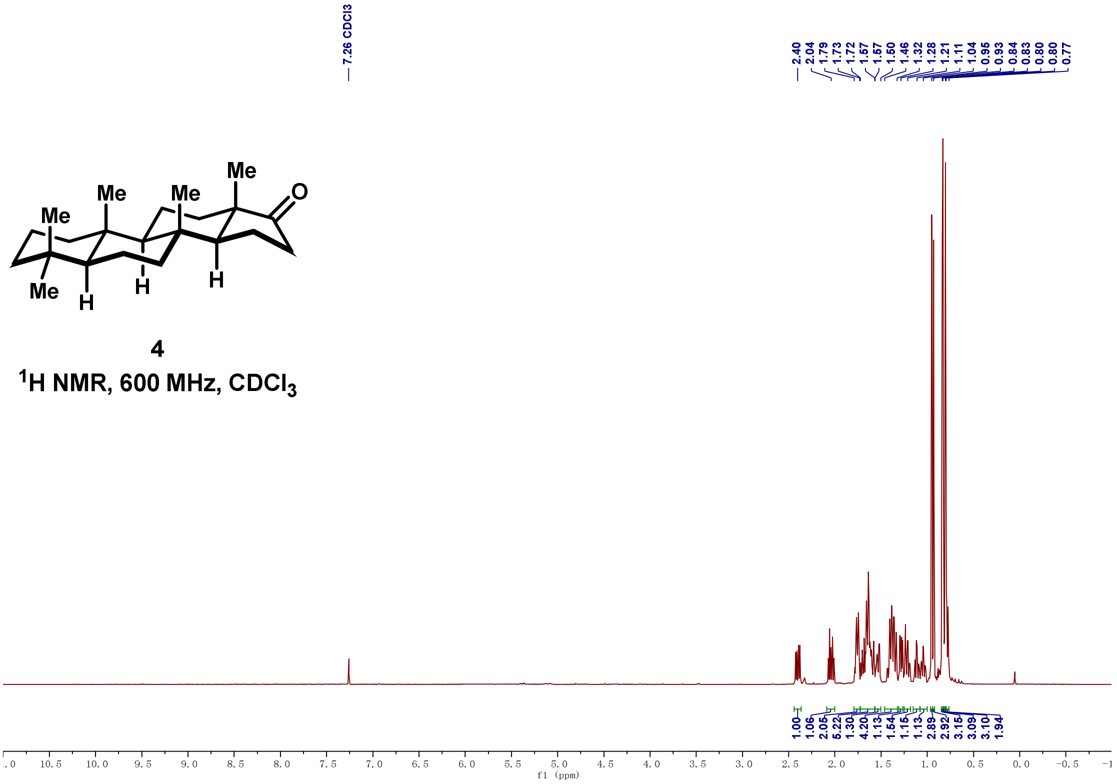

4

^{1}H NMR, 600 MHz, $CDCl_3$

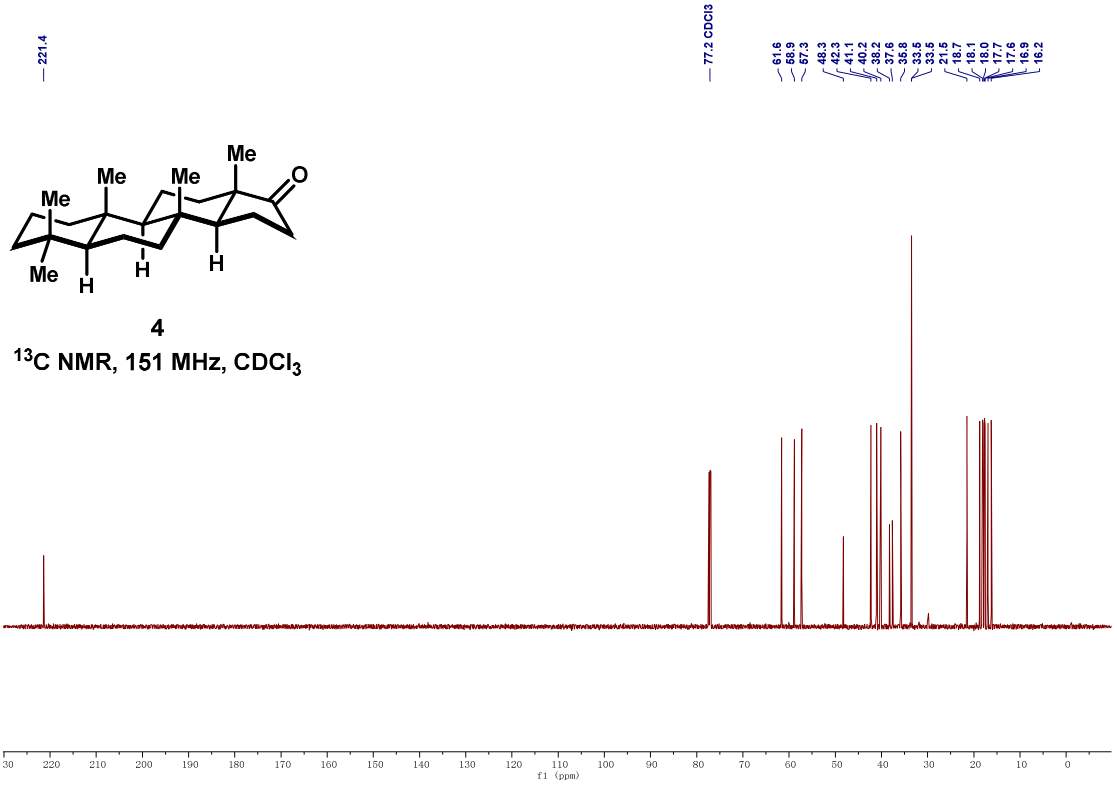

4

^{13}C NMR, 151 MHz, $CDCl_3$

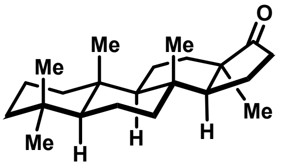

3

^{1}H NMR, 600 MHz, $CDCl_3$

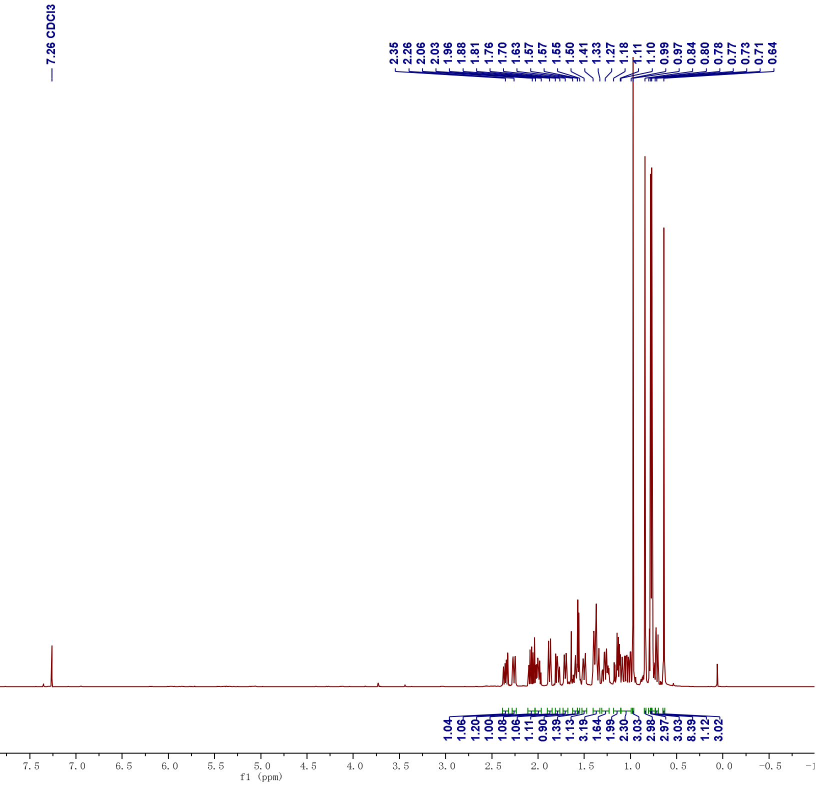

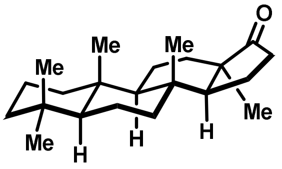

3

^{13}C NMR, 151 MHz, $CDCl_3$

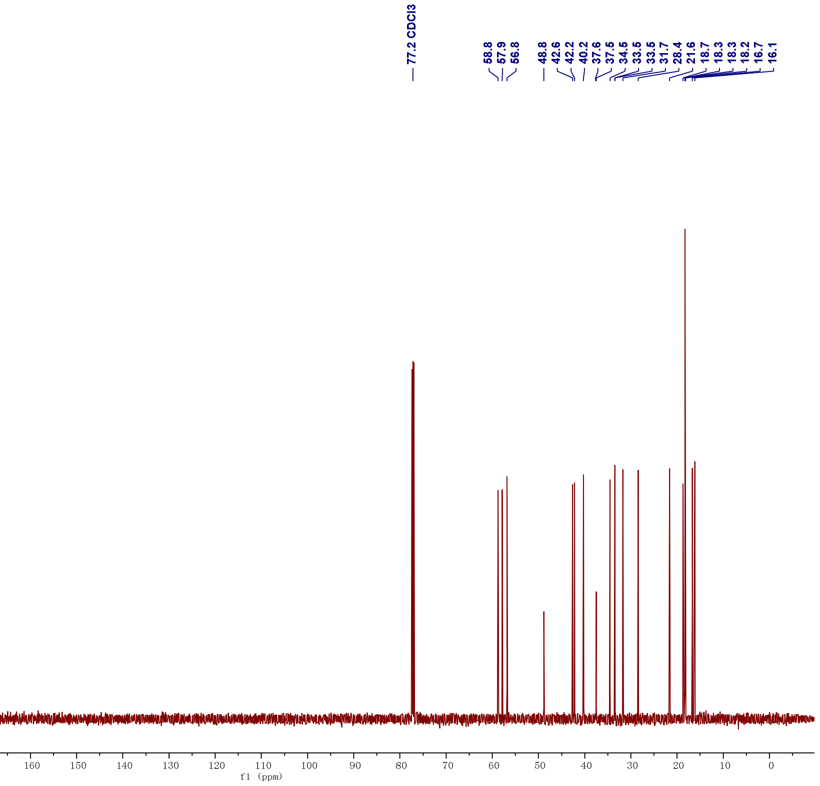

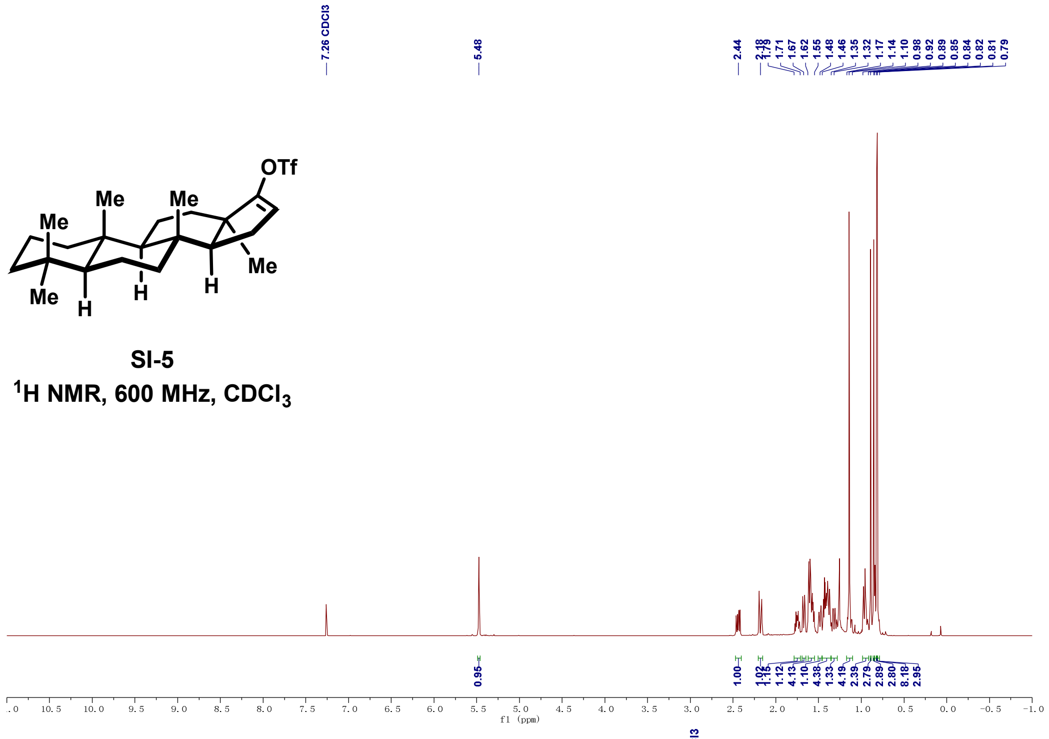

SI-5

^{1}H NMR, 600 MHz, $CDCl_3$

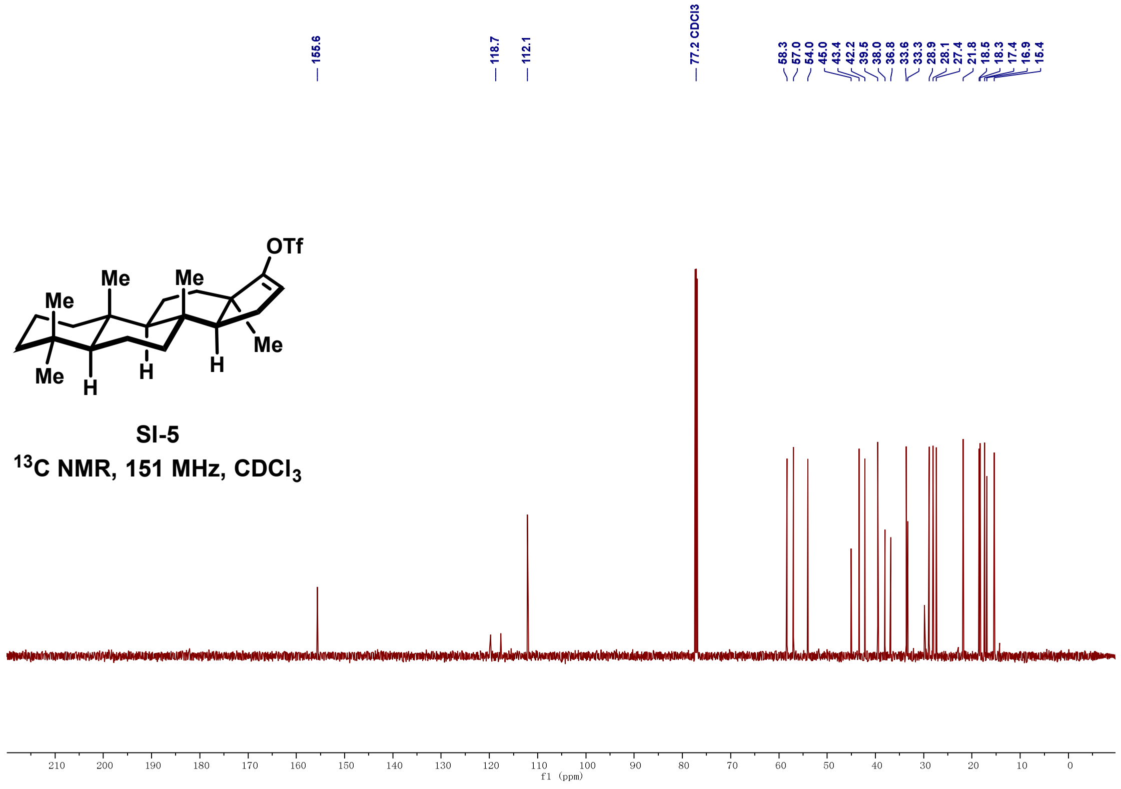

SI-5

^{13}C NMR, 151 MHz, $CDCl_3$

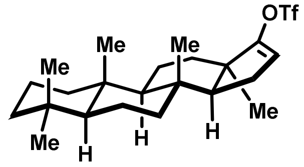

SI-5

^{19}F NMR, 471 MHz, $CDCl_3$

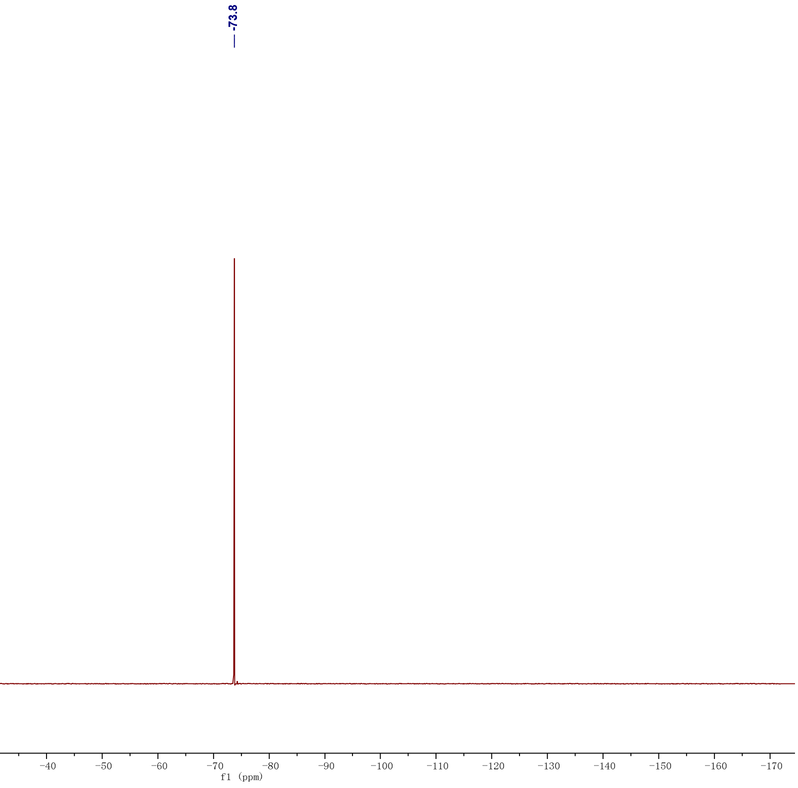

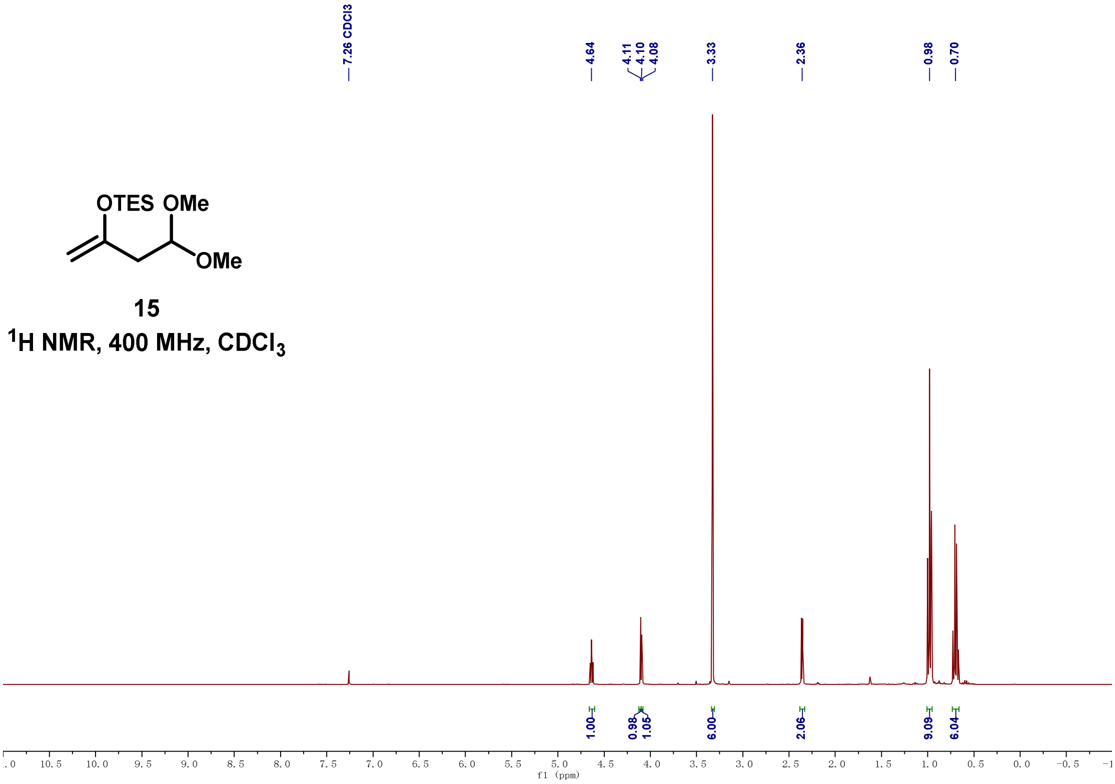
7.26 CDCl3
4.64
4.11
4.10
4.08
3.33
2.36
0.98
0.70
OTES OMe
OMe
15
¹H NMR, 400 MHz, CDCl3
1.00
0.98
1.06
6.00
2.06
9.09
6.04
f1 (ppm)

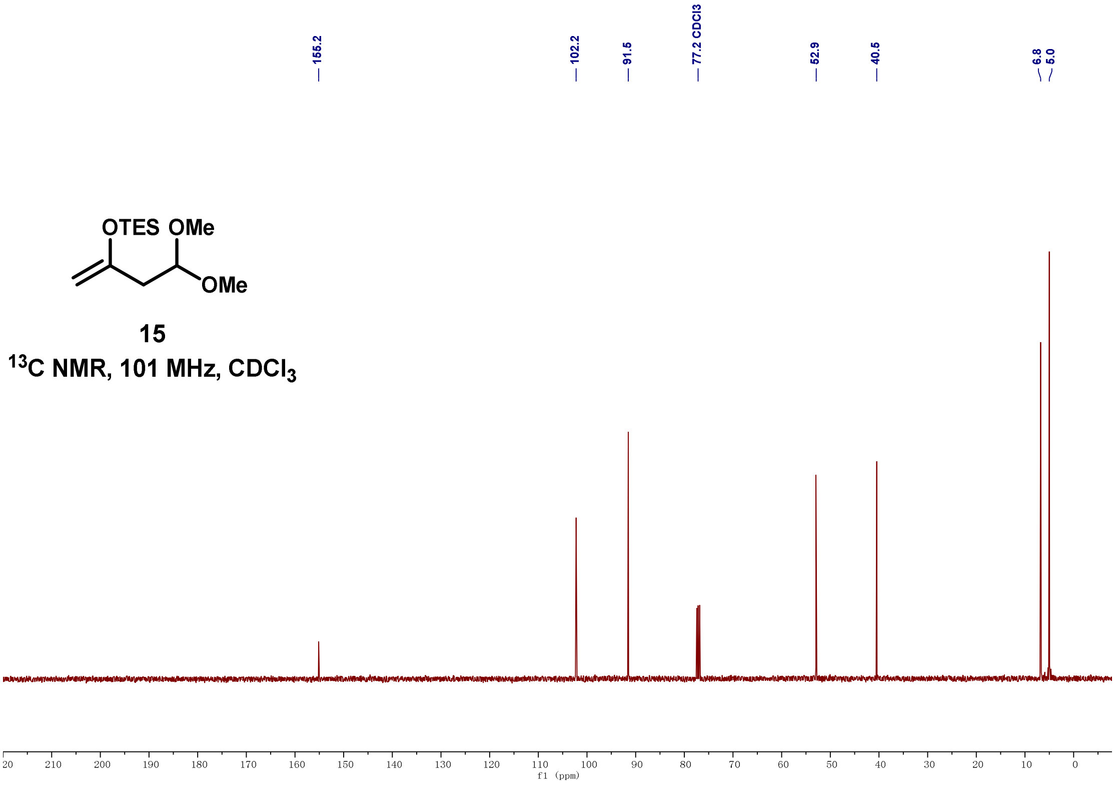
155.2
102.2
91.5
77.2 CDCl3
52.9
40.5
6.8
5.0
OTES OMe
OMe
15
¹³C NMR, 101 MHz, CDCl3
f1 (ppm)

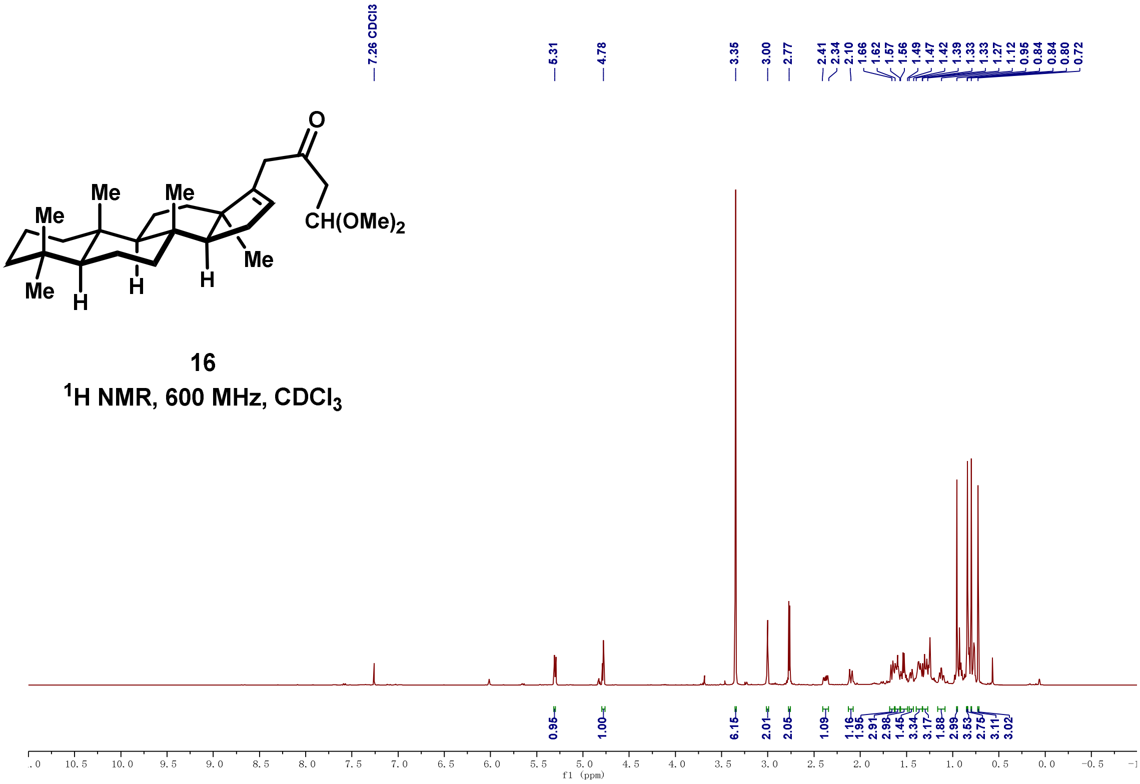

16

^{1}H NMR, 600 MHz, $CDCl_3$

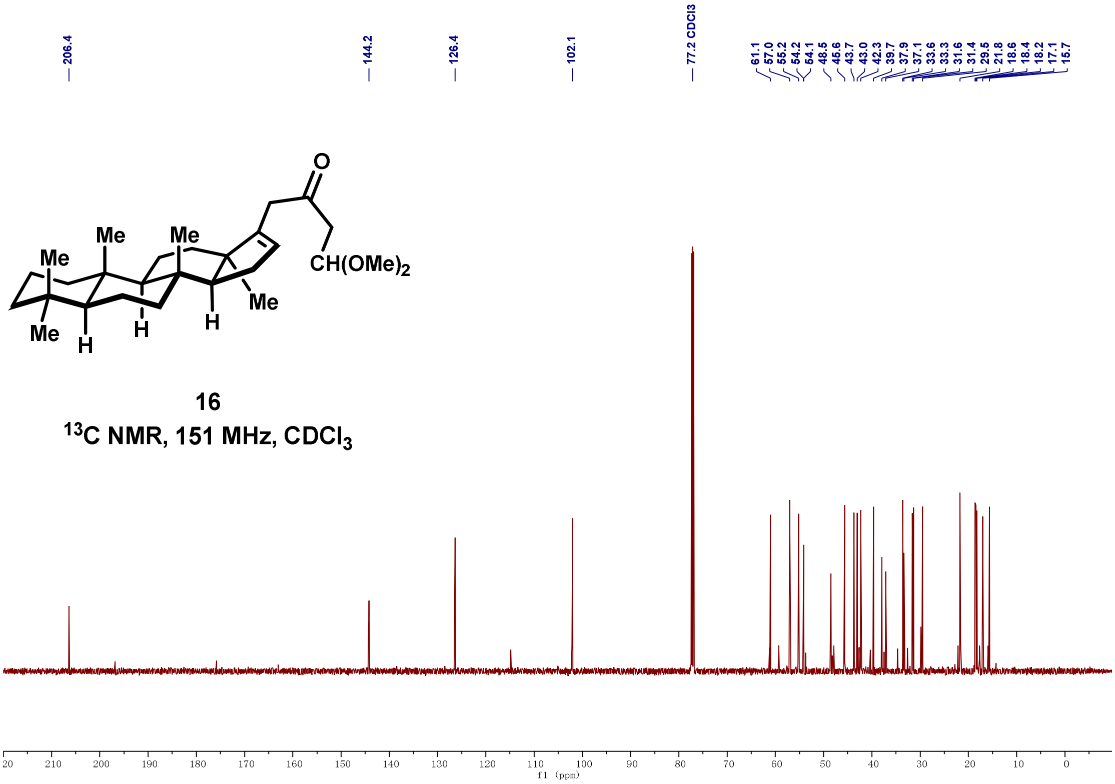

16

^{13}C NMR, 151 MHz, $CDCl_3$

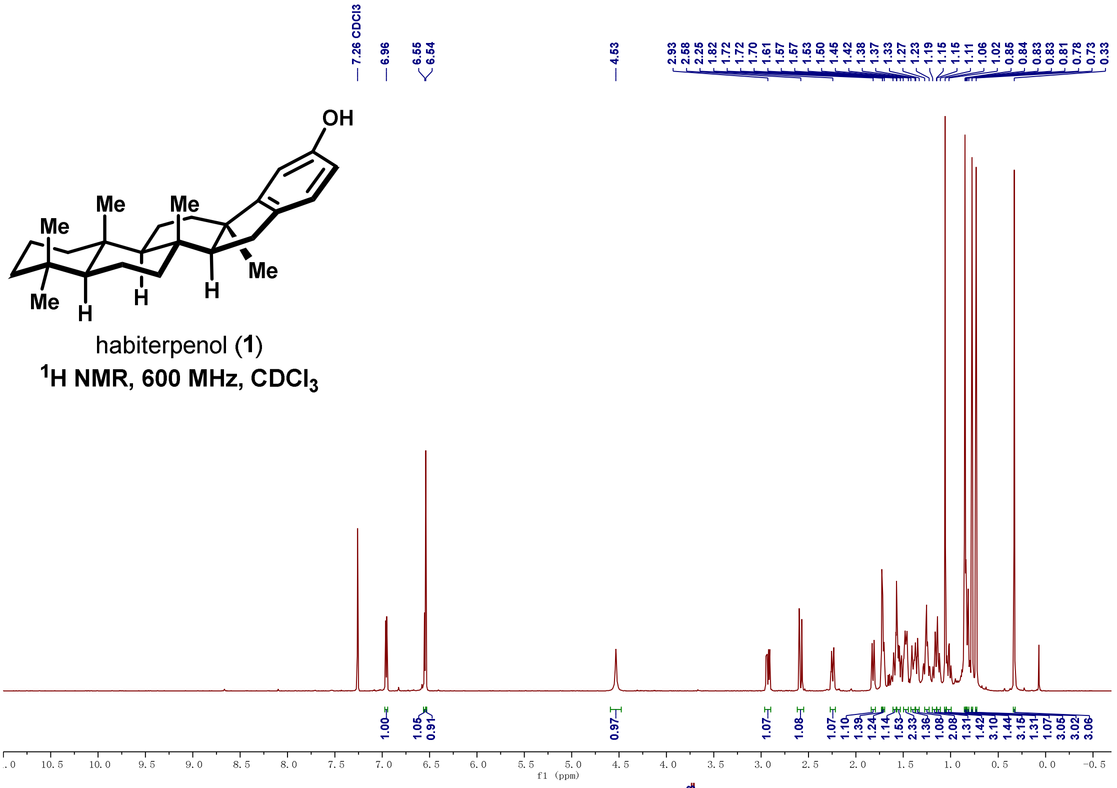
habiterpenol (1)
1H NMR, 600 MHz, CDCl3
OH
Me
Me
Me
Me
Me
H
H
H
7.26 CDCl3
6.96
6.55
6.54
4.53
2.93
2.58
2.25
1.82
1.72
1.72
1.70
1.61
1.57
1.57
1.53
1.50
1.46
1.42
1.38
1.37
1.33
1.27
1.23
1.19
1.16
1.16
1.11
1.06
1.02
0.86
0.84
0.83
0.83
0.81
0.78
0.73
0.33
1.00
1.05
0.91
0.97
1.07
1.08
1.07
1.10
1.39
1.24
1.14
1.53
2.33
1.36
1.08
2.08
1.31
1.42
3.10
1.44
3.15
1.31
1.07
3.05
3.02
3.06
f1 (ppm)

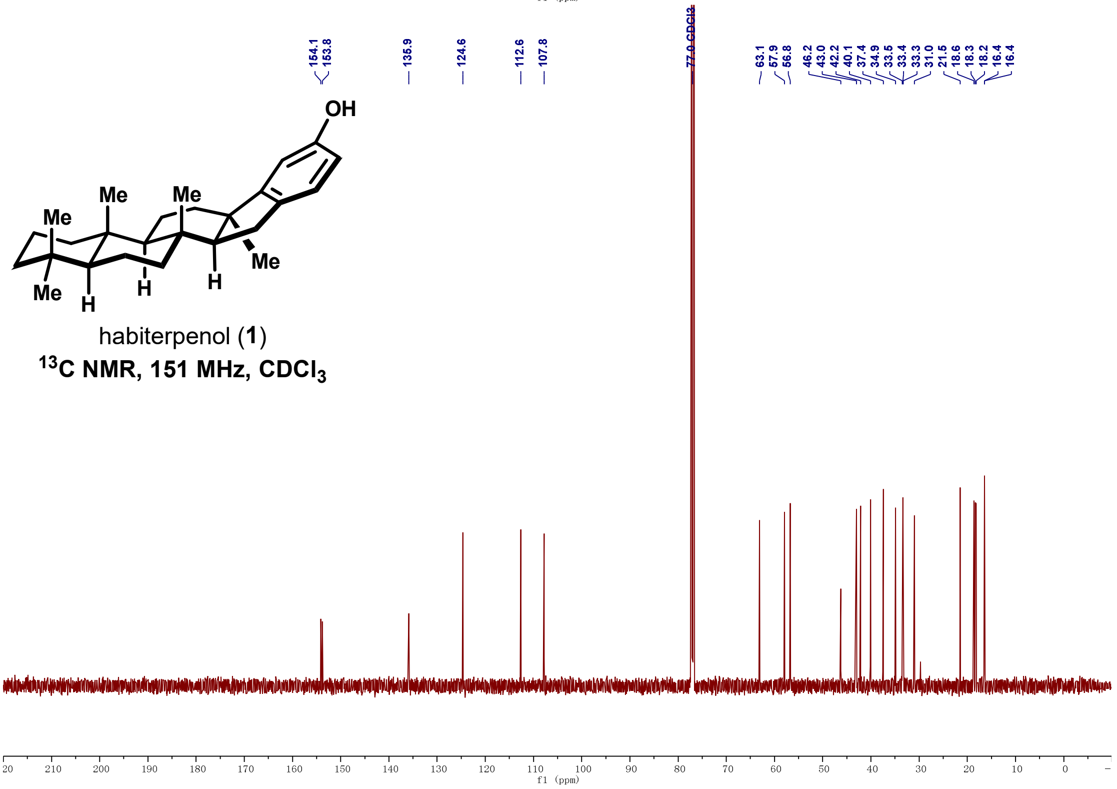
habiterpenol (1)
13C NMR, 151 MHz, CDCl3
OH
Me
Me
Me
Me
Me
H
H
H
154.1
153.8
135.9
124.6
112.6
107.8
77.0 CDCl3
63.1
57.9
56.8
46.2
43.0
42.2
40.1
37.4
34.9
33.5
33.4
33.3
31.0
21.5
18.6
18.3
18.2
16.4
16.4
f1 (ppm)

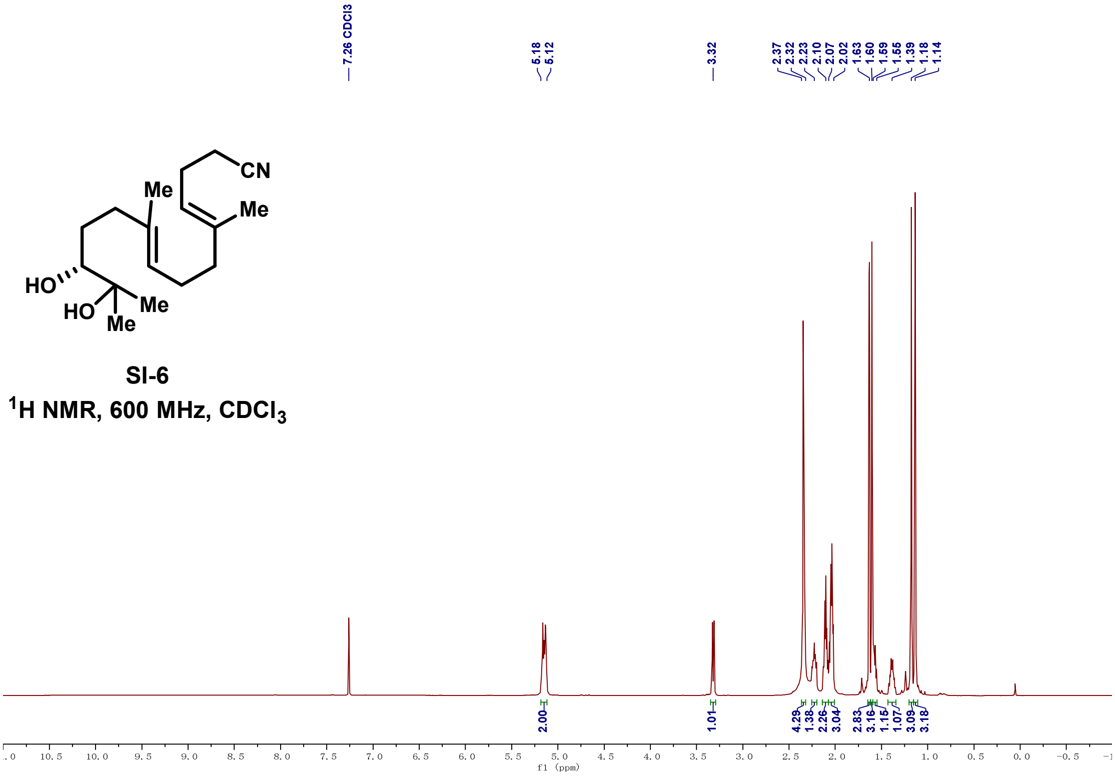
7.26 CDCl3
5.18
5.12
3.32
2.37
2.32
2.23
2.10
2.07
2.02
1.63
1.60
1.59
1.56
1.39
1.18
1.14
CN
Me
Me
HO
HO
Me
Me
SI-6
^{1}H NMR, 600 MHz, $CDCl_3$
2.00
1.01
4.29
1.38
2.26
3.04
2.83
3.16
1.15
1.07
3.09
3.18
f1 (ppm)

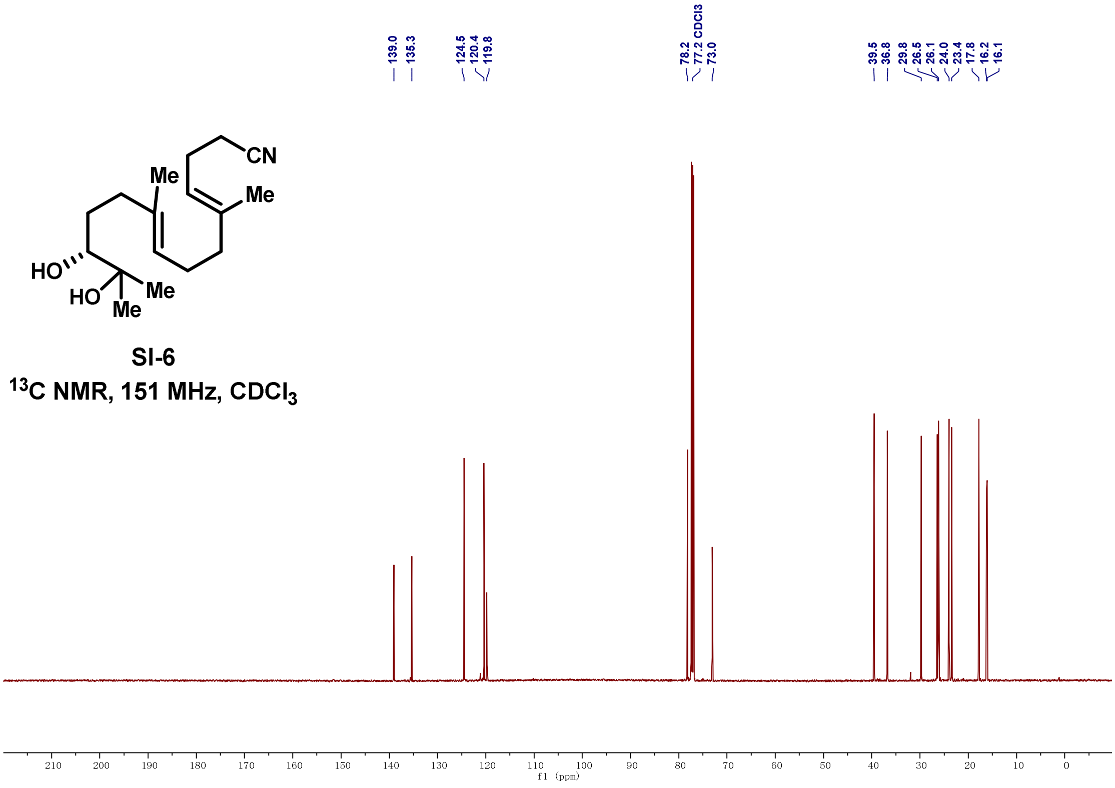
139.0
135.3
124.5
120.4
119.8
78.2
77.2 CDCl3
73.0
39.5
36.8
29.8
26.5
26.1
24.0
23.4
17.8
16.2
16.1
CN
Me
Me
HO
HO
Me
Me
SI-6
^{13}C NMR, 151 MHz, $CDCl_3$
f1 (ppm)

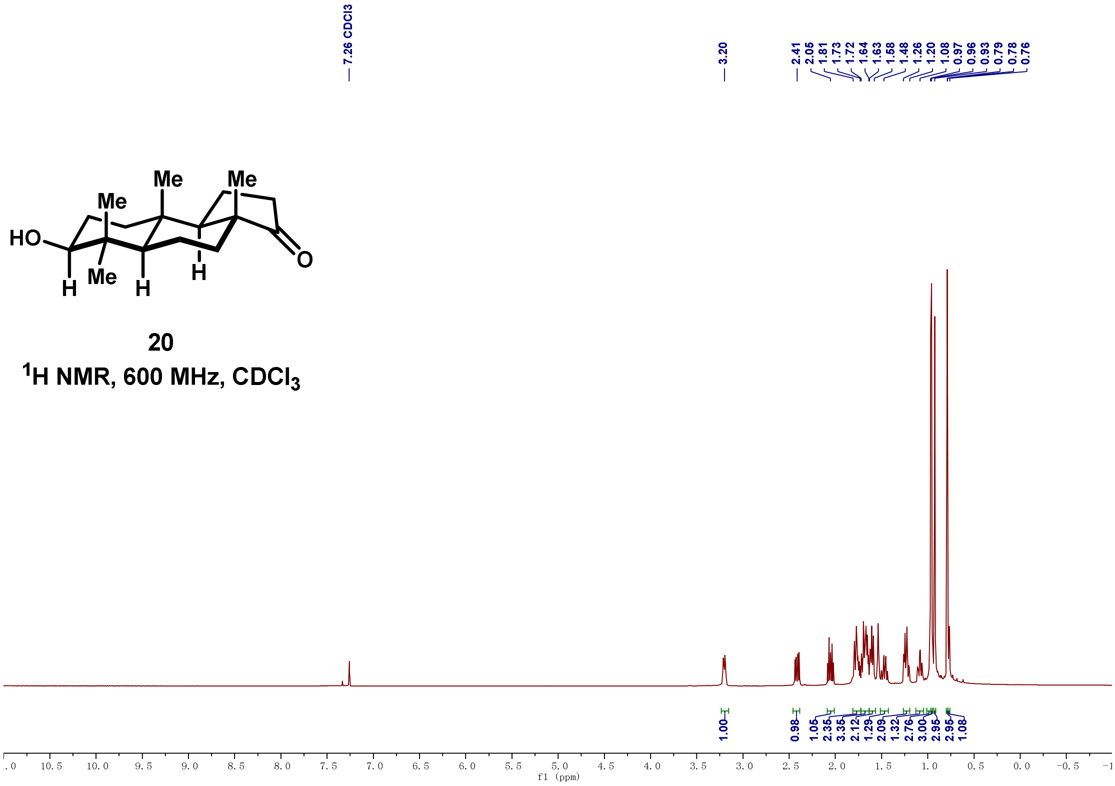

20

^{1}H NMR, 600 MHz, $CDCl_3$

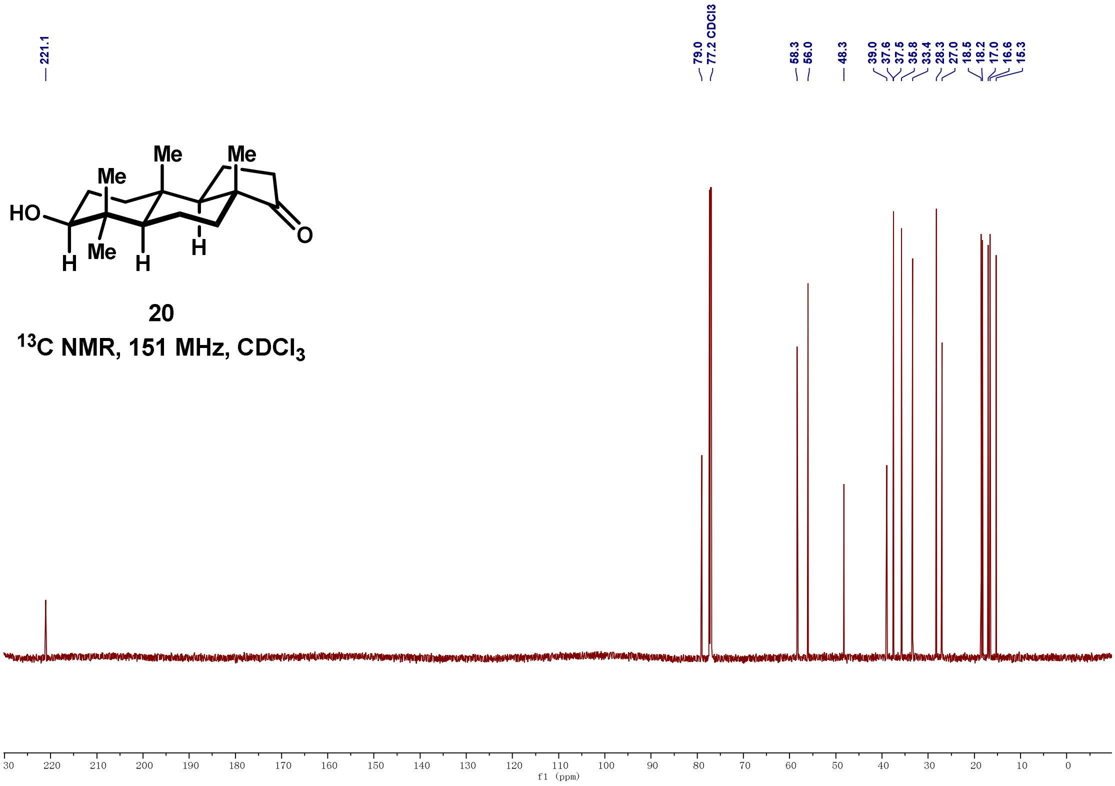

20

^{13}C NMR, 151 MHz, $CDCl_3$

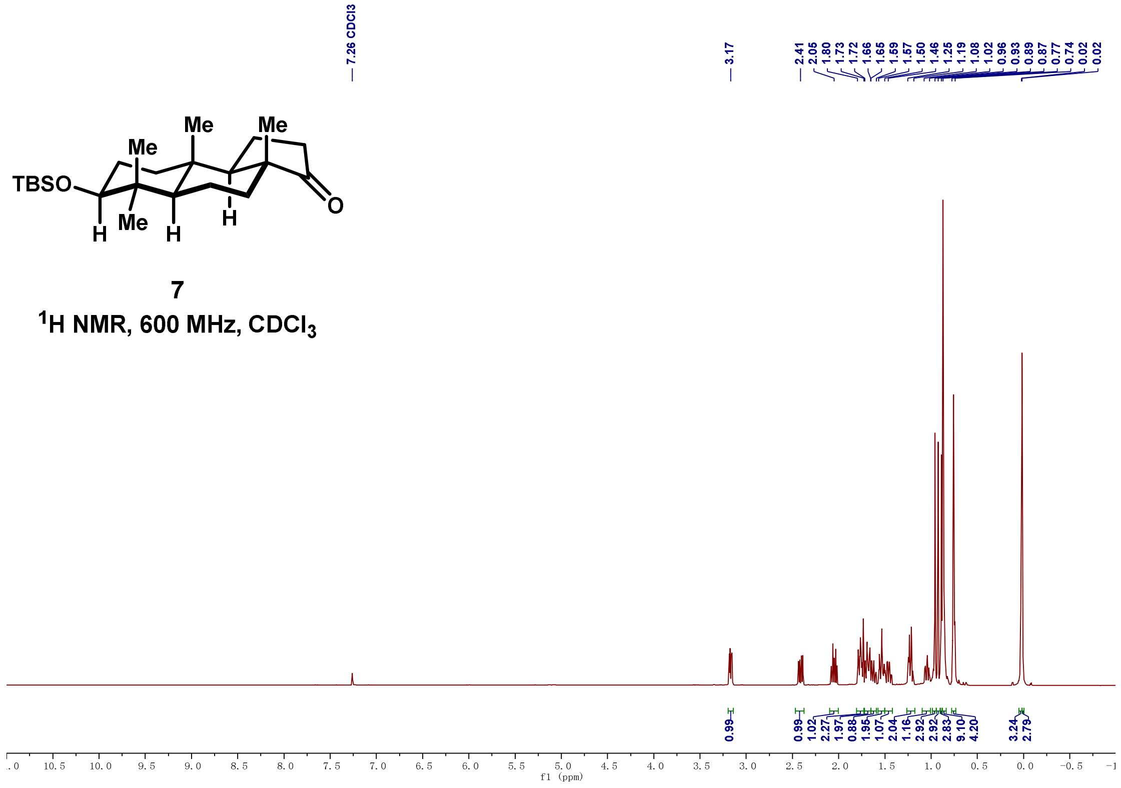
TBSO
Me
Me
Me
Me
H
H
H
O
7
^{1}H NMR, 600 MHz, $CDCl_3$
f1 (ppm)

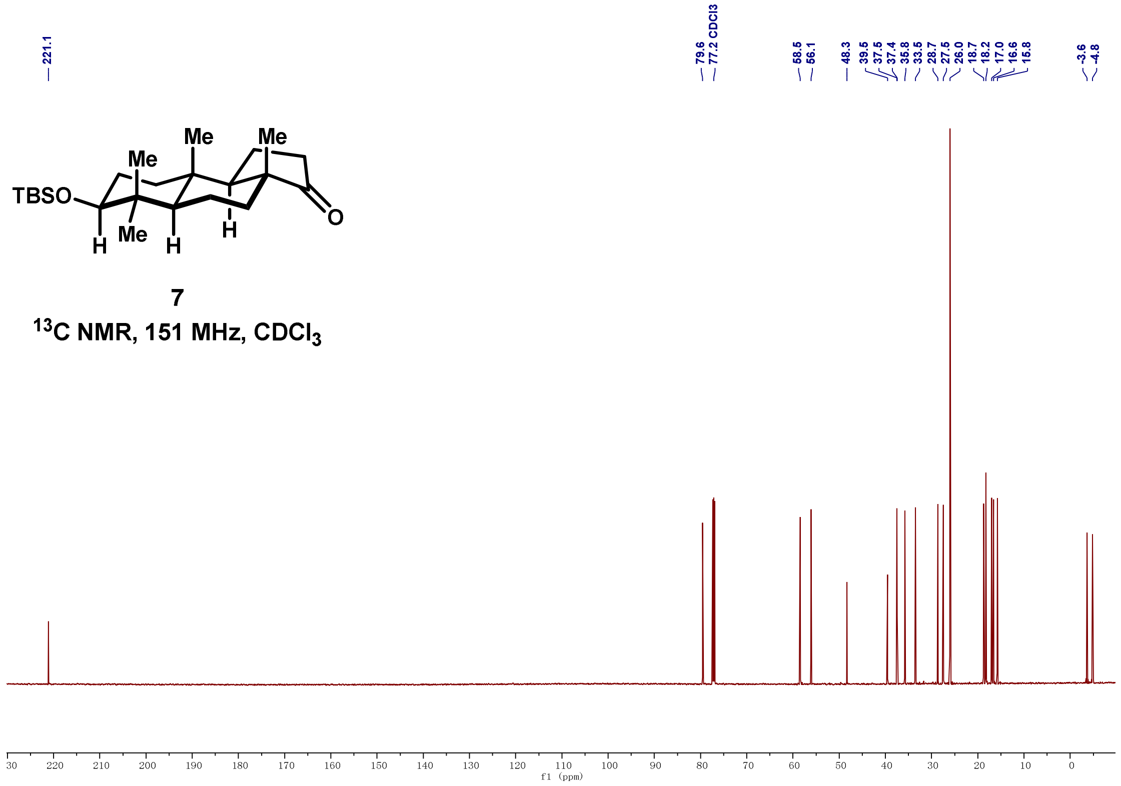
TBSO
Me
Me
Me
Me
H
H
H
O
7
^{13}C NMR, 151 MHz, $CDCl_3$
f1 (ppm)

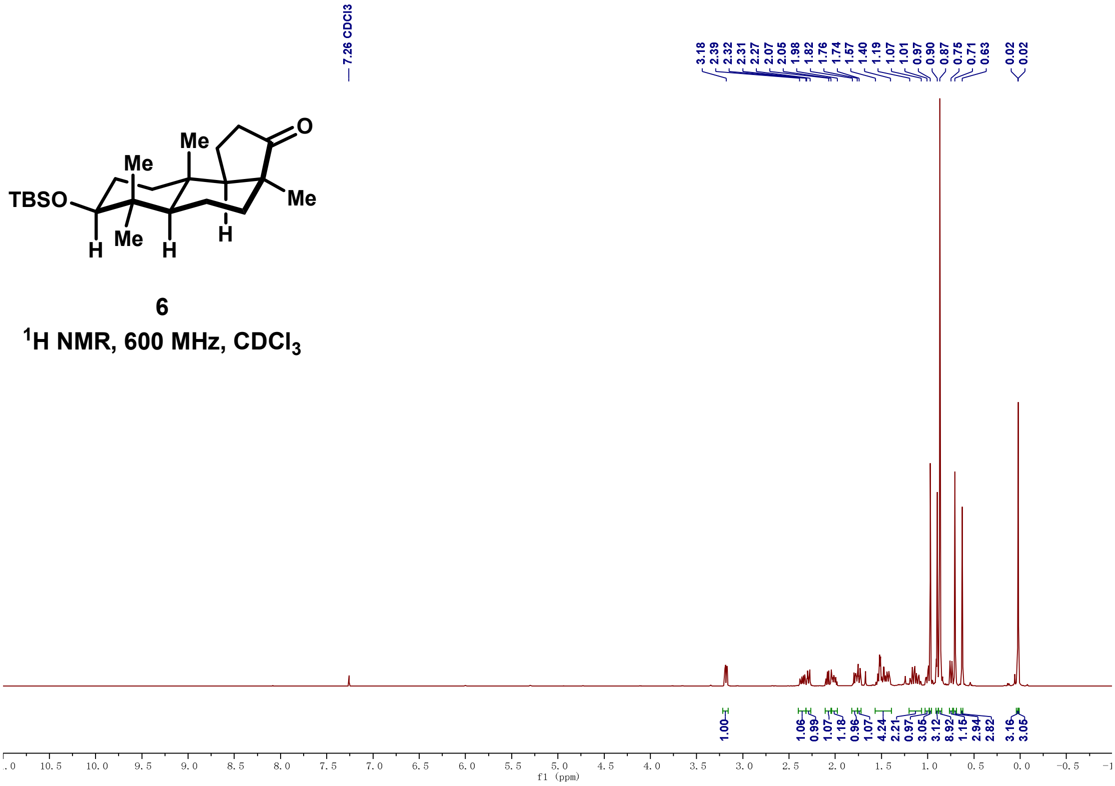
7.26 CDCl3
3.18
2.39
2.32
2.31
2.27
2.07
2.05
1.98
1.82
1.76
1.74
1.57
1.40
1.19
1.07
1.01
0.97
0.90
0.87
0.75
0.71
0.63
0.02
0.02
TBSO
Me
Me
Me
Me
O
H
H
H
6
1H NMR, 600 MHz, CDCl3
1.00
1.06
0.99
1.07
1.18
0.96
1.07
4.24
2.21
0.97
3.05
3.12
8.92
1.16
2.94
2.82
3.16
3.05
f1 (ppm)

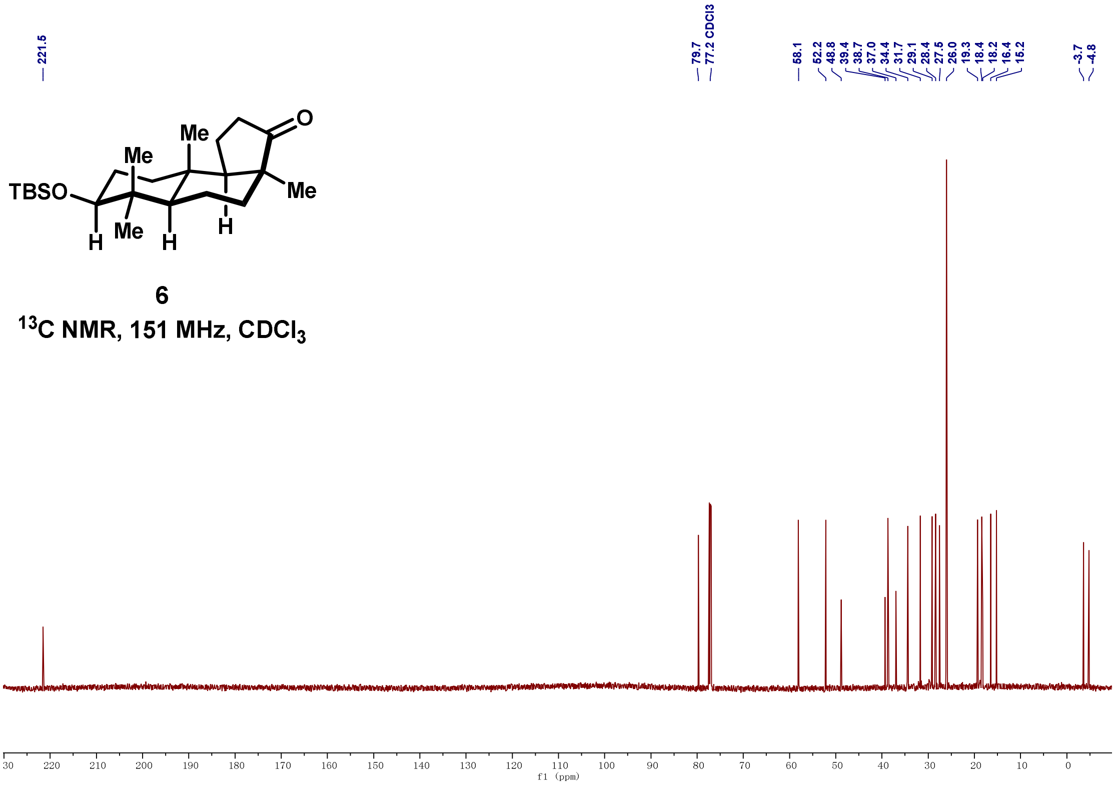
221.5
79.7
77.2 CDCl3
58.1
52.2
48.8
39.4
38.7
37.0
34.4
31.7
29.1
28.4
27.5
26.0
19.3
18.4
18.2
16.4
15.2
-3.7
-4.8
TBSO
Me
Me
Me
Me
O
H
H
H
6
13C NMR, 151 MHz, CDCl3
f1 (ppm)

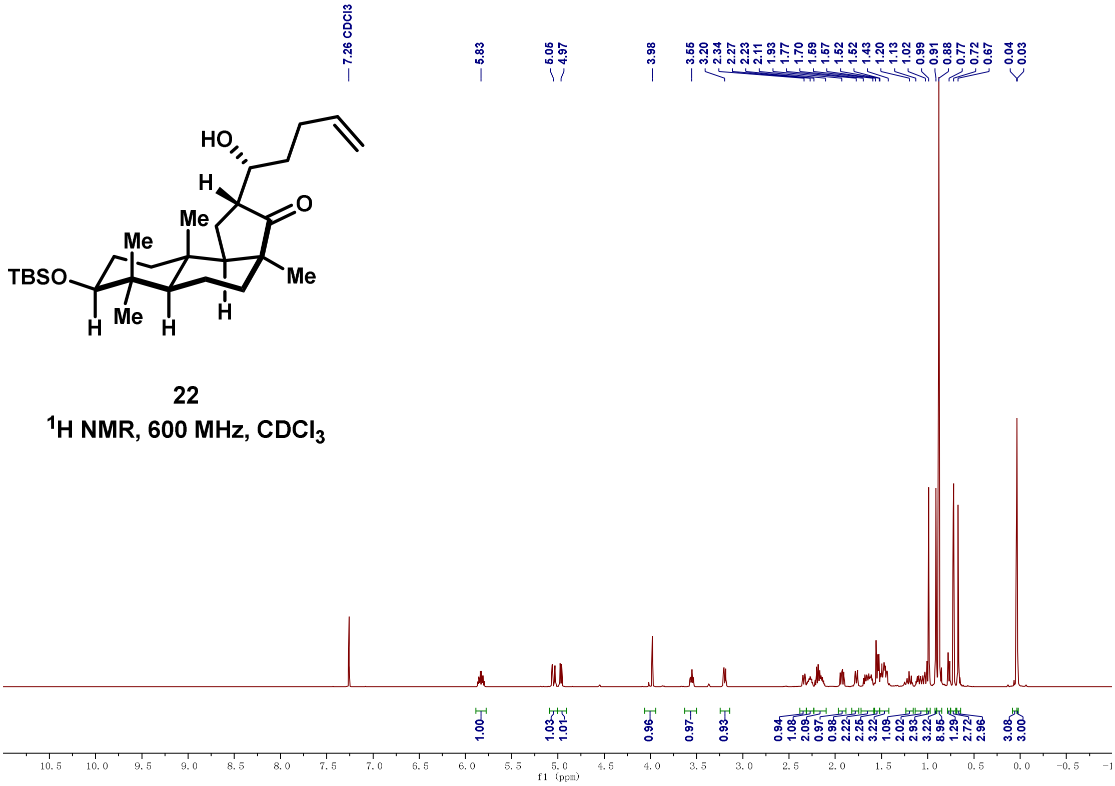

22

^{1}H NMR, 600 MHz, $CDCl_3$

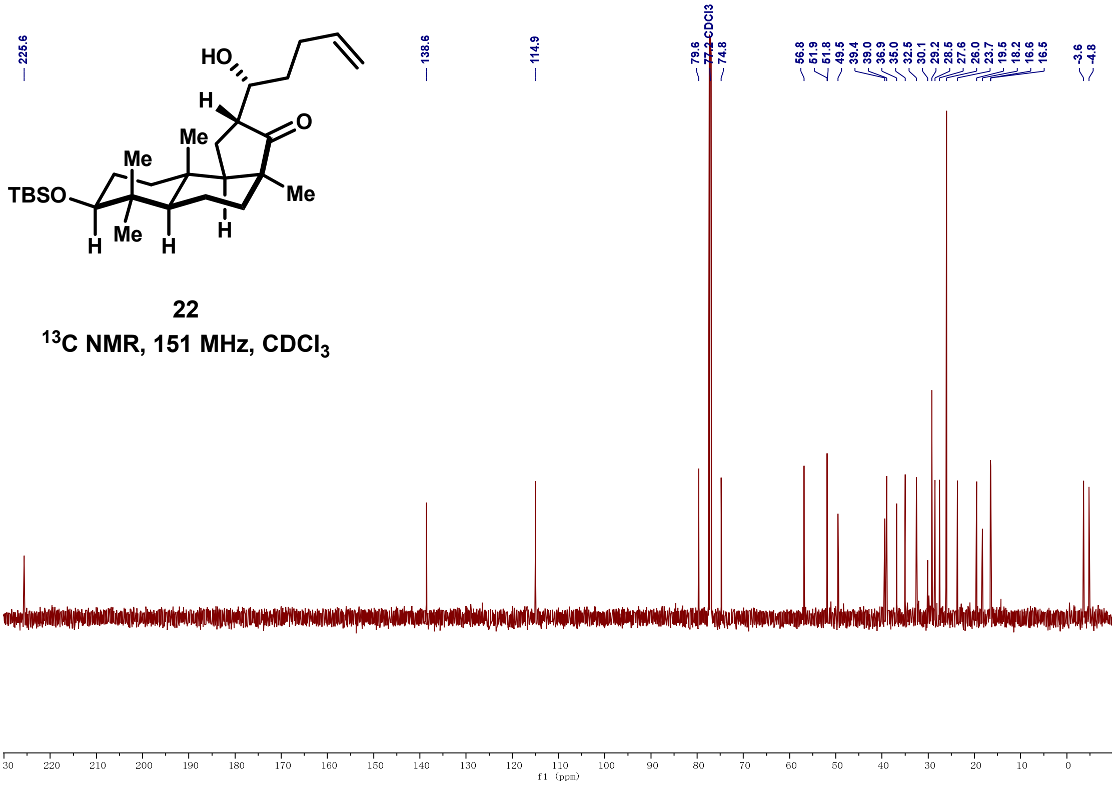

22

^{13}C NMR, 151 MHz, $CDCl_3$

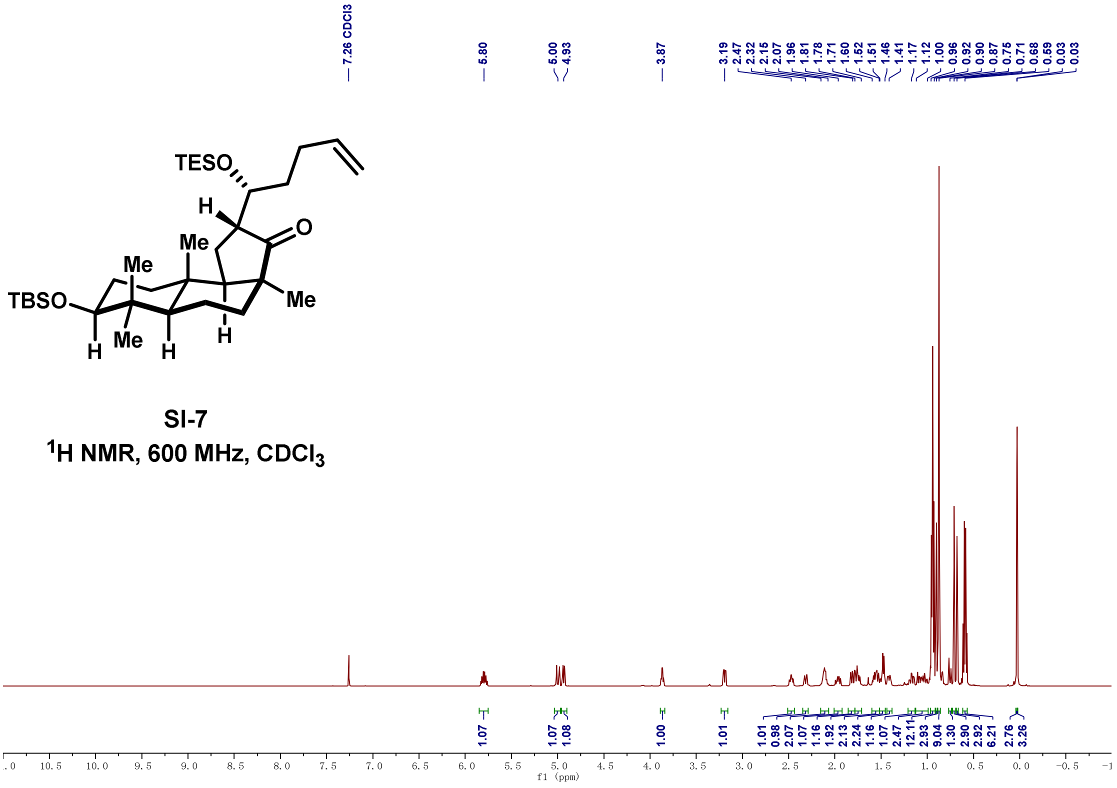

SI-7

^{1}H NMR, 600 MHz, $CDCl_3$

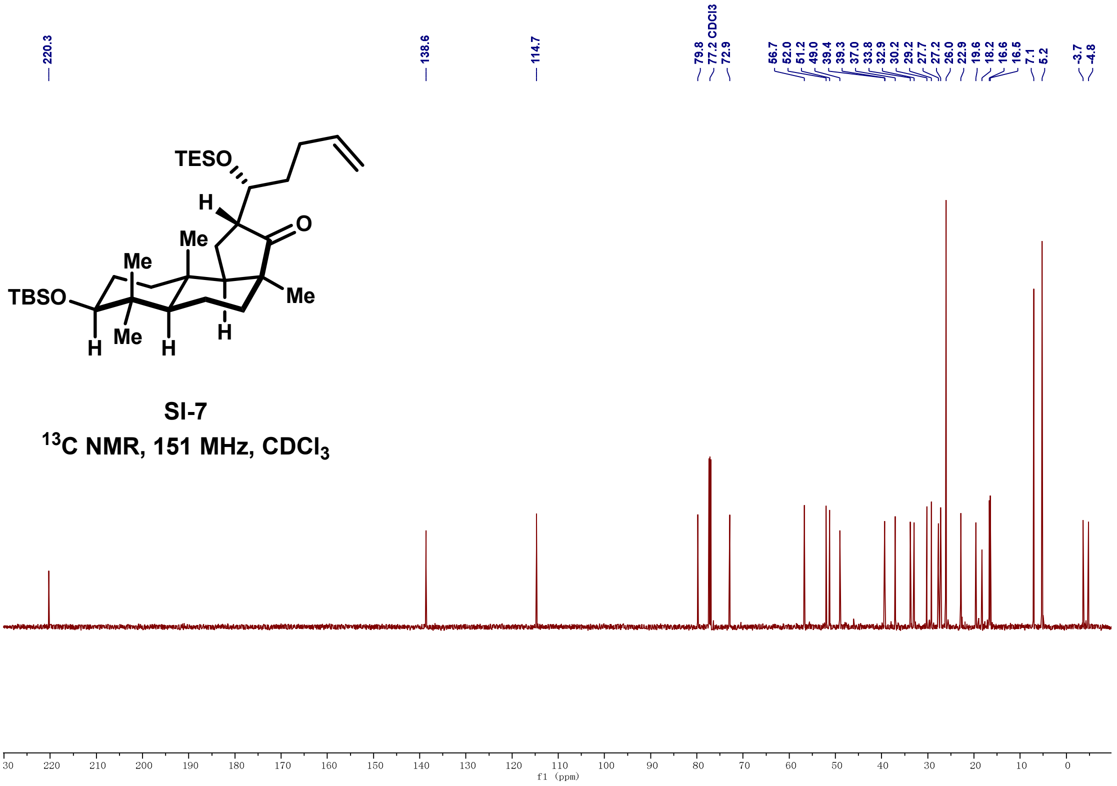

SI-7

^{13}C NMR, 151 MHz, $CDCl_3$

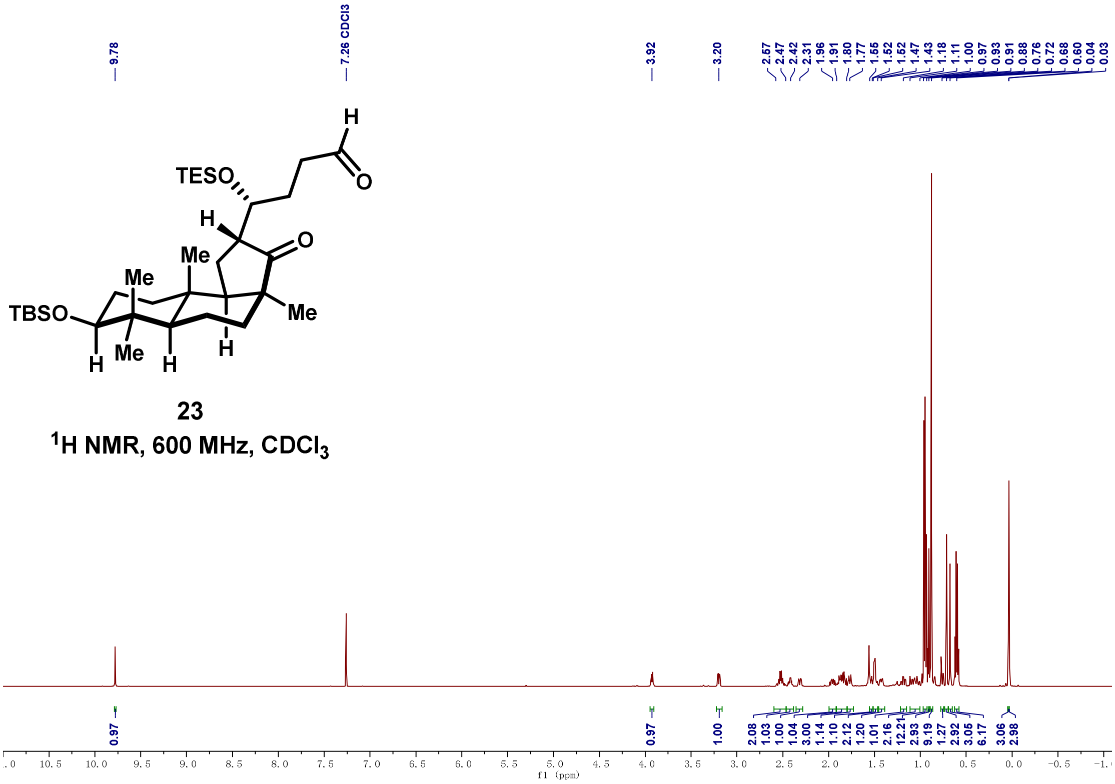

23
^{1}H NMR, 600 MHz, $CDCl_3$

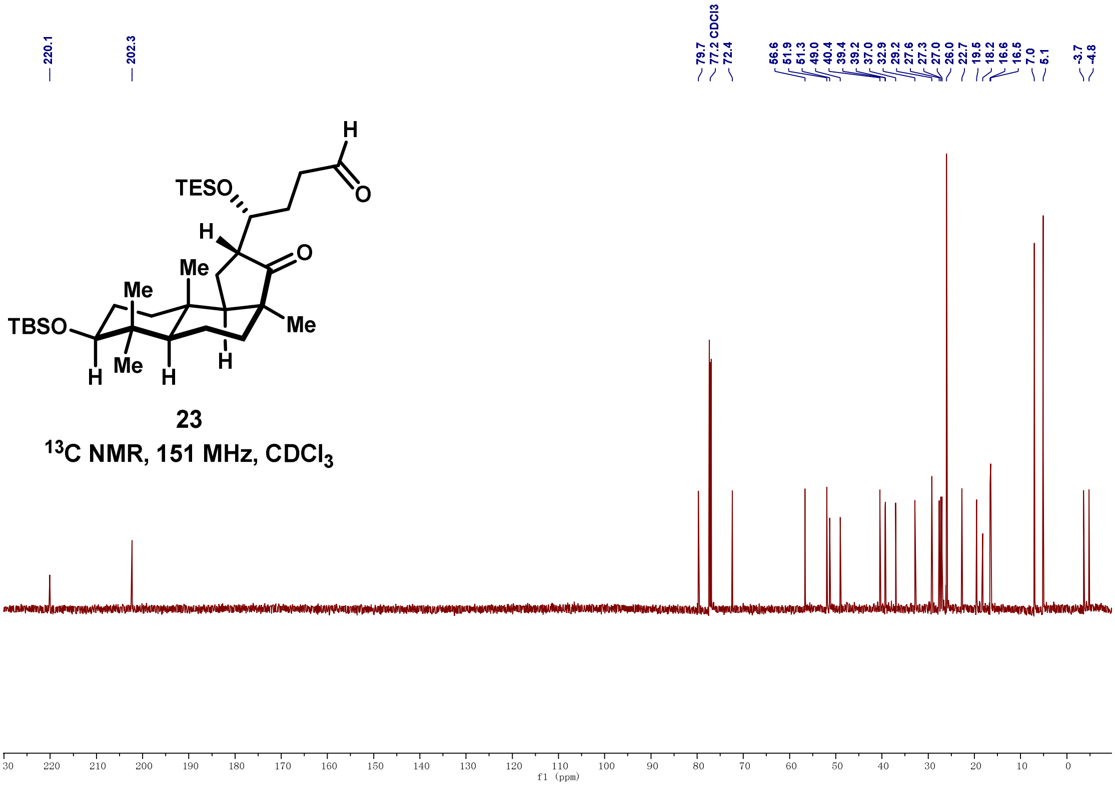

23
^{13}C NMR, 151 MHz, $CDCl_3$

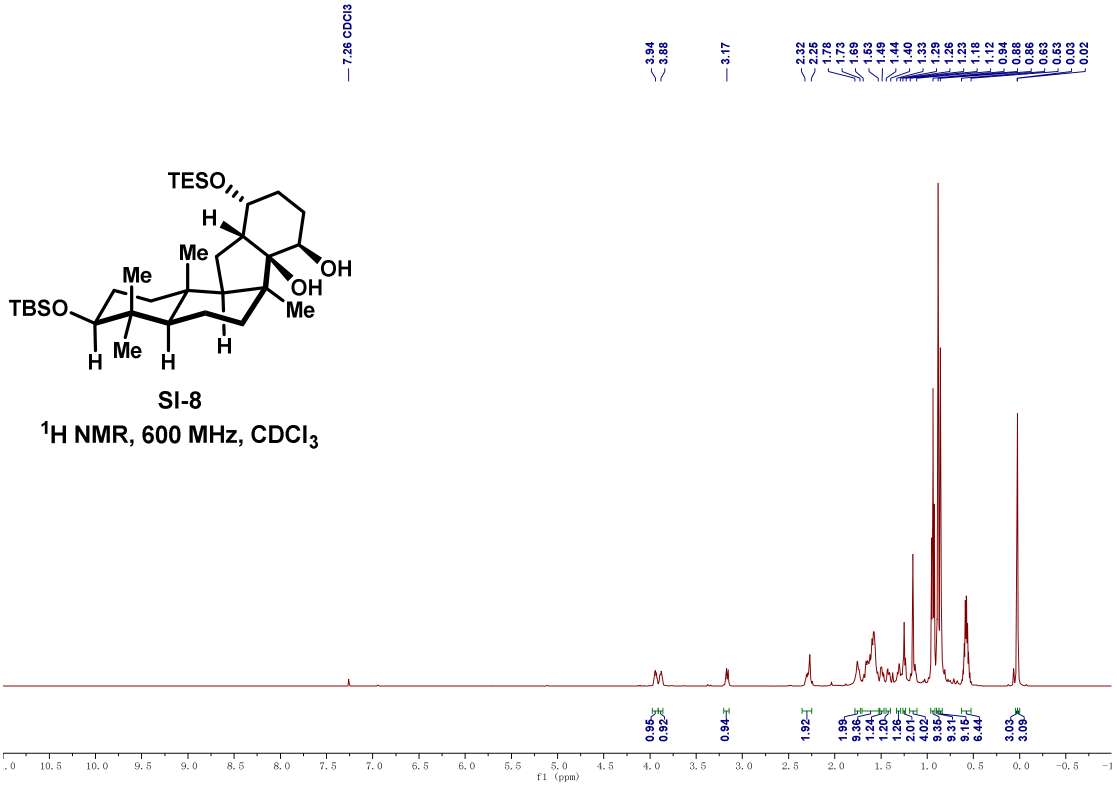

SI-8

^{1}H NMR, 600 MHz, $CDCl_3$

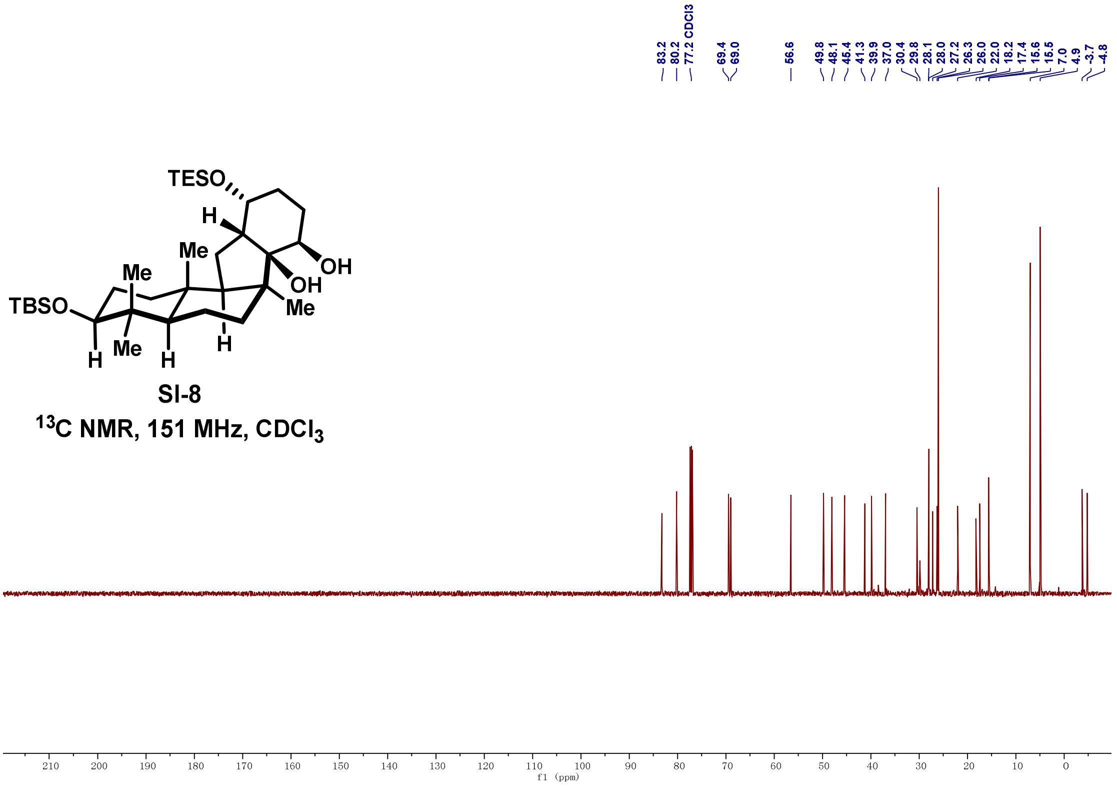

SI-8

^{13}C NMR, 151 MHz, $CDCl_3$

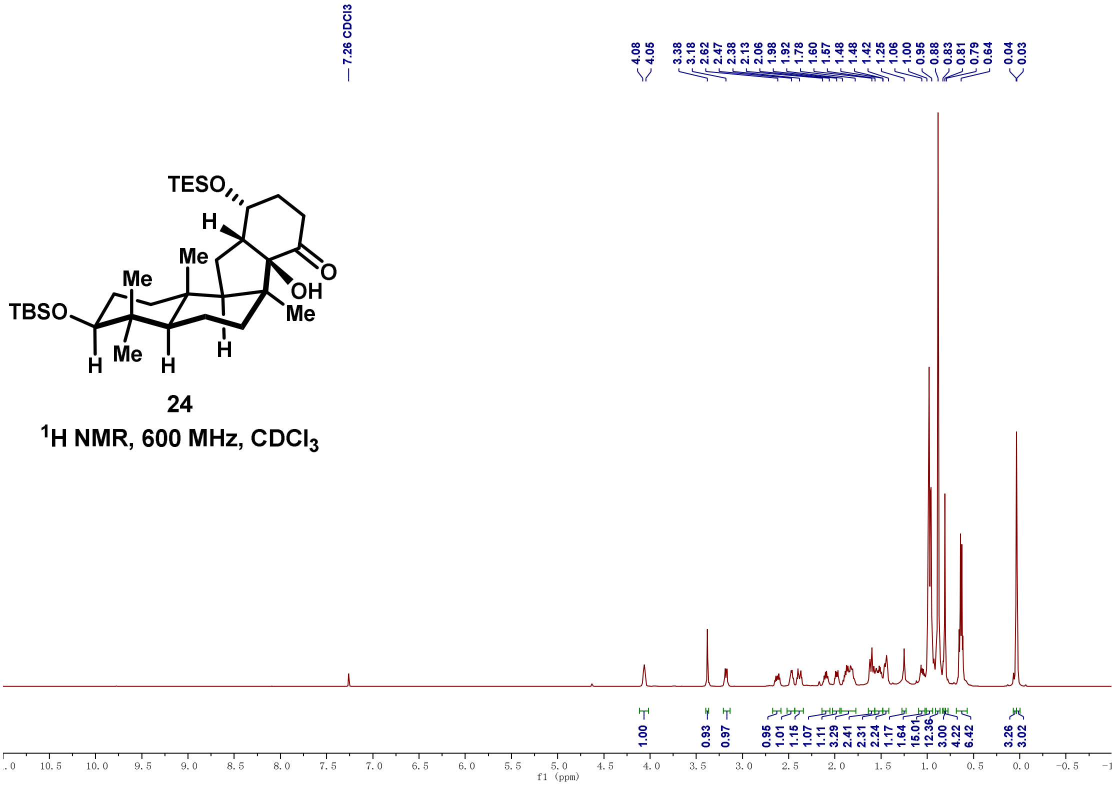

TESO
H
Me
Me
O
OH
TBSO
Me
Me
H
H
H
24
^{1}H NMR, 600 MHz, $CDCl_3$
7.26 CDCl3
4.08
4.06
3.38
3.18
2.62
2.47
2.38
2.13
2.06
1.98
1.92
1.78
1.60
1.57
1.48
1.48
1.42
1.25
1.06
1.00
0.96
0.88
0.83
0.81
0.79
0.64
0.04
0.03
1.00
0.93
0.97
0.96
1.01
1.16
1.07
1.11
3.29
2.41
2.31
2.24
1.17
1.64
15.01
12.36
3.00
4.22
6.42
3.26
3.02
f1 (ppm)

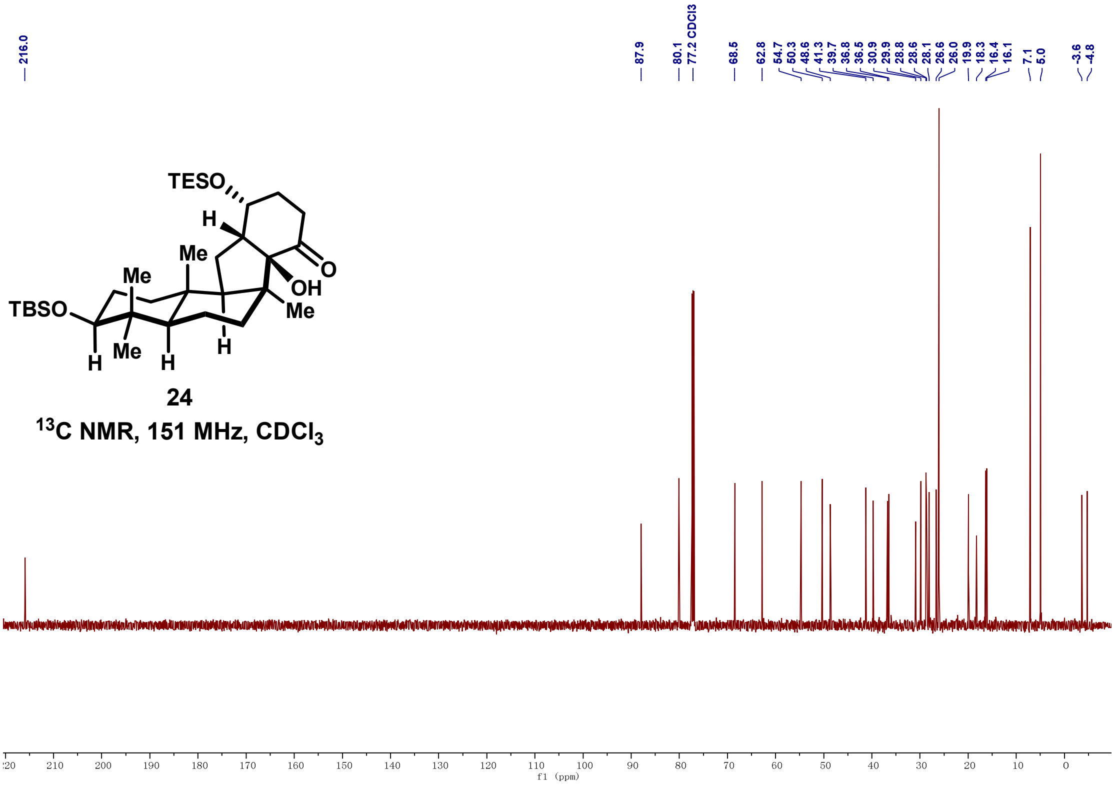

TESO
H
Me
Me
O
OH
TBSO
Me
Me
H
H
H
24
^{13}C NMR, 151 MHz, $CDCl_3$
216.0
87.9
80.1
77.2 CDCl3
68.5
62.8
54.7
50.3
48.6
41.3
39.7
36.8
36.5
30.9
29.9
28.8
28.6
28.1
26.6
26.0
19.9
18.3
16.4
16.1
7.1
5.0
-3.6
-4.8
f1 (ppm)

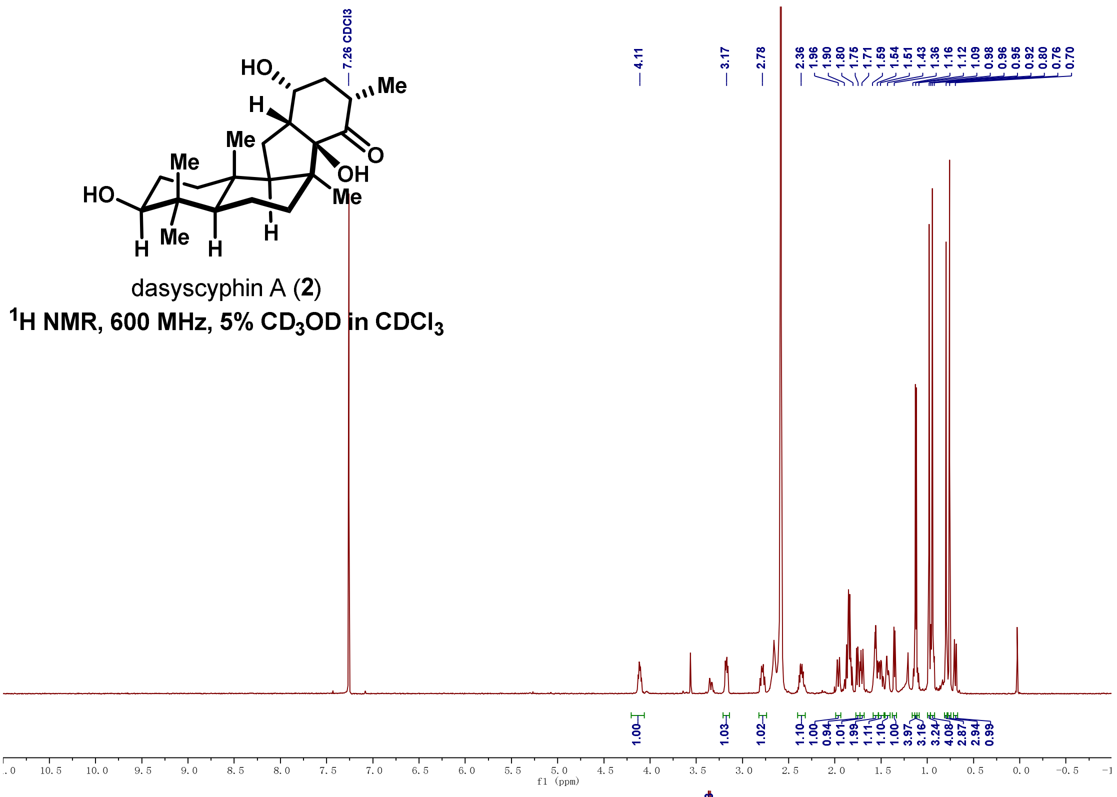

dasyscyphin A (**2**)

^{1}H NMR, 600 MHz, 5% CD_3OD in $CDCl_3$

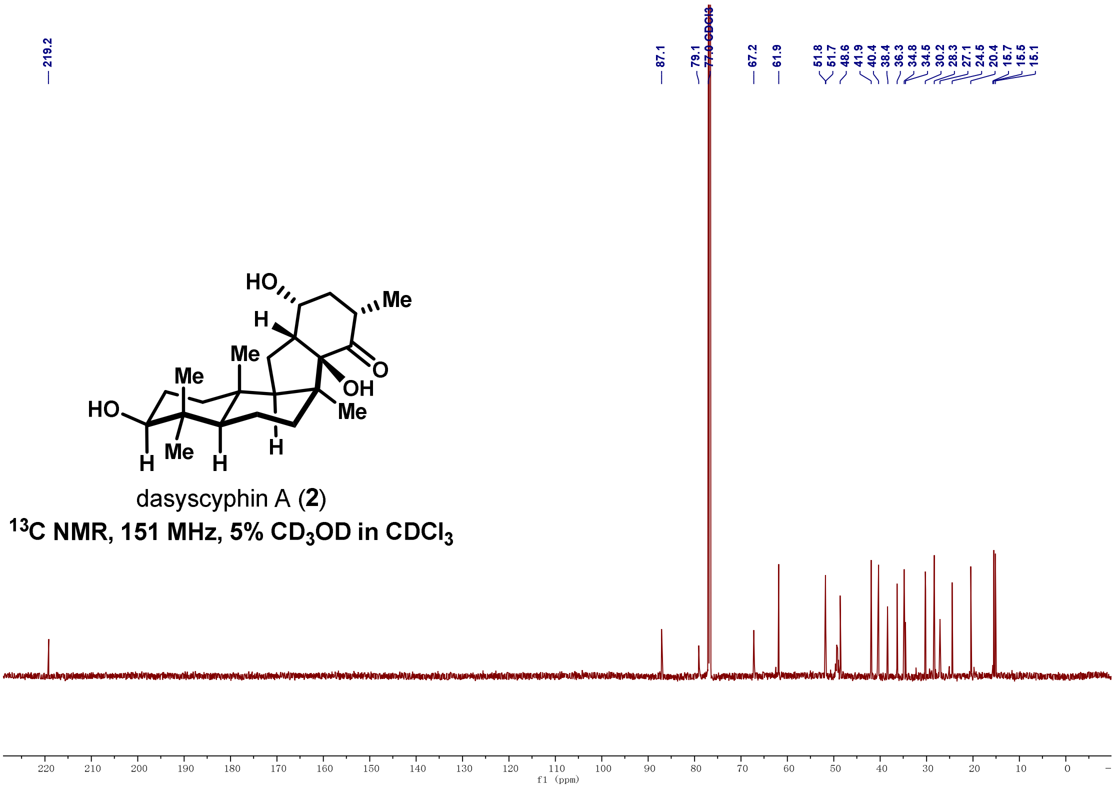

dasyscyphin A (**2**)

^{13}C NMR, 151 MHz, 5% CD_3OD in $CDCl_3$
